# RNA-Sequence Analysis of Primary Alveolar Macrophages after *In Vitro* Infection with Porcine Reproductive and Respiratory Syndrome Virus Strains of Differing Virulence

**DOI:** 10.1371/journal.pone.0091918

**Published:** 2014-03-18

**Authors:** Bouabid Badaoui, Teresa Rutigliano, Anna Anselmo, Merijn Vanhee, Hans Nauwynck, Elisabetta Giuffra, Sara Botti

**Affiliations:** 1 Parco Tecnologico Padano, Via Einstein, Lodi, Italy; 2 Laboratory of Virology, Faculty of Veterinary Medicine, Ghent University, Ghent, Belgium; University of Florida College of Medicine, United States of America

## Abstract

Porcine reproductive and respiratory syndrome virus (PRRSV) mainly infects porcine alveolar macrophages (PAMs), resulting in porcine reproductive and respiratory syndrome (PRRS) in pigs. Most of the transcriptomic studies on PAMs infected with PRRSV conducted thus far have made use of microarray technology. Here, we investigated the transcriptome of PAMs *in vitro* at 12 h post-infection with two European PRRSV strains characterized by low (Lelystad, LV) and high (Lena) virulence through RNA-Seq. The expression levels of genes, isoforms, alternative transcription start sites (TSS) and differential promoter usage revealed a complex pattern of transcriptional and post-transcriptional gene regulation upon infection with the two strains. Gene ontology analysis confirmed that infection of PAMs with both the Lena and LV strains affected signaling pathways directly linked to the innate immune response, including interferon regulatory factors (IRF), RIG1-like receptors, TLRs and PKR pathways. The results confirmed that interferon signaling is crucial for transcriptional regulation during PAM infection. *IFN-β1* and *IFN-αω*, but not *IFN-α*, were up-regulated following infection with either the LV or Lena strain. The down-regulation of canonical pathways, such as the interplay between the innate and adaptive immune responses, cell death and *TLR3*/*TLR7* signaling, was observed for both strains, but Lena triggered a stronger down-regulation than LV. This analysis contributes to a better understanding of the interactions between PRRSV and PAMs and outlines the differences in the responses of PAMs to strains with different levels of virulence, which may lead to the development of new PRRSV control strategies.

## Introduction

Porcine reproductive and respiratory syndrome (PRRS) is a viral disease that causes substantial economic losses to the swine industry [1], [2]. The PRRS virus (PRRSV) consists of a positive-sense single-stranded RNA molecule that is14.5 kb in length and has nine open reading frames. PRRSV strains can be divided into two groups, the European and North American genotypes, based on genetic and antigenic properties [3]. The European PRRSV genotype is divided into three subtypes: the mild pan-European subtype 1 (prototype virus: Lelystad virus, LV) and the more virulent eastern European subtypes 2 and 3 (prototype virus: Lena) [4]. The Lena virus has been found to be more virulent than the Lelystad virus [5]. Infection studies showed that the expression levels of *IFN-α*, *IL-10*, *IL-12* and *TNF-α* were higher in Lena-infected pigs than in LV-infected pigs [6]–[8]. Differences in virulence between the PRRSV strains are related to their immunomodulatory properties and replication capacity [9], [10]. Nevertheless, Morgan et al. [11] found that the *“SU1-bel, subtype 3”*strain from Belarus exhibited high virulence as a consequence of an enhanced inflammatory response, rather than an increased replication capacity.

PRRSV induces a strong humoral response; however, this response is not effective at controlling the virus, and a persistent infection frequently develops [12], [13]. The genetic diversity of PRRSV strains has been extensively characterized, and the correlation between their genetic diversity and geographical/temporal distances has been investigated to reveal the mechanisms of viral spreading [14]–[16]. Many strategies for controlling PRRSV transmission have been proposed but have generally shown little success, which has stimulated the search for new ways to control PRRSV transmission, including the possibility of using genetic breeding to achieve resistance to PRRSV [17].

Pulmonary alveolar macrophages (PAMs) are the main target cells for PRRSV infection, and many gene expression studies have explored the immune response of PAMs to PRRSV. Such studies have shown that the expression levels of *MX1*, *USP*, *IFN-β*, *IL-10* and *TNF-α* are affected by PRRSV infection [18], [19]. Overall, these analyses suggest that PRRSV subverts host defenses by inhibiting the expression of pro-inflammatory cytokines [20]–[22] and stimulating weak production of *IFN-α* [reviewed in 23].

RNA-Seq is increasingly being used to study gene expression, as it provides unbiased profiles, compared to microarrays [24], [25], and can be extremely accurate if a sufficient level of coverage is obtained [26]. Validation techniques, such as qPCR [27] and spike-in RNA [28], have corroborated the accuracy of RNA-Seq. One advantage of RNA-Seq over microarrays is that, in addition to gene expression, RNA-Seq data can be used to identify transcription start sites (TSS), splicing variants and differential promoter usage [28]. Annotations of the human genome suggest that a high proportion of protein-coding genes exhibit alternative promoters, and aberrant promoter usage has been found to be associated with various diseases [29].

In this study, RNA-Seq was used to characterize gene expression, splice variants, TSSs and differential promoter usage in PAMs infected with either LV (subtype 1, low virulence) or Lena (subtype 3, high virulence). The PAM signaling pathways that were affected by PRRSV infection were investigated and were found to differ following infection with the two PRRSV strains. The obtained data confirmed that many signaling pathways are potentially involved in the host immune response to PRRSV infection and that the regulation of interferon signaling is strongly modulated during PAM infection. In contrast to infection with LV, infection with Lena down-regulated the pathways involved in the interplay between the innate and adaptive immune response, cell death and *TLR3*/*TLR7* signaling. Additionally, we also highlight many differences in splicing isoforms, TSSs and differential promoter usage between LV and Lena infections.

## Materials and Methods

### Animals, cells and library preparation

Pulmonary alveolar macrophages were collected from three 3-week-old piglets, which were the offspring of hybrid sows (JSR Genepacker 90 English: Landrace x Large White) and Pietrain boars from a PRRS-negative herd. The PRRSV- and PCV2-negative status of the piglets was confirmed via an immunoperoxidase monolayer assay (IPMA) and PCR, respectively. The piglets were treated daily with 1 ml of enrofloxacin (5% solution) and 1 ml of lincospectin/spectinomycin (5 or 10% solution) for 3 days to eliminate bacterial pathogens and then were sacrificed one week later via intravenous injection with a lethal dose of Na-pentobarbital (20 ml/animal) in the jugular vein. The sacrifice procedure was carried out by a technician certified in Laboratory Animal Science (Felasa Category C). The PAMs were collected by broncho-alveolar lavage and frozen in liquid nitrogen as described by Wensvoort et al. [30].

The animals were housed at the Faculty of Veterinary Medicine of Ghent University (Belgium). All experimental procedures and the sacrifice of the animals were conducted in compliance with the relevant legislation on animal experiments (EU Directive 2010/63) and with the approval of the local ethical committee.

Prior to infection, the PAMs were thawed and cultured for 48 h, as described by Delputte et al. [31]. The primary culture from each of the three animals was split into three fractions: one fraction was infected at a multiplicity of infection (MOI) of 0.5 with the LV strain; the second was inoculated at a MOI of 0.5 with the Lena strain; and the third was maintained as a control (mock inoculated). The cells were collected at 12 h post-infection for transcriptome analysis. All of the infection experiments and controls were performed in duplicate. One of each duplicate was used to assess the percentage of infected cells. After 12 h, the cells were fixed in acetone-100% methanol at −20°C, and the infected cells were detected via immunoperoxidase staining with the monoclonal anti-nucleocapsid protein antibody P3/27 (48), followed by incubation with a horseradish peroxidase-labeled goat anti-mouse secondary antibody and development with a substrate solution containing 3-amino-9-ethylcarbazole. Viral antigen-positive cells and total cells were counted using an Olympus light microscope (Olympus Optical Co., Hamburg, Germany), and the percentage of infected cells was calculated. Three microscopic fields and a minimum of 200 cells per field were counted for each experimental condition.

Total RNA was extracted from the PAMs using TRIzol (Invitrogen Life Technologies, Milan-Italy) and RNeasy columns (Qiagen). RNA quality was assessed via microcapillary electrophoresis on an Agilent 2001 Bioanalyzer (Agilent Technologies) with RNA 6000 Nanochips. RNA was quantified using spectrophotometry (ND-1000; NanoDrop Technologies).

cDNA libraries were constructed using RNAfrom the LV-, Lena- or mock-infected PAMs. A total of nine cDNA libraries were obtained using TruSeq Sample Prep Kits (Illumina Inc., San Diego, CA) and were sequenced via 2×100 paired-end sequencing on an IlluminaHiSeq 2000 instrument. The obtained sequences were submitted to the Sequence Read Archive (SRA) at NCBI (SRX352447).

The Laboratory of Virology has been acknowledged by the Belgian Federal Agency for the Safety of the Food Chain (FASFC) for its work with experimental animals, including pigs (national approval number LA1400076). As euthanasia was the only action performed on the animals, no approval was required by the local ethical committee (Article 1, 5f, EU Directive 2010/63).

### Quality check, mapping, assembly and visualization of the sequenced reads

Sequence visualization and statistical analyses were performed with FASTQC (http://www.bioinformatics. bbsrc.ac.uk/projects/fastqc). Sickle software (https://github.com/najoshi/sickle) was employed to retrieve the paired sequences with quality scores above a threshold of 20 and a length greater than 50 bp. The reads passing the quality criteria were processed and aligned to the reference pig genome (Build Sus_scrofa.Sscrofa10.2.71.) using TopHat v2.0.8 [32], and a maximum of 2 mismatches were allowed when mapping the reads to the reference genome. The default parameters for TopHat were used [32]. The mapping results were then used to identify “islands” of expression, which can be interpreted as potential exons. The mapping results were visualized using the UCSC genome browser [33] and the Integrative Genomics Viewer (IGV) software, which is available at http://www.broadinstitute.org/igv/.

### Transcript assembly and abundance estimation

Transcript assembly and abundance estimation were performed using two methods: one for the genes and the other for the remaining features (isoforms, TSS and promoters).

The aligned reads were further processed using Cufflinks v2.1.1 [34] (Figure S4-A) to infer the splicing structure of each gene using the optimal reconstruction of the gene model, which was determined using a rigorous statistical model. The reads were assembled into transcripts by forcing Cufflinks to use not just the existing gene annotations during the assembly of transcripts but instead to construct the transcripts based on both the reference gene annotations and the sequence data. This approach allows Cufflinks to identify new potential alternative transcription and splicing events. Confidence intervals for the FPKM estimates were calculated using a Bayesian inference method [35]. Once all of the short read sequences were assembled with Cufflinks, the output files were merged via the Cuffmerge function and processed using Cuffcompare, along with a reference GTF annotation file downloaded from the Ensembl database.

The HTseq python package (http://www-huber.embl.de/users/anders/HTSeq/doc/count.html) was employed to generate an unambiguous table of counts per gene to estimate expression levels. The HTseq-count function was employed, with ‘union’ in overlapping mode and ‘gene’ as a feature.

### Differential expression analysis

The cells obtained from each pig were divided into three batches to represent the two treatment conditions (LV-infected orLena-infected) and the control (mock-infected). To remove baseline differences between the pigs, a paired design was employed, in which treatment values were subtracted from control values for each pig prior to the computation of the average and variance. Differential expression analysis was performed using the EdgeR package [36], which employs the table of counts per gene generated by HTSeq as an input.

Using edgeR, a general linear model was generated to test for a treatment (infection with Lena or LV) effect. This method is based on the negative binomial distribution and uses the conditional weighted likelihood to moderate the level of overdispersion across the sequences. Three pairwise comparisons of gene expression levels were performed for the three combinations of treatments: i) LV *vs.* mock, ii) Lena *vs.* mock and iii) LV *vs.* Lena.

The Cuffdiff function was used to assess the presence and frequency of different isoforms, TSSs and differential promoter usage. Briefly, Cuffdiff starts by modeling the variability in the fragment count for each gene across the replicates corresponding to the mock, LV and Lena samples. Second, the counts for each isoform are estimated in each replicate, taking into consideration the uncertainty arising from ambiguously mapped reads. Finally, Cuffdiff estimates the count variances for each transcript in each sample, and this information is used in statistical testing to report the differentially expressed transcripts, including the isoforms, TSSs and differential promoter usage [34]. Cuffdiff was used to perform three pairwise comparisons of the observed isoforms, TSSs and promoter usage: i) LV *vs.* mock ii) Lena *vs.* mock and iii) LV *vs.* Lena. For all of the analyses, a feature was considered significant if the false discovery rate (FDR) was less than 0.05 and the fold change (FC) difference was greater than or equal to 1.5.

### Functional analysis of gene lists through Ingenuity Pathways Analysis (IPA)

IPA (http://www.ingenuity.com) was used to identify the most significant molecular networks, biological functions and canonical metabolic pathways. The p-values associated with the biological processes or pathway annotation were calculated according to the right-tailed Fisher's Exact Test. Affected genes were first matched to their corresponding gene names in IPA. The obtained lists were then subjected to IPA to reveal the canonical pathways and biological functions that were significantly associated with the gene lists, using the default “universe” of genes and endogenous chemicals in the IPA library.

## Results

### 1. Expressed and differentially expressed genes, isoforms and TSSs, as well as differential promoter usage,thein LV *vs.* mock, Lena *vs.* mock and LV *vs.* Lena analyses

The percentages of PAMs infected by LV and Lena were 21% and 16% on average, respectively. This result was confirmed using quantitative PCR; the threshold cycle (Ct) values obtained were 15.6 and 20.5 for LV and Lena, respectively.

Nine libraries obtained from PAMs corresponding to LV (3 samples), Lena (3 samples) and mock infections (3 samples) were sequenced. The mean numbers of reads produced per strand were 14,483,298, 17,381,381 and 10,473,546 for the LV, Lena and Mock groups, respectively (Table S1-A). Data filtering using FASTQC and Sickle excluded between 11% and 17% of the reads (Table S1-B). Between 73% and 87% of the total paired reads passing the quality test were mapped to the reference pig genome (Build Sus_scrofa.Sscrofa10.2.71).

**Figure 1:**
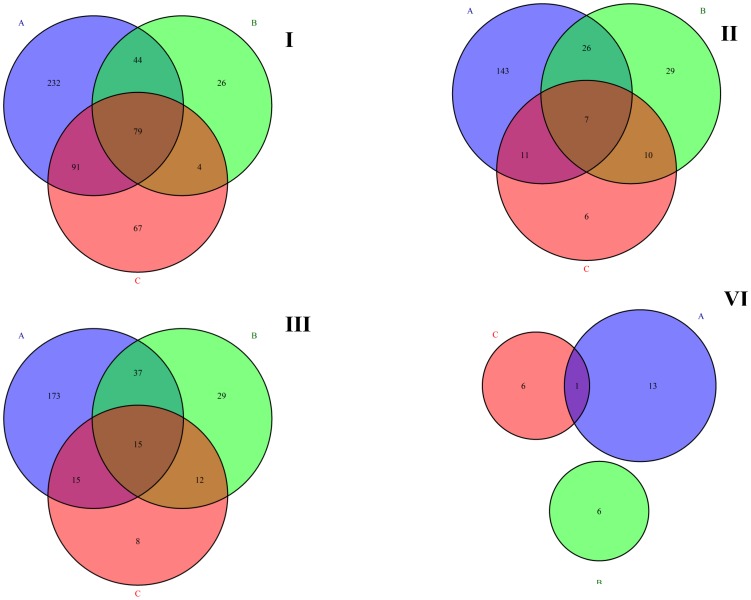
Venn diagram illustrating the significantly affected genes (I), isoforms (II), TSSs (III) and differential promoter use (VI) found in the LV *vs.* mock (A), Lena *vs.* mock (B) and LV *vs.* Lena (C) comparisons. **I.** Venn diagram illustrating the significantly affected genes found in the LV *vs.* mock (A, 446), Lena *vs.* mock (B, 153) and LV *vs.* Lena (C, 241) comparisons. **II.** Venn diagram illustrating the significantly affected isoforms found in the LV *vs.* mock (A, 187), Lena *vs.* mock (B, 72) and LV *vs.* Lena (C, 34) comparisons. **III.** Venn diagram illustrating the significantly affected TSSs found in the LV *vs.* mock (A, 240), Lena *vs.* mock (B, 93) and LV *vs.* Lena (C, 50) comparisons. **VI.** Venn diagram illustrating the significantly affected promoters found in the LV *vs.* mock (A, 14), Lena *vs.* mock (B, 6) and LV *vs.* Lena (C, 7) comparisons. The blue circle, “A”, corresponds to the LV *vs.* mock case (I. significantly affected genes, II. significantly affected isoforms, III. significantly affected TSSs and VI. significantly affected promoters). The green circle, “B”, corresponds to the Lena *vs.* mock case (I. significantly affected genes, II. significantly affected isoforms, III. significantly affected TSSs and VI. significantly affected promoters). The purple circle, “C”, corresponds to the LV *vs.* Lena case (I. significantly affected genes, II. significantly affected isoforms, III. significantly affected TSSs and VI. significantly affected promoters).

In total, 29,900 annotated genes and 58,347 isoforms were expressed in both the infected and non-infected PAMs. Of these genes, 0.46% were expressed at more than 10,000 FPKM, 0.6% between 1000 and 10,000 FPKM, 3.14% between 100 and 1000 FPKM and 40.64% between 1 and 100 FPKM. The genes with the highest expression were XLOC_009444 (14,122,900 FPKM in the LV-infected cells), XLOC_027735 (13,467,000FPKM in the Lena-infected cells) and SCARNA16 (13,133,300 FPKM in the LV-infected cells). The differentially expressed features and the intersection of the differentially expressed genes, isoforms, TSSs and differential promoter usage observed in the LV *vs.* mock, Lena *vs.* mock and LV *vs.* Lena comparisons are shown in Figure 1 (1.I, 1.II, 1.III and 1.IV).

#### 1.1. Canonical pathways and biological functions differentially expressed in the LV- vs. mock-infected samples

**Figure 2:**
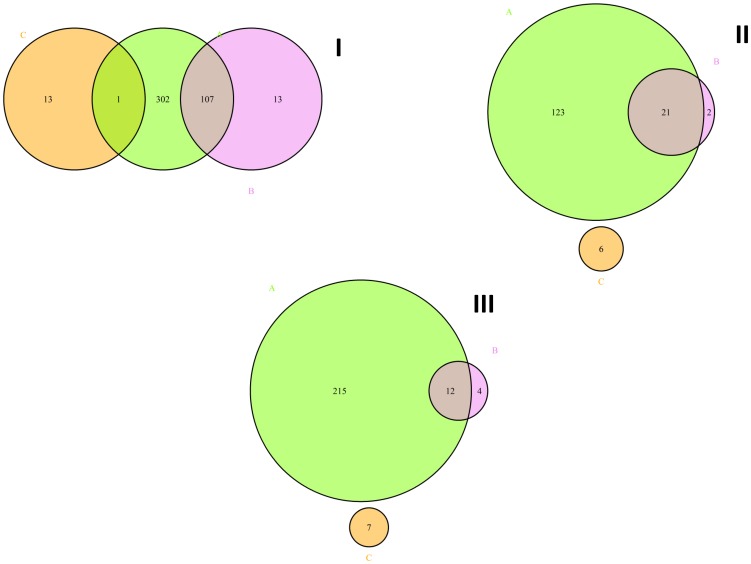
Venn diagram illustrating the significantly affected genes (A, chartreuse color), isoforms (B, violet color) and differential promoter use (C, orange color) found in the LV *vs.* mock (I), Lena *vs.* mock (II) and LV *vs.* Lena (III) comparisons. **I.** Venn diagram illustrating the significantly affected genes (A, 446), isoforms (B, 187) and differential promoter use (C, 14) found in the LV *vs.* mock comparison. **II.** Venn diagram illustrating the significantly affected genes (A, 153), isoforms (B, 72) and promoters (C, 6) found in Lena *vs.* mock comparison. **III.** Venn diagram illustrating the significantly affected genes (A, 241), isoforms (B, 34) and promoters (C, 7) found in LV *vs.* Lena comparison. The chartreuse circle, “A”, corresponds to the differentially expressed genes (I. LV *vs.* mock, II. Lena *vs.* mock and III. LV *vs.* Lena). The violet circle, “B”, represents the differentially expressed isoforms (I. LV *vs.* mock, II. Lena *vs.* mock and III. LV *vs.* Lena). The orange circle, “C”, corresponds to the differentially used promoters(I. LV *vs.* mock, II. Lena *vs.* mock and III. LV *vs.* Lena).

A total of 446 differentially expressed genes were identified between the mock-infected and LV-infected PAMs (Table S2). Among these genes, 107 presented at least two isoforms that were differentially expressed between the mock- and LV-infected PAMs, and one was differentially regulated at the promoter level (Figure 2.I). In total, 413 genes could be mapped to the IPA database to assign gene ontology. The top five canonical pathways identified as being differentially expressed in the LV *vs.* mock via IPA were “Interferon Signaling”, “Activation of IRF by Cytosolic Pattern Recognition Receptors”, the “Role of Pattern Recognition Receptors in Recognition of Bacteria and Viruses”, the “Role of PKR in Interferon Induction and Antiviral Response” and “Retinoic Acid Mediated Apoptosis Signaling” (Table 1A). All of these pathways are interconnected and are directly involved in the immune response to viral infection. In the infected cells, the “Interferon Signaling” pathway, which is central to the innate defense mechanisms of the host against viral infection, included 12 up-regulated genes: *IFIT1*, *IFIT3*, *IFITM1*, *IFNβ1*, *IRF1*, *JAK2*, *MX1*, *OAS1*, *PSMB8*, *SOCS1*, *STAT1* and *STAT2*. An additional 14 up-regulated genes were involved in the activation of IRF by the cytosolic pattern recognition receptor pathway (*ADAR*, *DDX58*, *DHX58*, *IFIH1*, *IFIT2*, *IFNβ1*, *IL10*, *IRF7*, *ISG15*, *NFKBIA*, *STAT1*, *STAT2*, *TNF* and *ZBP1*). The “Role of Pattern Recognition Receptors in Recognition of Bacteria and Viruses” pathway, which generally leads to the serine phosphorylation of *IRF3* and *IRF7* [37], involved *IRF7*, but not *IRF3*. The “Role of PKR in Interferon Induction and Antiviral Response” and “Retinoic Acid Mediated Apoptosis Signaling” pathways were up-regulated and shared four genes (*CASP8*, *BID*, *IFNβ1* and *IRF1*).

**Table 1:** Lists of the 5 top canonical pathways and the corresponding affected genes in the LV *vs.* mock (A), Lena *vs.* mock (B) and LV *vs.* Lena (C) comparisons.

A. LV *vs.* mock infection			
Ingenuity Canonical Pathways	−log(p-value)*	Ratio£	Molecules&
Interferon Signaling	1.08E+01	3.87E-01	IFIT3,SOCS1,IFIT1,OAS1,MX1,IFNB1, IFITM1,STAT2,PSMB8,JAK2,STAT1,IRF1
Activation of IRF by Cytosolic Pattern Recognition Receptors	9.97E+00	2.64E-01	DHX58,IL10,ZBP1,IFNB1,ADAR,ISG15, IFIH1,IRF7,NFKBIA,DDX58,STAT2,IFIT2, STAT1,TNF
Role of Pattern Recognition Receptors in Recognition of Bacteria and Viruses	8.55E+00	1.78E-01	OAS1,OAS2,IL10,PIK3R1,IFNB1,RNASEL,IFIH1,IRF7,DDX58,TLR7,CASP1,IL1B,PIK3CD, EIF2AK2,TLR3,TNF
Role of PKR in Interferon Induction and Antiviral Response	7.03E+00	2.5E-01	NFKBIA,IFNB1,BID,EIF2AK2,TLR3,STAT1, CASP8,RNASEL,TNF,IRF1
Retinoic Acid Mediated Apoptosis Signaling	6.96E+00	2.16E-01	BID,CASP8,IFNB1,IRF1,PARP9,PARP11, PARP14,TIPARP,TNFRSF10A,TNFSF10, ZC3HAV1

B. Lena *vs.* mock infection			
Activation of IRF by Cytosolic Pattern Recognition Receptors	1.04E+01	1.89E-01	DHX58,IFIH1,IRF7,DDX58,ZBP1,IFNB1,ADAR, IFIT2,TNF,ISG15
Role of Pattern Recognition Receptors in Recognition of Bacteria and Viruses	8.09E+00	1.11E-01	IFIH1,OAS1,IRF7,OAS2,DDX58,IFNB1,PIK3CD, EIF2AK2,RNASEL,TNF
Role of Hypercytokinemia/hyperchemokinemia in the Pathogenesis of Influenza	8.02E+00	2.26E-01	CXCL10,IL8,CCR5,CCL4,CCL2,IFNB1,TNF
Interferon Signaling	8.02E+00	2.26E-01	IFIT3,SOCS1,IFIT1,OAS1,MX1,IFNB1, IFITM1
Role of RIG1-like Receptors in Antiviral Innate Immunity	4.89E+00	1.47E-01	DDX58,DHX58,IFIH1,IFNB1,IRF7

C. LV *vs.* Lena infection			
IL-15 Production	4.65E+00	2.17E-01	IL15,IFNB1,JAK2,STAT1,IRF1
TREM1 Signaling	4.54E+00	1.21E-01	IL8,TLR4,CCL2,TLR7,JAK2,TLR3,TNF
Communication between Innate and Adaptive Immune Cells	4.43E+00	9.76E-02	CXCL10,IL8,TLR4,IL15,TLR7,IFNB1,TLR3, TNF
Crosstalk between Dendritic Cells and Natural Killer Cells	4.35E+00	9.52E-02	TLR4,MICB,IL15,TLR7,IFNB1,TNFSF10,TLR3,TNF
Role of JAK2 in Hormone-like Cytokine Signaling	3.92E+00	1.56E-01	SOCS1,SOCS2,JAK2,STAT1,SIRPA

*probability that a canonical pathway is significantly involved in the analysis.

£ number of genes in the list involved in a certain canonical pathway divided by the number of genes in the IPA database involved in the same canonical pathway.

& genes involved in the corresponding canonical pathway.

**Figure 3:**
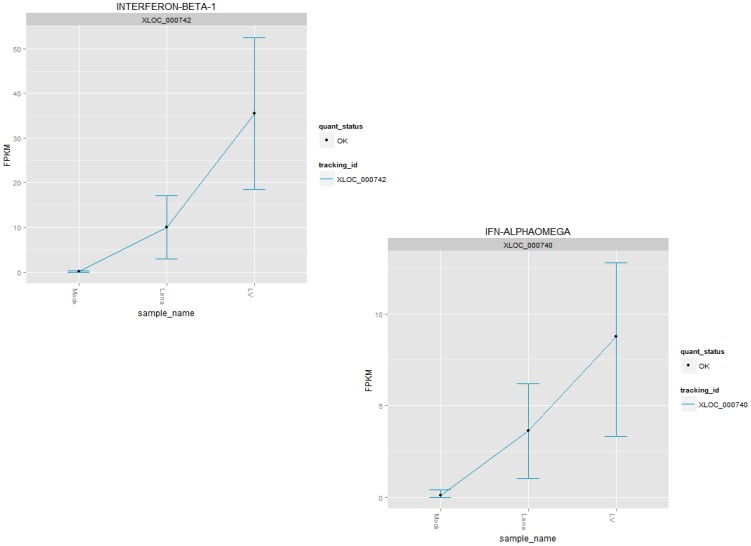
Gene expression of *IFN-β* and *IFN-αω* genes in FPKM (Y axis). Top left: *IFN-β* expression in the mock, LV and Lena groups. *IFN-β* was differentially expressed between both the LV and Lena groups when compared to the mock infection group. *IFN-β* was also expressed at a significantly higher level in the LV group than in the Lena group. Lower right: *IFN-αω* expression in the mock, LV and Lena groups. *IFN-αω* was differentially expressed between the LV and Lena groups from one side and the mock group from the other side. *IFN-β* was also expressed at a significantly higher level in the LV group than in the Lena group.

The *IFN-β* gene was common to all of the top canonical pathways, which is consistent with the previously reported fundamental role of this gene in the response of pigs to PRRSV (reviewed in [23]). Interestingly, *IFN-β* and *IFN*-αω were both up-regulated following LV and Lena infection, but the expression of *IFN-β* was higher in the PAMs infected with LV (Figure 3).

Among the top ten biological functions that differed between the LV-and mock-infected samples (Table 2A), the categories “Cell death of immune cells”, “Leukocyte infection”, other functions related to the hematological system (the numbers of leukocytes, mononuclear leukocytes, lymphocytes and T lymphocytes) and the “Cell death/survival” pathway were all up-regulated. Only two biological functions (apoptosis of phagocytes and cellular homeostasis) were down-regulated.

**Table 2:** Lists of affected biological functions and the corresponding affected genes in the LV *vs.* mock (A) and Lena *vs.* mock (B) comparisons.

A. LV *vs.* mock				
Function Annotation	p-Value*	Predicted Activation State£	Activation z-score&	Molecules§
**A. LV ** ***vs.*** ** mock**				
cell death of immune cells	1.89E-13	Increased	2.147	BID,CASP1, CASP8, CCL4, CCR5, CD274, CD38, CD5, DHX58, EIF2AK2, HAVCR2, HCAR2, HSH2D, IDO1, IFNB1, IL10, IL15, IL15RA, IL1B, IL27, IL7R, IRF1, IRF2, IRF4, JAK2, LGALS9, MCL1, MX1, NAMPT, NFKBIA, PARP14, PIK3CD, PIK3R1, PIM1, PMAIP1, PPBP, PRDM1, PYCARD, RHOH, RNASEL, SEMA4D, SOCS1, STAT1, TLR3, TNF, TNFAIP3, TNFSF10, TNFSF13B
quantity of leukocytes	2.62E-13	Increased	2.297	ABCA1, ARNTL, B3GNT5, BID, CASP8, CCL2, CCL3L1/CCL3L3, CCR2, CCR5, CD274, CD38, CD5, CXCL10, CYP27B1, DAPP1, DDX58, ELF1, ENTPD7, HAVCR2, HBEGF, HLA-B, IDO1, IFNB1, IL10, IL15, IL15RA, IL1B, IL27, IL7R, IRF1, IRF2, IRF4, ISG15, JAK2, LGALS9, MAEA, MAP3K8, MCL1, MXD1, NBN, NFKBIA, PDE4B, PIK3CD, PIK3R1, PIM1, PIM3, PLAC8, PML, PPM1D, PRDM1, PTPN13, PYCARD, RFX5, RHOH, RICTOR, SEMA4D, SOCS1, SOX4, STAT1, TCF4, TEC, TIMD4, TLR3, TNF, TNFAIP3, TNFRSF10A, TNFSF10, TNFSF13B, VCAM1
Cell death	2.62E-13	Increased	2.297	ABCA1, ARNTL, B3GNT5, BID, CASP8, CCL2, CCL3L1/CCL3L3, CCR2, CCR5, CD274, CD38, CD5, CXCL10, CYP27B1, DAPP1, DDX58, ELF1, ENTPD7, HAVCR2, HBEGF, HLA-B, IDO1, IFNB1, IL10, IL15, IL15RA, IL1B, IL27, IL7R, IRF1, IRF2, IRF4, ISG15, JAK2, LGALS9, MAEA, MAP3K8, MCL1, MXD1, NBN, NFKBIA, PDE4B, PIK3CD, PIK3R1, PIM1, PIM3, PLAC8, PML, PPM1D, PRDM1, PTPN13, PYCARD, RFX5, RHOH, RICTOR, SEMA4D, SOCS1, SOX4, STAT1, TCF4, TEC, TIMD4, TLR3, TNF, TNFAIP3, TNFRSF10A, TNFSF10, TNFSF13B, VCAM1
quantity of lymphocytes	1.61E-12	Increased	2.703	BID, CASP1, CASP8, CCL2, CCL4, CCR5, CD274, CD38, CD5, DHX58, EIF2AK2, HAVCR2, HCAR2, HLA-B, HSH2D, IDO1, IFNB1, IL10, IL15, IL15RA, IL1B, IL27, IL7R, IRF1, IRF2, IRF4, JAK2, LGALS9, MCL1, MX1, NAMPT, NFKBIA, PARP14, PIK3CD, PIK3R1, PIM1, PMAIP1, PPBP, PRDM1, PYCARD, RHOH, RNASEL, SEMA4D, SOCS1, STAT1, TLR3, TNF, TNFAIP3, TNFSF10, TNFSF13B
quantity of mononuclear leukocytes	5.16E-12	Increased	3.212	ARNTL, B3GNT5, BID, CASP8, CCL2, CCR2, CCR5, CD274, CD38, CD5, CXCL10, CYP27B1, DAPP1, DDX58, ELF1, ENTPD7, HLA-B, IFNB1, IL10, IL15, IL15RA, IL27, IL7R, IRF1, IRF2, IRF4, ISG15, JAK2, LGALS9, MAP3K8, MCL1, NBN, NFKBIA, PIK3CD, PIK3R1, PIM1, PIM3, PPM1D, PRDM1, PTPN13, PYCARD, RFX5, RHOH, SEMA4D, SOCS1, SOX4, STAT1, TCF4, TEC, TIMD4, TLR3, TNF, TNFAIP3, TNFRSF10A, TNFSF13B, VCAM1
infection of leukocytes	5.16E-12	Increased	3.212	ARNTL, B3GNT5, BID, CASP8, CCL2, CCR2, CCR5, CD274, CD38, CD5, CXCL10, CYP27B1, DAPP1, DDX58, ELF1, ENTPD7, HLA-B, IFNB1, IL10, IL15, IL15RA, IL27, IL7R, IRF1, IRF2, IRF4, ISG15, JAK2, LGALS9, MAP3K8, MCL1, NBN, NFKBIA, PIK3CD, PIK3R1, PIM1, PIM3, PPM1D, PRDM1, PTPN13, PYCARD, RFX5, RHOH, SEMA4D, SOCS1, SOX4, STAT1, TCF4, TEC, TIMD4, TLR3, TNF, TNFAIP3, TNFRSF10A, TNFSF13B, VCAM1
proliferation of B lymphocytes	7.80E-12	Increased	3.218	ARNTL, B3GNT5, BID, CASP8, CCL2, CCR2, CCR5, CD274, CD38, CD5, CXCL10, CYP27B1, DAPP1, DDX58, ELF1, ENTPD7, HLA-B, IFNB1, IL10, IL15, IL15RA, IL27, IL7R, IRF1, IRF2, IRF4, ISG15, JAK2, LGALS9, MAP3K8, MCL1, NBN, NFKBIA, PIK3CD, PIK3R1, PIM1, PIM3, PML, PPM1D, PRDM1, PTPN13, PYCARD, RFX5, RHOH, SEMA4D, SOCS1, SOX4, STAT1, TCF4, TEC, TIMD4, TLR3, TNF, TNFAIP3, TNFRSF10A, TNFSF13B, VCAM1
quantity of T lymphocytes	7.80E-12	Increased	3.218	ARNTL, B3GNT5, BID, CASP8, CCL2, CCR2, CCR5, CD274, CD38, CD5, CXCL10, CYP27B1, DAPP1, DDX58, ELF1, ENTPD7, HLA-B, IFNB1, IL10, IL15, IL15RA, IL27, IL7R, IRF1, IRF2, IRF4, ISG15, JAK2, LGALS9, MAP3K8, MCL1, NBN, NFKBIA, PIK3CD, PIK3R1, PIM1, PIM3, PML, PPM1D, PRDM1, PTPN13, PYCARD, RFX5, RHOH, SEMA4D, SOCS1, SOX4, STAT1, TCF4, TEC, TIMD4, TLR3, TNF, TNFAIP3, TNFRSF10A, TNFSF13B, VCAM1
apoptosis of phagocytes	1.47E-10	Decreased	−2.201	CASP1, CCL4, CCR5, EIF2AK2, HAVCR2, IL15, IRF1, LGALS9, MGLL, PIK3R1, SAMHD1, STAT1, TNF
Cellular homeostasis	1.47E-10	Decreased	−2.201	CASP1, CCL4, CCR5, EIF2AK2, HAVCR2, IL15, IRF1, LGALS9, MGLL, PIK3R1, SAMHD1, STAT1, TNF
**B. Lena ** ***vs.*** ** mock**				
cell death of immune cells	4.78E-08	Increased	2.276	CCL4, CCR5, CD274, DHX58, EIF2AK2, HAVCR2, HCAR2, IFNB1, IL15RA, IL27, IL7R, IL8, MX1, PARP14, PIK3CD, PPBP, RNASEL, SOCS1, TNF, TNFSF10
Cell death	8.23E-08	Increased	2.575	CCL2, CCL4, CCR5, CD274, DHX58, EIF2AK2, HAVCR2, HCAR2, IFNB1, IL15RA, IL27, IL7R, IL8, MX1, PARP14, PIK3CD, PPBP, RNASEL, SOCS1, TNF, TNFSF10
Cell death of lymphocytes	6.11E-07	Increased	2.921	CCL2, CCL4, CCL8, CCR5, CCRL2, CXCL10, EDN1, IFNB1, IL8, PDE4B, PIK3CD, PPBP, TNF
apoptosis of lymphocytes	6.11E-07	Increased	2.921	CCL2, CCL4, CCL8, CCR5, CCRL2, CXCL10, EDN1, IFNB1, IL8, PDE4B, PIK3CD, PPBP, TNF
apoptosis of T lymphocytes	6.11E-07	Increased	2.921	CCL2, CCL4, CCL8, CCR5, CCRL2, CXCL10, EDN1, IFNB1, IL8, PDE4B, PIK3CD, PPBP, TNF
invasion of phagocytes	6.11E-07	Increased	2.921	CCL2, CCL4, CCL8, CCR5, CCRL2, CXCL10, EDN1, IFNB1, IL8, PDE4B, PIK3CD, PPBP, TNF
chemotaxis of antigen presenting cells	2.43E-06	Increased	2.987	ADAR, CCL2, CD274, DDX58, DUSP5, EIF2AK2, IFNB1, IL15RA, IL27, IL7R, IL8, IRF7, JAG1, PIK3CD, PML, RSAD2, SEMA4A, SOCS1, TNF
hematopoiesis of myeloid progenitor cells	2.43E-06	Increased	2.987	ADAR, CCL2, CD274, DDX58, DUSP5, EIF2AK2, IFNB1, IL15RA, IL27, IL7R, IL8, IRF7, JAG1, PIK3CD, PML, RSAD2, SEMA4A, SOCS1, TNF
cell movement of PBMCs	2.43E-06	Increased	2.987	ADAR, CCL2, CD274, DDX58, DUSP5, EIF2AK2, IFNB1, IL15RA, IL27, IL7R, IL8, IRF7, JAG1, PIK3CD, PML, RSAD2, SEMA4A, SOCS1, TNF
inflammatory response	2.53E-06	Increased	2.635	CCL2, CCL4, CCL8, CCR5, CXCL10, IFNB1, IL8, PIK3CD

*probability that a biological function is significantly involved in the analysis.

£ activation state of the biological function; three cases are possible: activation, inhibition or “none”, if the statistical significance is not sufficient for a clear conclusion regarding the activation/inhibition.

& prediction of the activation state of the biological function; IPA uses z-scores to evaluate the activation/inhibition of the biological function. A negative score is indicative of inhibition, while a positive score is indicative of activation. If the statistical significance is not sufficient for a clear conclusion regarding activation/inhibition, we discuss the tendency toward activation/inhibition on the basis of the positive/negative activation z-score.

§ genes involved in the corresponding biological function.

#### 1.2. Canonical pathways and biological functions differentially expressed in the Lena- vs. mock-infected samples

Three of the top five canonical pathways modulated by the infection of PAMs with Lena were shared with those affected by LV infection: “Activation of IRF by Cytosolic Pattern Recognition Receptors”, “Interferon Signaling” and the “Role of Pattern Recognition Receptors in Recognition of Bacteria and Viruses” (Table 1B). The two canonical pathways specific to Lena infection were the “Role of RIG1-like Receptors in Antiviral Innate Immunity”, which is involved in the recognition of viral RNA [38]–[40], and the “Role of Hypercytokinemia/hyperchemokinemia in the Pathogenesis of Influenza”.

Among the top ten biological functions, the “cell death of immune cells and lymphocytes and apoptosis of lymphocytes and T lymphocytes” was up-regulated (Table 2B). Other up-regulated functions related to virus infection included the invasion of phagocytes, chemotaxis of antigen-presenting cells and the inflammatory response. Cell death/survival pathways were less affected by Lena infection than by LV infection and involved different sets of genes (Figure S1-A and B).

#### 1.3. Canonical pathways and biological functions differentially expressed in the LV-and Lena-infected samples

A direct comparison between the LV and Lena infections was performed at the gene, isoform and promoter levels (Table S2). The top five canonical pathways (not taking into account the canonical pathways already found to be differentially expressed in the LV *vs.* mock and Lena *vs.* mock) were “IL-15 production”, “TREM1 signaling”, “Communication between innate and adaptive Immune Cells”, “Crosstalk between dendritic cells and natural killer cells” and the “Role of JAK2 in hormone-like cytokine signaling” (Table 1C). Lena infection triggered the up-regulation of *RPL31* (FC = 243.87), *IL-8* (FC = 144), *PA2G4* (FC = 75) and *CXCL2* (FC = 9.18), while other genes were up-regulated following LV infection, including *P2RY2* (FC = 29.24), *CCL2* (FC = 13.18), *WNT4* (FC = 11.31), *ACADVL* (FC = 7.26), *CXCL10*(FC = 3.97) and *IFNβ1* (FC = 3.48).

Interestingly, *RPL31* and *IL8* were almost exclusively expressed after Lena infection (Figure S2-A and B), while other genes, including *CCL2* and *ACADVL*, were differentially regulated by infection with both strains, although to different extents (Figure S2-C and D). *CXCL10* exhibited three isoforms, but only one isoform (TCONS_00046382) was differentially expressed (Figure S3-A) between the two strains. This gene was also regulated at the TSS level; we identified 3 TSSs, of which one (TSS31082) was differentially regulated between the LV and Lena infections (Figure S3-B).

### 2. Transcriptional and post-transcriptional regulation and differential promoter usage in PAMs after infection with the LV and Lena PRRSV strains

Differentially expressed isoforms with different TSSs are transcriptionally regulated, while differentially expressed isoforms with the same TSSs are regulated at the post-transcriptional level [28]. For the genes involved in the top canonical pathways identified in the previous analyses (LV *vs.* mock, Lena *vs.* mock and LV *vs.* Lena), the regulation of PRRSV infection was investigated at the levels of the transcripts, isoforms and TSSs (Figure S4-B). Three groups of genes were defined:

- Genes with one isoform and one TSS; these genes were classified as “un-spliced and transcriptionally regulated” genes and included *TNF*, *BID*, *CASP8*, *ZC3HAV1*, *IL8*, *CCR5*, *CCL4*, *CCL2* and *TLR4* (Figure S5-Group 1).
- Genes with more than one isoform and one TSS;these genes were classified as “Spliced and post-transcriptionally regulated” genes and included *IFNβ1*, *STAT1*, *TIPARP*, *MICB*, *IRF7*, *CASP1*, *IL1B*, *OAS2* and *STAT2* (Figure S5-Group 2).
- Genes with more than one isoform and more than one TSS. In this group, some isoforms of a given gene are transcriptionally regulated, while others are post-transcriptionally regulated. This group was classified as “Spliced and both transcriptionally and post-transcriptionally regulated”. This group could be further differentiated into two sub-groups (Figure S5-Group 3): (a) “Spliced genes whose isoforms are all transcriptionally regulated”, which included *IFIT3*, *IFIT1*, *IRF1*, *ZBP1*, *ISG15*, *OAS1*, *IFITM1*, *TLR7*, *TNFSF10*, *TNFRSF10A* and *CXCL10*; and (b) “Spliced genes whose isoforms are either transcriptionally or post-transcriptionally regulated”, which included *IL10*, *NFKBIA*, *EIF2AK2*, *PARP14*, *PSMB8*, *DHX58*, *ADAR* and *RNASEL*.

The analysis of differential promoter usage offers an additional perspective to the overview of gene regulation triggered by PRRSV infection. Therefore, we considered the different isoforms that showed the same TSS yields and presented transcripts produced from different start sites [Figure S4-B]. This analysis suggested that 14, 6 and 7 genes were differentially regulated at the promoter level in the LV *vs.* mock, Lena *vs.* mock and LV *vs.* Lena comparisons, respectively (Table S2). Two genes (*PSMB8* and *TIPARP7*) expressed two isoforms, of which one was exclusively expressed in one PRRSV strains (Lena or LV) but not in the other (Figure S5-Group 3 and 2). Moreover, many genes exhibited different levels of expression of the various isoforms under infection with one PRRSV strain *vs.* the other (*PARP14*, *SOCS1*, *IFIT1*, *MX1*, *IFOTM1*, *RNASEL*, *PIKSCD* and *TLR3*; Figure S5).

## Discussion

This study provides insights into the transcriptome of PAMs infected with the LV and Lena PRRSV strains.

### Canonical pathways and biological functions differentially expressed in the LV vs. mock, Lena vs. mock and LV vs. Lena analyses

The biological annotation of the immune response-related genes that were differentially expressed in the PAMs during LV and Lena infection can be interpreted using two sequential steps.

i) The role of the *TLRs* in mounting an immune response during PRRSV infection has been confirmed [41], [42]. Once activated, *IRF1*, *IRF2* and *IRF7* translocate into the nucleus and induce the transcription of *IFN-β* and *IFN-αω*, but not *IFN-α*. *IRF1*, *IRF2* and *IRF7* were all affected by LV infection, but only *IRF7* was found to be modulated after Lena infection. The fact that the PRRSV strains provoked the expression of *IFN-β*, but not *IFN-α*, is in agreement with previous experiments performed in PAMs and dendritic cells *in vitro* [43]–[45].

(ii) Once secreted, IFN-β and most likely IFN-αω are exported to the extracellular space, where they bind to the IFN receptors, provoking the recruitment of *STAT1/STAT2* and inducing the subsequent IFN-stimulated genes: *IFIT1*, *IFIT3*, *IFITM1*, *ISG12*, *ISG15*, *ISG20*, *PKR*, *IRF1*, *JAK2*, *MX1*, *OAS1*, *PSMB8*, *RNaseL* and *SOCS1*;all of these genes play a fundamental role in the host immune response. Specifically, *PKR* dimerizes and phosphorylates *EIF2α* to inhibit the translational process [45], [46]. *OAS*1 is an IFN inducer that is capable of inhibiting protein synthesis and viral growth by degrading viral and cellular RNA [47]. *ISG12*, *ISG15* and *ISG20* are ubiquitin homologs involved in the regulation of innate immunity [48]. *MX1* exhibits antiviral activity [49].

The biological functions altered by Lena and LV infections were focused on cell death/survival, which is considered an innate defense mechanism. We observed considerable interplay between cell death and cell survival, which might explain the discrepancies reported in previous PRRS studies concerning apoptosis [50]–[52].

### Comparison between Lena and LV infections

In this study, the replication rate was slightly higher in LV than Lena strain. Similar results were obtained by Morgan et al. [11] who reported no obvious differences in PRRSV replication between the LV and the “SU1-bel, subtype 3” strain *in vitro*. This differs from results of *in vivo* studies, in which a higher replication rate was reported for Lena compared to other PRRSV subtype 1 strains [6]. Frydas et al [53] have shown that, while LV can replicate in a restricted subpopulation of monocytic cells, Lena appears to target an ample range of cell subpopulations to disseminate within the mucosa. This finding might explain the high replication rate of the Lena strain in vivo, as airway mucosal surface is a common entry site for the virus [53].

### i. Canonical pathways

Two canonical pathways might explain the differences in cell death/survival that were reported for the LV and Lena PRRSV strains: “*IL-15* production” and “Role of JAK2 in Hormone-like Cytokine Signaling”.“*IL-15* production” involved five genes (*IFNβ1*, *IL15*, *IRF1*, *JAK2* and *STAT1*), all of which were up-regulated following LV infection compared to Lena infection. This pathway is one hallmark of the survival and differentiation of NK and T cells [54]. Furthermore, the “Role of JAK2 in Hormone-like Cytokine Signaling” is involved in cell survival and differentiation [55].

The “TREM1 signaling” pathway involved seven genes (*CCL2*, *IL8*, *JAK2*, *TLR3*, *TLR4*, *TLR7* and *TNF*), among which *IL-8* and *TLR4* were up-regulated during Lena infection. It has been reported that TREM1 activation is associated with increases in *TLR4* [56] and interleukin-8 (*IL-8*) expression [57]. Interestingly, *IL-8* was exclusively expressed during Lena and not LV infection, while *TLR4* expression in the LV group was close to null. This finding suggests that TREM1 signaling is mostly specific to Lena infection. Based on a meta-analysis, Badaoui et al. [58] reported the possible involvement of TREM1 signaling during PRRSV infection. This finding could partially explain the high inflammatory response reported following Lena infection (Table S2), as TREM1 is directly implicated in the enhancement of inflammation [59]. From a comparative analysis of PRRSV type 1 and PRRSV type 2, Lee & Lee [60] reported that the up-regulation of IL-8 only occurred 12 h after infection with PRRSV type 2 and was not observed following infection with PRRSV type 1. As IL-8 is known to activate lymphocytes, basophils and neutrophils, its modulation might play an important role in controlling the host immune response.

The interplay between the innate and adaptive immune response is a fundamental aspect of host-pathogen interactions [61]. In this context, two related canonical pathways were found to be differentially expressed in LV- and Lena-infected samples: “Communication between Innate and Adaptive Immune Cells” and “Crosstalk between Dendritic Cells and Natural Killer Cells”. Most of the genes involved in these two canonical pathways were up-regulated to a greater extent by LV infection than by Lena infection. This result suggests that a partial down-regulation of the crosstalk between the innate and adaptive immune responses is triggered by Lena infection. The connection between NK cells and the DC pathway is required for optimal immune cell expansion and activation [62], which might explain the relative decrease in the immune response observed in the Lena-infected samples compared to the LV-infected cells.

### ii. Biological functions

The cell death/survival biological functions were strongly activated during LV infection compared to Lena infection (Figure S1). Moreover, screening the network interactions among the genes involved in cell death/survival for both the LV and Lena strains (Figure S1) showed that the canonical apoptosis pathway signals (*BID*, *CASP8*, *MCL1* and *NFKB)* were activated upon LV infection, but not following Lena infection.

Weesendorp et al. [6] observed a higher apoptosis rate following Lena infection than following LV infection *in vivo*. In other studies, PRRSV was found to induce the apoptosis of lymphocytes and macrophages; this would lead to a decreased host immune response and allow the virus to persist in the host [63], [64]. In addition to the difficulty in directly comparing *in vitro* and *in vivo* approaches, these discrepancies could be explained by ability of Lena to exploit novel cell receptors to target a wider population of cells compared to LV, leading to higher viral replication and higher apoptosis rate *in vivo*.

### iii. Individual genes

*TLR7* and *TLR3* were both activated upon LV infection, but not upon Lena infection (Figure S6). The lack of induction of *TLR3* and/or *TLR7* might be a strategy developed by Lena to escape the host immune response. Su et al. [65] showed that inhibiting the *TLR3/TLR7*signaling pathway hinders an adequate host response to PRRSV infection. However, Zhang et al. [66] found that *TLR3* and *TLR7* were more highly expressed in cells infected with a highly pathogenic PRRSV isolate compared to a weakly pathogenic isolate. This discrepancy from our results might be due to the differences in the immune responses triggered by European PRRSV genotypes 1 and 3, which were used in this study, compared to PRRSV genotype 2, which was employed in [66]. Furthermore, although *TLR4* was expressed in small quantities (average FPKM = 0.4 in Lena), it was shown to be more highly expressed in the Lena group than in the LV group (FC = 5.98) and was expressed at an even lower level in the LV-infected cells compared to mock-infected cells (Figure S6). *TLR4* has been shown to intervene in viral infection with respiratory syncytial virus (RSV) and mouse mammary tumor virus [67].

*IFN-β* and *IFN-αω* were up-regulated following infection with either LV or Lena, but *IFN-β* was more highly expressed in the PAMs infected with LV (Figure 3). The difference in IFN-β expression levels between LV and Lena had thus no apparent impact on their replication rate *in vitro*, which was even slightly lower for Lena. This would point to the previously proposed mechanisms of impairment of the signaling pathway of IFN- β as a main strategy used by PRRSV to block or delay apoptosis until sufficient progeny have been produced [44], [68]. Such impairment could act via individual signaling pathways like RIG-I and Toll like receptor or via a cross talk among several signaling pathways. In addition, some structural proteins of the virus could explain the delayed expression of INF- β [68].

It is worth mentioning that the involvement of *IFN-αω* in macrophage infection by PRRSV is reported here for the first time, likely because this gene was only recently annotated in the context of the structural and functional annotation of the porcine immunome [69].

*IL-10* was observed in the LV group, but not in the Lena group (Table S2). Furthermore, while *TNF-α* was up-regulated in both the LV and Lena groups, it was significantly up-regulated in the LV group compared to the Lena group (Table S2). The induction of *TNF-α* and *IL-10* is used as a criterion for the classification of PRRSV isolates [70]. The relative inhibition of *TNF-α* and *IL-10* might be another mechanism employed by the Lena strain to increase its virulence, as suggested for other PRRSV strains [71], [72].

In an *in vivo* transcriptomic analysis of PAMs [73], of the potential antiviral functions reported to be significant, at least two of the biological functions were also identified in this study: the “up-regulating IFN-induced genes” and “increasing intracellular zinc ion concentration” categories. The intersection between the genes found to be differentially expressed *in vivo* [73] and those that were differentially expressed in this study between the mock and the LV/Lena groups highlighted 22 genes (*BRCA1*, *CCL2*, *CCR5*, *DTX3L*, *GBP1*, *GBP2*, *HERC6*, *IFIH1*, *IFIT1*, *IFIT2*, *IFIT3*, *IRF7*, *ISG15*, *MX1*, *PAPD5*, *PARP9*, *PLAC8*, *PPBP*, *RSAD2*, *SOCS1*, *USP18* and*XAF1*). These genes form a highly significant network centered on IFN signaling (Figure S7). This group of genes could therefore be considered a hallmark of infection by type 1 and type 3 European strains, independent of the infection type (*in vivo*/*in vitro*) and strain virulence (LV/Lena).

### Transcriptional and post-transcriptional regulation and differential promoter use upon infection of the PAMs with the LV and Lena PRRSV strains

Most of the genes that were differentially expressed in the PAMs following PRRSV infection were expressed as several isoforms that were subjected to transcriptional/post-transcriptional regulation and/or differential promoter usage. In the results section, we classified these genes into three main groups (genes with one isoform and one TSS, Figure S5-Group 1; genes with more than one isoform and one TSS, Figure S5-Group 2; and genes with more than one isoform and more than one TSS, Figure S5-Group 3).

The first two groups included genes crucial for the innate immune response, which may be under stronger selection to prevent the emergence of new isoforms and/or post-transcriptional regulation. However, most genes (19) belonged to the third group, suggesting that they could be subjected to positive selection at the transcriptional and post-transcriptional regulation levels.

The analysis of alternative promoter usage highlighted the complexity of transcriptional regulation during the infection of PAMs with PRRSV. *SLC22A15*, a regulator of transmembrane transport, was the only differentially expressed gene that appeared to be significantly regulated at the promoter level, and this gene was identified only in the LV *vs.* mock analysis. However, seven other genes (*MPDZ*, *ANAPC13*, *GPAT*, *ENSSSCG00000029349*, *LIN54*, *ENSSSCG00000025855* and *UBXN11*) were differentially regulated at the promoter level in the LV *vs.* Lena comparison. *MPDZ* is directly involved in host-pathogen interactions [74], whereas *ANAPC13* and *LIN54* are involved in cell cycle progression [75], [76].*UBXN1* is involved in cytoskeleton remodeling [77], and GPAT plays a role in lipid synthesis [78].

## Conclusion

In this study, we used RNA-Seq to profile PAMs following infection with two different strains of PRRSV to obtain a broad overview of the response of PAMs to PRRSV infection. We characterized and compared the cell responses to the two strains in terms of canonical pathways and biological functions, as well as in terms of individual genes. Therefore, our results increase our knowledge of PRRSV infection by consolidating and enriching the previous information generated using microarray technologies.

To the best of our knowledge, this is the first study in which genes, isoforms, TSSs and differential promoter usage were estimated based on RNA-Seq data obtained from PAMs infected with PRRSV. However, further targeted experiments are needed to validate and clarify these types of regulation and to determine their potential effects during macrophage infection.

## Supporting Information

Figure S1 Top network formed by genes that were differentially expressed in (A) LV *vs.* mock and (B) Lena *vs.* mock comparisons that are directly involved in cell death/survival. These two biological functions are highly interconnected and are highlighted in the networks as “cell death” and “cell survival”. In the LV *vs.* mock set, most genes were up-regulated, except for*CD5*, *PYCARD* and *ARG1*, which were down-regulated. In the Lena *vs.* mock set, except for *HSPB1*, all of the genes were up-regulated. The networks were constructed by using focus molecules as “seeds” that were connected together to form a network including the genes in the list. If needed, other non-focus molecules from the dataset were then added to complete the network. The resulting networks were scored and then sorted based on the score. The network scores represent the negative log of the p-value of the likelihood that the network molecules were found together by chance. Therefore, a high score represents a san index indicating that the interconnection of the molecules within the network is more likely to be true.
(PDF)

Figure S2 Example of top genes that were differentially expressed between the LV and Lena groups (among the largest fold changes). (I) Expressed only in the Lena and not in the LV group: (A) *RPL31* and (B) *IL8*. (II) Expressed in high quantities in both the LV and Lena groups: (C) *CCL2*. (III) Expressed in the LV group, but in a very low quantity in the Lena group: (D)*ACADVL*.
(TIFF)

Figure S3 Example of the top (among the largest fold changes) differentially expressed genes (*CXCL10*) that were regulated transcriptionally and post-transcriptionally. *CXCL10*was presentin three isoforms, among which “TCONS_00046382” was differentially expressed between the LV and Lena groups. *CXCL10* was also regulated at the TSS level, as it displayed three TSSs, among which “TSS31082” was differentially regulated between the LV and Lena groups.
(TIFF)

Figure S4 Cufflinks approaches for estimating (A) transcripts and their abundances and (B) transcriptional and post-transcriptional regulatory effects on overall transcript output. In Figure S4-A: For the whole analysis, Cufflinks used the paired-end reads that were aligned to the reference genome (Build Sus_scrofa.Sscrofa10.2.71.),using the TopHat software, to perform the spliced alignments (a). Cufflinks starts by connecting the compatible fragments in an overlap graph. Thus, Cufflinks applies Dilworth's Theorem, which yields a minimal set of paths that cover all of the fragments in the overlap graph, by finding the largest set of reads meeting the criterion that no two reads could have originated from the same isoform (b,c). Subsequently, Cufflinks estimates transcript abundance using a statistical model in which the probability of observing each fragment is a linear function of the abundances of the transcripts from which it could have originated (d). The last step consists of maximizing the likelihood function for all possible sets of relative transcript abundance to determine the set that best explains the observed fragments (e). In Figure S4-B: (a) When the abundance of isoforms A, B and C are grouped by TSS, the changes in the relative abundance of the TSS groups indicate transcriptional regulation (A+B *vs.* C). Post-transcriptional effects are observed as changes in the levels of the isoforms ina single TSS group (A *vs.* B) (Adapted from Trapnell et al. 2012).
(PDF)

Figure S5 Transcriptional/post-transcriptional regulation of the genes involved in the top canonical pathways in the LV *vs.* mock, Lena *vs.* mock and LV *vs.* Lena comparisons. (A) Un-spliced and transcriptionally regulated genes,(B) spliced and post-transcriptionally regulated genes and(C) spliced and both transcriptionally and post-transcriptionally regulated genes. For each transcript,the “XLOC”, “TSS” and “TCONS” suffixes correspond to the genes, TSSs and isoforms, respectively. Differentially expressed isoforms with different TSSs are transcriptionally regulated, while isoforms with the same TSS are regulated at the post-transcriptional level (Figure S4).
(PDF)

Figure S6 Differential expression of*TLR3*, *TLR4* and *TLR7* between the LV and Lena groups. *TLR7* and *TLR3* were significantly up-regulated in the LV group, while neither *TLR7* nor *TLR3*wasmodulated in the Lena group. Although *TLR4* was expressed in small quantities (medium FPKM = 0.4 in Lena), it was more highly expressed in the Lena group than in the LV group (FC = 5.98) and was expressed at an even lower level during LV infection than during mock infection.
(PDF)

Figure S7 Network formed by common genes that were differentially expressed in the LV *vs.* mock and Lena *vs.* mock comparisons from one side and in an *in vivo* study performed by Zhou et al. (2001) from the other side. The canonical pathways that were significantly affected by this group of genes are highlighted with blue squares and include interferon signaling, the activation of IRF by cytosolic pattern recognition receptors, the role of hypercytokinemia/hyperchemokinemia in the pathogenesis of influenza, the role of RIG1-like receptors in antiviral innate immunity and the role of PI3K/AKT signaling in the pathogenesis of influenza. The networks were constructed using focus molecules as “seeds” that were connected together to form a network using the genes in the list. If needed, other non-focus molecules from the dataset were then added to complete the network. The resulting networks were scored and then sorted based on the score. The network scores represent the negative log of the p-value of the likelihood that the network molecules were found together by chance. Therefore, a high score represents an index indicating that the interconnection of the molecules within the network is more likely to be true.
(PDF)

Table S1 Summary of the reads generated per sample (mock, LV and Lena). A. Numbers of RNA-Seq reads generated per sample (mock, LV and Lena). B. RNA-Seq reads eliminated following quality evaluation via FASTQC and Sickle. C. RNA-Seq reads mapped to the pig genome (Build Sus_scrofa.Sscrofa10.2.71.) using the TopHat v2.0.8 algorithm.
(DOC)

Table S2 Genes, isoforms, TSSs and promoter usage showing differential expression in the LV *vs.* mock, Lena *vs.* mock and LV *vs.* Lena comparisons. Genes_Mock_*vs*_LV: differentially expressed genes between the LV and mock infections; Genes_Mock_*vs*_Lena: differentially expressed genes between the Lena and mock infections; Genes_LV_*vs*_Lena: differentially expressed genes between the LV and Lena infections. Isoforms_Mock_*vs*_LV: differentially expressed isoforms between the LV and mock infections; Isoforms_Mock_*vs*_Lena: differentially expressed isoforms between the Lena and mock infections; Isoforms_LV_*vs*_Lena: differentially expressed isoforms between the LV and Lena infections. TSS_Mock_*vs*_LV: differentially expressed TSSs between the LV and mock infections; TSS_Mock_*vs*_Lena: differentially expressed TSSs between the Lena and mock infections; TSS_LV_*vs*_Lena: differentially expressed TSSs between the LV and Lena infections. Promoters_Mock_*vs*_LV: differentially used promoters between the LV and mock infections; Promoter_Mock_*vs*_Lena: differentially used promoters between the Lena and mock infections; Promoter_LV_*vs*_Lena: differentially used promoters between the LV and Lena infections. Each page is composed of 7 columns: test_id, gene_id, gene, gene-map, locus, sample_1, sample_2, status, value_1, value_2, log2.fold_change, test_stat, p_value, q_value and significant.
(XLS)

## References

[1] Neumann EJ, Kliebenstein JB, Johnson CD, Mabry JW, Bush EJ, et al (2005) Assessment of the economic impact of porcine reproductive and respiratory syndrome on swine production in the United States. Journal of the American Veterinary Medical Association 227: 385–392. 16121604 10.2460/javma.2005.227.385

[2] Nieuwenhuis N, Duinhof TF, van Nes A (2012) Economic analysis of outbreaks of porcine reproductive and respiratory syndrome virus in nine sow herds. Vet Rec 170 9: 225. 22238201 10.1136/vr.100101

[3] Mardassi L, Wilson S, Mounir and Dea S (1994) Detection of porcine reproductive and respiratory syndrome virus and efficient differentiation between Canadian and European strains by reverse transcription and PCR amplification. J Clin Microbiol 32: 2197–2203. 7814546 10.1128/jcm.32.9.2197-2203.1994 PMC263966

[4] Stadejek T, Oleksiewicz MB, Scherbakov AV, Timina AM, Krabbe JS, et al (2008) Definition of subtypes in the European genotype of porcine reproductive and respiratory syndrome virus: nucleocapsid characteristics and geographical distribution in Europe. Arch Virol 153: 1479–1488. 18592131 10.1007/s00705-008-0146-2

[5] Karniychuk UU, Geldhof M, Vanhee M, Van Doorsselaere J, Saveleva TA, et al (2010) Pathogenesis and antigenic characterization of a new East European subtype 3 porcine reproductive and respiratory syndrome virus isolate. BMC Vet Res 6: 30. 20525333 10.1186/1746-6148-6-30 PMC2898778

[6] Weesendorp E, Morgan S, Stockhofe-Zurwieden N, Popma-De Graaf DJ, Graham SP, et al (2013) Comparative analysis of immune responses following experimental infection of pigs with European porcine reproductive and respiratory syndrome virus strains of differing virulence. Vet Microbiol 163: 1–12. 23036445 10.1016/j.vetmic.2012.09.013 PMC7117209

[7] Lee SM, Schommer SK, Kleiboeker SB (2004) Porcine reproductive and respiratory syndrome virus field isolates differ in *in vitro* interferon phenotypes. Vet Immunol Immunopathol 102: 217–231. 15507307 10.1016/j.vetimm.2004.09.009 PMC7112598

[8] Díaz I, Darwich L, Pappaterra G, Pujols J, Mateu E (2006) Different European-type vaccines against porcine reproductive and respiratory syndrome virus have different immunological properties and confer different protection to pigs. Virology 351: 249–59. 16712895 10.1016/j.virol.2006.03.046

[9] Johnson W, Roof M, Vaughn E, Christopher-Hennings J, Johnson CR, et al (2004) Pathogenic and humoral immune responses to porcine reproductive and respiratory syndrome virus (PRRSV) are related to viral load in acute infection. Vet Immunol Immunopathol 102: 147–233. 10.1016/j.vetimm.2004.09.010 15507308

[10] Loving CL, Brockmeier SL, Vincent AL, Lager KM, Sacco RE (2008) Differences in clinical disease and immune response of pigs challenged with a high-dose versus low-dose inoculum of porcine reproductive and respiratory syndrome virus. Viral Immunol 21: 315–325. 18788940 10.1089/vim.2008.0038

[11] Morgan SB, Graham SP, Salguero FJ, Sánchez Cordón PJ, Mokhtar H, et al (2013) Increased pathogenicity of European porcine reproductive and respiratory syndrome virus is associated with enhanced adaptive responses and viral clearance. Vet Microbiol 163 1–2: 13–22. 23313323 10.1016/j.vetmic.2012.11.024

[12] Kimman TG, Cornelissen LA, Moormann RJ, Rebel JM, Stockhofe-Zurwieden N (2009) Challenges for porcine reproductive and respiratory syndrome virus (PRRSV) vaccinology. Vaccine 27: 3704–3718. 19464553 10.1016/j.vaccine.2009.04.022

[13] Mateu E, Diaz I (2008) The challenge of PRRS immunology. Vet J 177: 345–351. 17644436 10.1016/j.tvjl.2007.05.022 PMC7110845

[14] Badaoui B, Grande R, Calza S, Cecere M, Luini M, et al (2013) Impact of genetic variation and geographic distribution of Porcine 1 Reproductive and Respiratory Syndrome Virus on infectivity and pig growth. BMC Veterinary Research 9: 58. 23537091 10.1186/1746-6148-9-58 PMC3762063

[15] Pitkin A, Deen J, Dee S (2009) Use of a production region model to assess the airborne spread of porcine reproductive and respiratory syndrome virus. Vet Microbiol 136: 1–7. 19046835 10.1016/j.vetmic.2008.10.013

[16] Prieto C, Vázquez A, Núñez JI, Alvarez E, Simarro I, Castro JM (2009) Influence of time on the genetic heterogeneity of Spanish porcine reproductive and respiratory syndrome virus isolates. Vet J 180: 363–370. 18684650 10.1016/j.tvjl.2008.01.005

[17] Reiner G (2009) Investigations on genetic disease resistance in swine: a contribution to the reduction of pain, suffering and damage in farm animals. Appl Anim Behav Sci 118: 217–221.

[18] Zhang X, Shin J, Molitor TW, Schook LB, Rutherford MS (1999) Molecular responses of macrophages to porcine reproductive and respiratory syndrome virus infection. Virology 262: 152–162. 10489349 10.1006/viro.1999.9914

[19] Delputte PL, Nauwynck HJ (2004) Porcine arterivirus infection of alveolar macrophages is mediated by sialic acid on the virus. J Virol 78: 8094–8101. 15254181 10.1128/JVI.78.15.8094-8101.2004 PMC446125

[20] Jiang Z, Zhou X, Michal JJ, Wu X-L, Zhang L, et al (2013) Reactomes of Porcine Alveolar Macrophages Infected with Porcine Reproductive and Respiratory Syndrome Virus. PLoS ONE 8 3: e59229. 23527143 10.1371/journal.pone.0059229 PMC3602036

[21] Flores-Mendoza L, Silva-Campa E, Reséndiz M, Osorio FA, Hernández J (2008) Porcine reproductive and respiratory syndrome virus infects mature porcine dendritic cells and up-regulates interleukin-10 production. Clinical and Vaccine Immunology 15 720–725. 18272667 10.1128/CVI.00224-07 PMC2292656

[22] Murtaugh MP, Xiao Z, Zuckermann F (2002) Immunological responses of swine to porcine reproductive and respiratory syndrome virus infection. Viral Immunology 15 533–547. 12513925 10.1089/088282402320914485

[23] Sun Y, Han M, Kim C, Calvert JG, Yoo D (2012) Interplay between Interferon-Mediated Innate Immunity and Porcine Reproductive and Respiratory Syndrome Virus. Viruses 4: 424–446. 22590680 10.3390/v4040424 PMC3347317

[24] Richard H, Schulz MH, Sultan M, Nurnberger A, Schrinner S, et al (2010) Prediction of alternative isoforms from exon expression levels in RNA-Seq experiments. Nucleic Acids Res 38: e112. 20150413 10.1093/nar/gkq041 PMC2879520

[25] Sultan M, Schulz MH, Richard H, Magen A, Klingenhoff A, et al (2008) A global view of gene activity and alternative splicing by deep sequencing of the human transcriptome. Science 321: 956–960. 18599741 10.1126/science.1160342

[26] Nagalakshmi U, Wang Z, Waern K, Shou C, Raha D, et al (2008) The transcriptional landscape of the yeast genome defined by RNA sequencing. Science 320: 1344–1349. 18451266 10.1126/science.1158441 PMC2951732

[27] Wilhelm BT, Marguerat S, Watt S, Schubert F, Wood V, et al (2008) Dynamic repertoire of a eukaryotic transcriptome surveyed at single-nucleotide resolution. Nature 453: 1239–1243. 18488015 10.1038/nature07002

[28] Mortazavi A, Williams BA, McCue K, Schaeffer L, Wold B (2008) Mapping and quantifying mammalian transcriptomes by RNA-Seq. Nature Methods 5: 621–628. 18516045 10.1038/nmeth.1226 PMC13303166

[29] Davuluri RV, Suzuki Y, Sugano S, Plass C, Huang TH (2008) The functional consequences of alternative promoter use in mammalian genomes. Trends Genet 24: 167–177. 18329129 10.1016/j.tig.2008.01.008

[30] Wensvoort G, Terpstra C, Pol JM, ter Laak EA, Bloemraad M, et al (1991) Mystery swine disease in The Netherlands: the isolation of Lelystad virus. Vet Q 13: 121–130. 1835211 10.1080/01652176.1991.9694296

[31] Delputte PL, Nauwynck HJ (2004) Porcine arterivirus infection of alveolar macrophages is mediated by sialic acid on the virus. J Virol 78: 8094–8101. 15254181 10.1128/JVI.78.15.8094-8101.2004 PMC446125

[32] Langmead B, Trapnell C, Pop M, Salzberg SL (2009) Ultrafast and memory efficient alignment of short DNA sequences to the human genome. Genome Biol 10: R25. 19261174 10.1186/gb-2009-10-3-r25 PMC2690996

[33] Zweig AS, Karolchik D, Kuhn RM, Haussler D, Kent WJ (2008) UCSC genome browser tutorial. Genomics 92: 75–84. 18514479 10.1016/j.ygeno.2008.02.003

[34] Trapnell C, Williams BA, Pertea G, Mortazavi A, Kwan G, et al (2010) Transcript assembly and quantification by RNA-Seq reveals unannotated transcripts and isoform switching during cell differentiation. Nat Biotechnol 28: 511–515. 20436464 10.1038/nbt.1621 PMC3146043

[35] Jiang H, Wong WH (2009) Statistical inferences for isoform expression in RNASeq. Bioinformatics 25: 1026–1032. 19244387 10.1093/bioinformatics/btp113 PMC2666817

[36] Robinson MD, McCarthy DJ, Smyth GK (2010) edgeR: a Bioconductor package for differential expression analysis of digital gene expression data. Bioinformatics 26: 139–140. 19910308 10.1093/bioinformatics/btp616 PMC2796818

[37] Sadler AJ, Williams BR (2008) Interferon-inducible antiviral effectors. Nat Rev Immuno 8: 559–568. 10.1038/nri2314 PMC2522268 18575461

[38] Moretta L, Bottino C, Pende D, Mingari MC, Biassoni R, Moretta A (2002) Human natural killer cells: their origin, receptors and function. Eur J Immunol 32: 1205–1211. 11981807 10.1002/1521-4141(200205)32:5<1205::AID-IMMU1205>3.0.CO;2-Y

[39] Onomoto K, Yoneyama M, Fujita T (2007) Regulation of antiviral innate immune responses by RIG-I family of RNA helicases. Current Topics in Microbiology and Immunology 316: 193–205. 17969449 10.1007/978-3-540-71329-6_10

[40] Luo R, Xiao S, Jiang Y, Jin H, Wang D, et al (2008) Fang L. Porcine reproductive and respiratory syndrome virus (PRRSV) suppresses interferon-beta production by interfering with the RIG-I signaling pathway. Molecular Immunology 45: 2839–2846. 18336912 10.1016/j.molimm.2008.01.028 PMC7112510

[41] Liu CH, Chaung HC, Chang HL, Peng YT, Chung WB (2009) Expression of Toll-like receptor mRNA and cytokines in pigs infected with porcine reproductive and respiratory syndrome virus. Veterinary Microbiology 136: 266–276. 19124206 10.1016/j.vetmic.2008.11.016

[42] Xiao S, Jia J, Mo D, Wang Q, Qin L, et al (2010) Understanding PRRSV Infection in Porcine Lung Based on Genome-Wide Transcriptome Response Identified by Deep Sequencing. PLoS ONE 2010 5 6: e11377. 10.1371/journal.pone.0011377 PMC2894071 20614006

[43] Ait-Ali T, Wilson AD, Westcott DG, Frossard JP, Mellencamp MA, et al (2008) Dynamic differential regulation of innate immune response transcripts during the infection of alveolar macrophages by the porcine reproductive and respiratory syndrome virus. Dev Biol 132: 239–245. 10.1159/000317166 18817308

[44] Genini S, Delputte PL, Malinverni R, Cecere M, Stella A, et al (2008) Genome-wide transcriptional response of primary alveolar macrophages following infection with porcine reproductive and respiratory syndrome virus. J Gen Virol 89: 2550–2564. 18796724 10.1099/vir.0.2008/003244-0 PMC2885007

[45] Loving CL, Brockmeier SL, Sacco RE (2007) Differential type I interferon activation and susceptibility of dendritic cell populations to porcine arterivirus. Immunology 120: 217–229. 17116172 10.1111/j.1365-2567.2006.02493.x PMC2265861

[46] Tamura T, Tailor P, Yamaoka K, Kong HJ, Tsujimura H, et al (2008) IFN regulatory factor-4 and -8 govern dendritic cell subset development and their functional diversity. J Immunol 174: 2573–2581. 10.4049/jimmunol.174.5.2573 15728463

[47] Eskildsen S, Justesen J, Schierup MH, Hartmann R (2003) Characterization of the 2–5-oligodenylate synthetase ubiquitin-like family. Nucleic Acids Res 31: 2166–3173. 10.1093/nar/gkg427 PMC162331 12799444

[48] Blomstrom DC, Fahey D, Kutny R, Korant BD, Knight EJ (1986) Molecular characterization of the interferon-induced 15-kDa protein. Molecular cloning and nucleotide and amino acid sequence. J Biol Chem 261: 8811–8816. 3087979

[49] Haller O, Arnheiter H, Lindenmann J, Gresser I (1980) Host gene influences sensitivity to interferon action selectively for influenza virus. Nature 283: 660–662. 7354853 10.1038/283660a0

[50] Kim TS, Benfield DA, Rowland RR (2002) Porcine reproductive and respiratory syndrome virus-induced cell death exhibits features consistent with a nontypical form of apoptosis. Virus Res 2002 85: 133–140. 10.1016/s0168-1702(02)00029-1 12034480

[51] Miller LC (2004) Fox JM (2004) Apoptosis and porcine reproductive and respiratory syndrome virus. Vet Immunol Immunopathol 102: 131–142. 15507300 10.1016/j.vetimm.2004.09.004

[52] Lee C, Bachand A, Murtaugh MP, Yoo D (2004) Differential host cell gene expression regulated by the porcine reproductive and respiratory syndrome virus GP4 and GP5 glycoproteins. Vet Immunol Immunopathol 102: 189–198. 15507305 10.1016/j.vetimm.2004.09.020 PMC7112691

[53] Frydas IS, Verbeeck M, Cao J, Nauwynck HJ (2013) Replication characteristics of porcine reproductive and respiratory syndrome virus (PRRSV) European subtype 1 (Lelystad) and subtype 3 (Lena) strains in nasal mucosa and cells of the monocytic lineage: indications for the use of new receptors of PRRSV (Lena). Vet Res 4: 44: 73. 10.1186/1297-9716-44-73 PMC3849772 24007551

[54] Ahmad R, Ennaciri J, Cordeiro P, El Bassam S, Menezes J (2007) Herpes simplex virus-1 up-regulates IL-15 gene expression in monocytic cells through the activation of protein tyrosine kinase and PKC zeta/lambda signaling pathways. J Mol Biol 16: 367 1: 25–35. 10.1016/j.jmb.2006.12.060 17239392

[55] Carter-Su C, Rui L, Herrington J (2000) Role of the tyrosine kinase JAK2 in signal transduction by growth hormone. Pediatr Nephrol 14 7: 550–7. 10912517 10.1007/s004670000366

[56] Fortin CF, Lesur O, Fulop T Jr (2007) Effects of TREM-1 activation in human neutrophils: activation of signaling pathways, recruitment into lipid rafts and association with TLR4. Int Immunol 19: 41–50. 17098818 10.1093/intimm/dxl119

[57] Tessarz AS, Weiler S, Zanzinger K, Angelisova P, Horejsi V, et al (2007) Non-T cell activation linker (NTAL) negatively regulates TREM-1/DAP12-induced inflammatory cytokine production in myeloid cells. J Immunol 178: 1991–1999. 17277102 10.4049/jimmunol.178.4.1991

[58] Badaoui B, Tuggle CK, Hu Z-L, Reecy J, Ait-Ali T, et al (2013) Pig immune response to general stimulus and to Porcine Reproductive and Respiratory Syndrome Virus infection: a meta-analysis approach. BMC Genomics 14: 220. 23552196 10.1186/1471-2164-14-220 PMC3623894

[59] Bleharski JR, Li H, Meinken C, Graeber TG, Ochoa MT, et al (2003) Use of genetic profiling in leprosy to discriminate clinical forms of the disease. Science 301: 1527–1530. 12970564 10.1126/science.1087785

[60] Lee YJ, Lee C (2013) Cytokine production in immortalized porcine alveolar macrophages infected with porcine reproductive and respiratory syndrome virus. 150: 213–220. 10.1016/j.vetimm.2012.09.007 23041033

[61] Luo R, Xiao S, Jiang Y, Jin H, Wang D, et al (2008) Porcine reproductive and respiratory syndrome virus (PRRSV) suppresses interferon-beta production by interfering with the RIG-I signaling pathway. Molecular Immunology 45: 2839–2846. 18336912 10.1016/j.molimm.2008.01.028 PMC7112510

[62] Moretta L, Bottino C, Pende D, Mingari MC, Biassoni R, et al (2002) Human natural killer cells: their origin, receptors and function. Eur J Immunol 32: 1205–1211. 11981807 10.1002/1521-4141(200205)32:5<1205::AID-IMMU1205>3.0.CO;2-Y

[63] Labarque G, Van Gucht S, Nauwynck H, Van Reeth K, Pensaert M (2003) Apoptosis in the lungs of pigs infected with porcine reproductive and respiratory syndrome virus and associations with the production of apoptogenic cytokines. Vet Res 34: 249–260. 12791235 10.1051/vetres:2003001

[64] Gómez-Laguna J, Salguero FJ, Fernández de Marco M, Barranco I, Rodríguez-Gómez IM, et al (2012) Type 2 porcine reproductive and respiratory syndrome virus infection mediated apoptosis in B- and T-cell areas in lymphoid organs of experimentally infected pigs. Trans boundary and Emerging Diseases http://dx.doi.org/10.1111/j.1865-1682.2012.01338.x. 10.1111/j.1865-1682.2012.01338.x 22607093

[65] Su X, Li S, Meng M, Qian W, Xie W, et al (2006) TNF receptor-associated factor-1 (TRAF1) negatively regulates Toll/IL-1 receptor domain-containing adaptor inducing IFN-β (TRIF)-mediated signaling. Eur J Immunol 36: 199–206. 16323247 10.1002/eji.200535415

[66] Zhang L, Liu J, Bai J, Wang X, Li Y, et al (2013) Comparative expression of Toll-like receptors and inflammatory cytokines in pigs infected with different virulent porcine reproductive and respiratory syndrome virus isolates. 10: 135–146. 10.1186/1743-422X-10-135 PMC3673858 23631691

[67] Haeberle HA, Takizawa R, Casola A, Brasier AR, Dieterich HJ, et al (2002) Respiratory syncytial virus-induced activation of nuclear factor-κB in the lung involves alveolar macrophages and toll-like receptor 4-dependent pathways. J Infect Dis 186: 1199–1206. 12402188 10.1086/344644

[68] Zhou A, Zhang S (2012) Regulation of cell signaling and porcine reproductive and respiratory syndrome virus. Cell Signal 24 5: 973–80. 22274732 10.1016/j.cellsig.2012.01.004

[69] Dawson HD, Loveland JE, Pascal G, Gilbert JG, Uenishi H, et al (2013) Structural and Functional Annotation of the Porcine Immunome. BMC genomics 14: 332. 23676093 10.1186/1471-2164-14-332 PMC3658956

[70] Gimeno M, Darwich L, Díaz I, de la Torre E, Pujols J, et al (2011) Cytokine profiles and phenotype regulation of antigen presenting cells by genotype-I porcine reproductive and respiratory syndrome virus isolates. Veterinary Research 42: 9. 21314968 10.1186/1297-9716-42-9 PMC3037899

[71] Hou J, Wang L, He W, Zhang H, Feng WH (2012) Highly pathogenic porcine reproductive and respiratory syndrome virus impairs LPS- and poly(I:C)-stimulated tumor necrosis factor-alpha release by inhibiting ERK signaling pathway. Virus Research 167: 106–111. 22497732 10.1016/j.virusres.2012.03.017

[72] Díaz I, Darwich L, Pappaterra G, Pujols J, Mateu E (2006) Different European type vaccines against porcine reproductive and respiratory syndrome virus have different immunological properties and confer different protection to pigs. Virology 351: 249–259. 16712895 10.1016/j.virol.2006.03.046

[73] Zhou P, Zhai S, Zhou X, Lin P, Jiang T, et al (2011) Molecular characterization of transcriptome-wide interactions between highly pathogenic porcine reproductive and respiratory syndrome virus and porcine alveolar macrophages in vivo. Int J BiolSci 7 7: 947–59. 10.7150/ijbs.7.947 PMC3157269 21850204

[74] Konig R, Zhou Y, Elleder D, Diamond TL, Bonamy GM, et al (2008) Global analysis of host-pathogen interactions that regulate early-stage HIV-1 replication. Cell 3:135 1: 49–60. 10.1016/j.cell.2008.07.032 PMC2628946 18854154

[75] Schwickart M, Havlis J, Habermann B, Bogdanova A, Camasses A, et al.. (2004) Swm1/Apc13 is an evolutionarily conserved subunit of the anaphase-promoting complex stabilizing the association of Cdc16 and Cdc27. 10.1128/MCB.24.8.3562-3576.2004 PMC381669 15060174

[76] Schmit F, Cremer S, Gaubatz S (2009) LIN54 is an essential core subunit of the DREAM/LINC complex that binds to the cdc2 promoter in a sequence-specific manner. FEBS J 276: 5703–5716. 19725879 10.1111/j.1742-4658.2009.07261.x

[77] Katoh H, Harada A, Mori K, Negishi M (2002) Socius is a novel Rnd GTPase-interacting protein involved in disassembly of actin stress fibers. Mol Cell Biol 1:22 9: 2952–64. 10.1128/MCB.22.9.2952-2964.2002 PMC133765 11940653

[78] Chen YQ, Kuo MS, Li S, Bui HH, Peake DA, et al (2008) AGPAT6 is a novel microsomal glycerol-3- phosphate acyltransferase. J BiolChem 83 15: 10048–57. 10.1074/jbc.M708151200 PMC2442282 18238778

