# Supplementary figures and images for: RNA-Sequence Analysis of Primary Alveolar Macrophages after In Vitro Infection with Porcine Reproductive and Respiratory Syndrome Virus Strains of Differing Virulence
Source: PLoS One. 2014 Mar 18;9(3):e91918. doi: 10.1371/journal.pone.0091918 (PMC3958415; doi:10.1371/journal.pone.0091918)

A

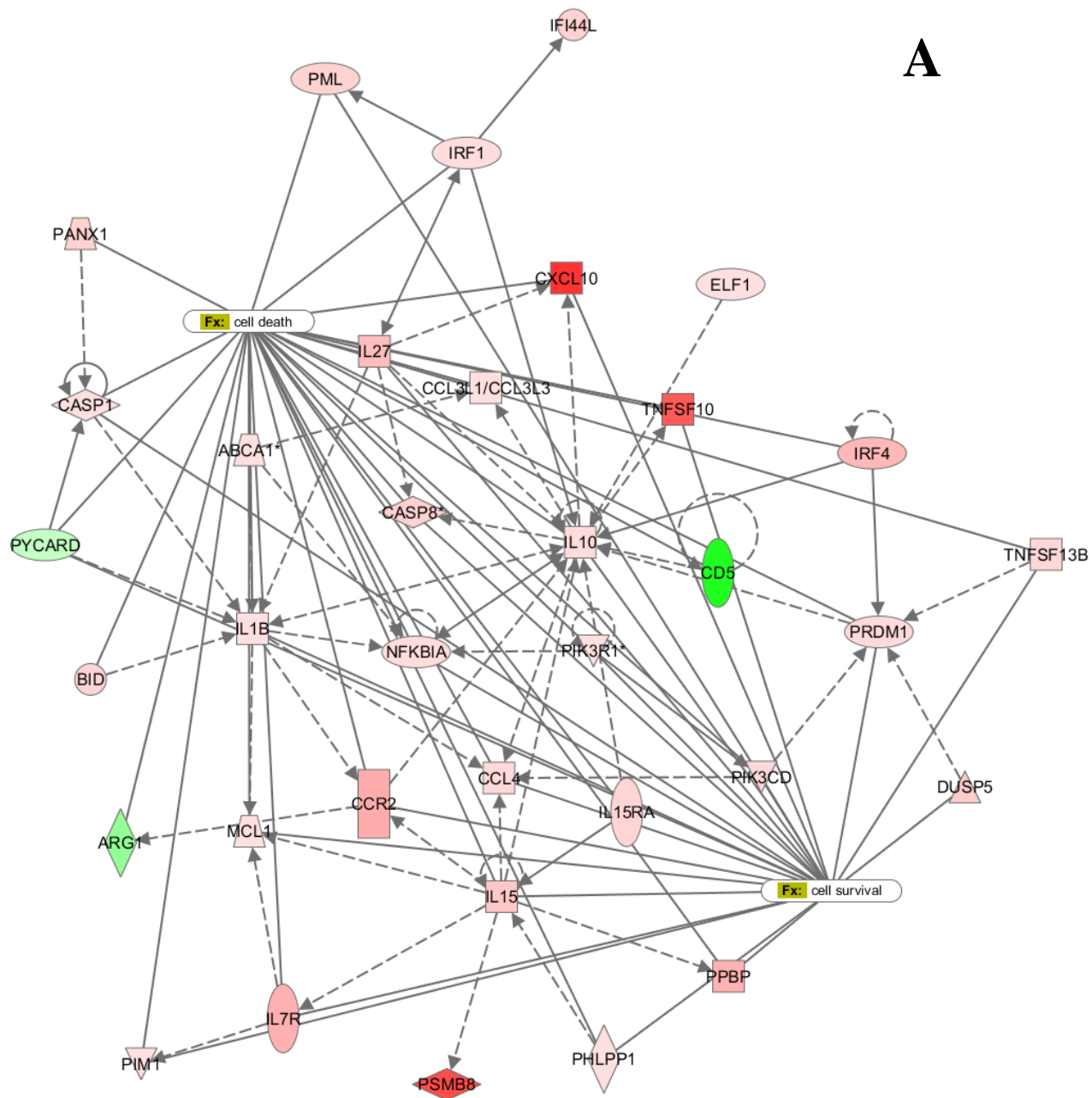

# B

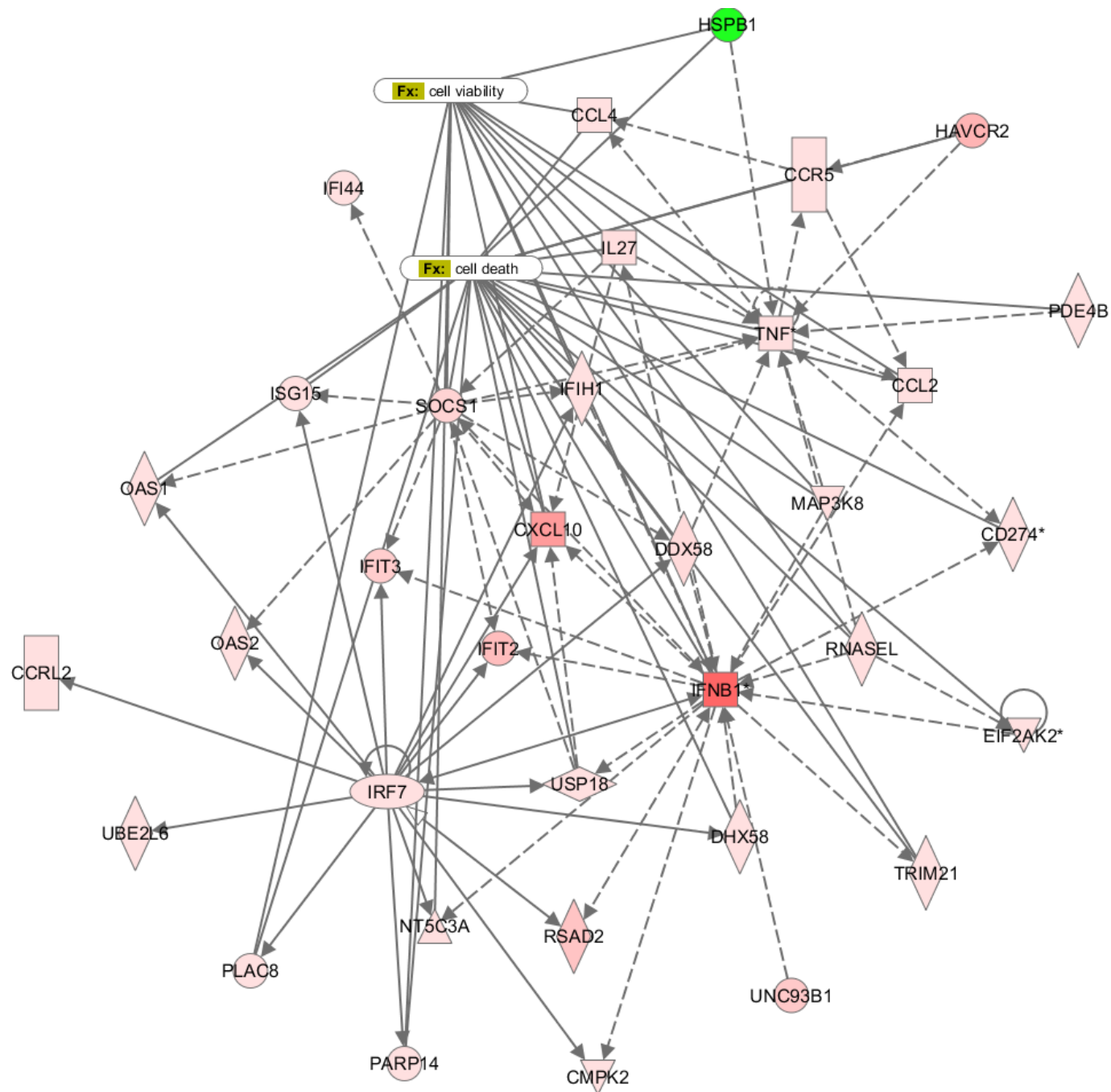

Supplement: Figure S1 — Top network formed by genes that were differentially expressed in (A) LV vs. mock and (B) Lena vs. mock comparisons that are directly involved in cell death/survival. These two biological functions are highly interconnected and are highlighted in the networks as “cell death” and “cell survival”. In the LV vs. mock set, most genes were up-regulated, except forCD5, PYCARD and ARG1, which were down-regulated. In the Lena vs. mock set, except for HSPB1, all of the genes were up-regulated. The networks were constructed by using focus molecules as “seeds” that were connected together to form a network including the genes in the list. If needed, other non-focus molecules from the dataset were then added to complete the network. The resulting networks were scored and then sorted based on the score. The network scores represent the negative log of the p-value of the likelihood that the network molecules were found together by chance. Therefore, a high score represents a san index indicating that the interconnection of the molecules within the network is more likely to be true. (PDF) [file pone.0091918.s001.pdf]

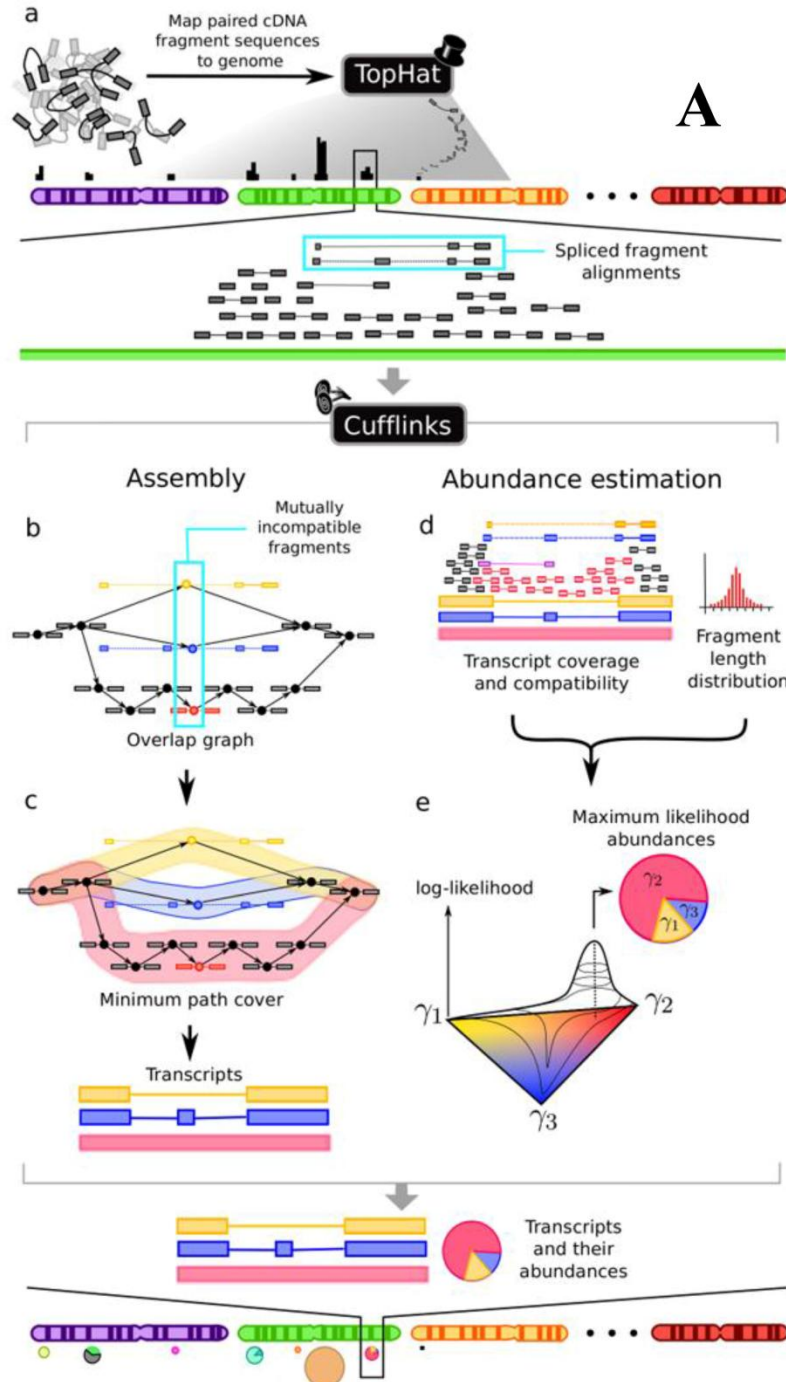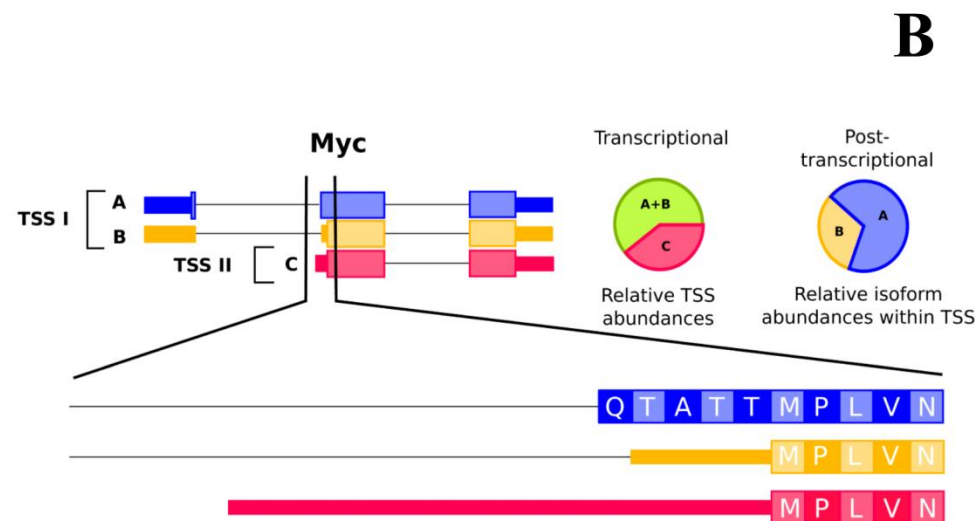

Supplement: Figure S4 — Cufflinks approaches for estimating (A) transcripts and their abundances and (B) transcriptional and post-transcriptional regulatory effects on overall transcript output. In Figure S4-A: For the whole analysis, Cufflinks used the paired-end reads that were aligned to the reference genome (Build Sus_scrofa.Sscrofa10.2.71.),using the TopHat software, to perform the spliced alignments (a). Cufflinks starts by connecting the compatible fragments in an overlap graph. Thus, Cufflinks applies Dilworth's Theorem, which yields a minimal set of paths that cover all of the fragments in the overlap graph, by finding the largest set of reads meeting the criterion that no two reads could have originated from the same isoform (b,c). Subsequently, Cufflinks estimates transcript abundance using a statistical model in which the probability of observing each fragment is a linear function of the abundances of the transcripts from which it could have originated (d). The last step consists of maximizing the likelihood function for all possible sets of relative transcript abundance to determine the set that best explains the observed fragments (e). In Figure S4-B: (a) When the abundance of isoforms A, B and C are grouped by TSS, the changes in the relative abundance of the TSS groups indicate transcriptional regulation (A+B vs. C). Post-transcriptional effects are observed as changes in the levels of the isoforms ina single TSS group (A vs. B) (Adapted from Trapnell et al. 2012). (PDF) [file pone.0091918.s004.pdf]

# TLR3

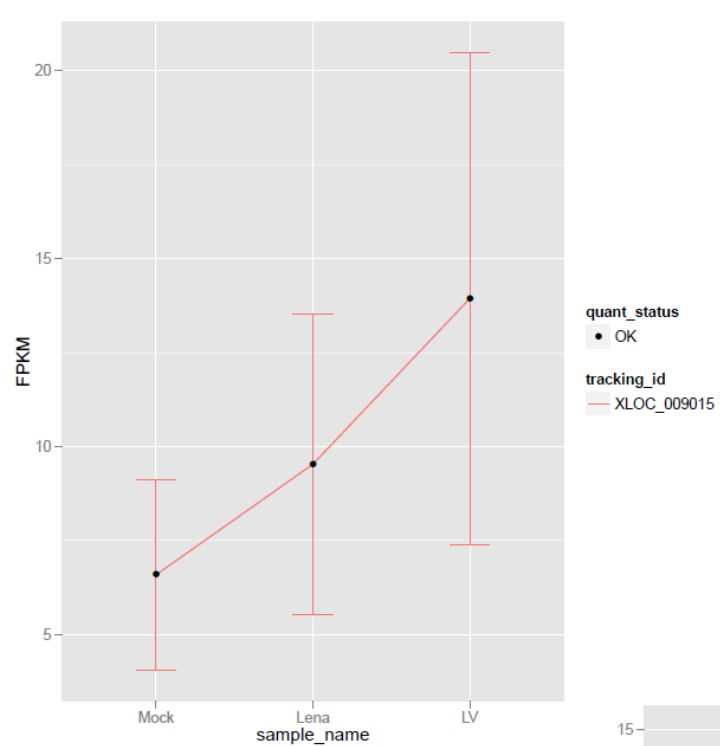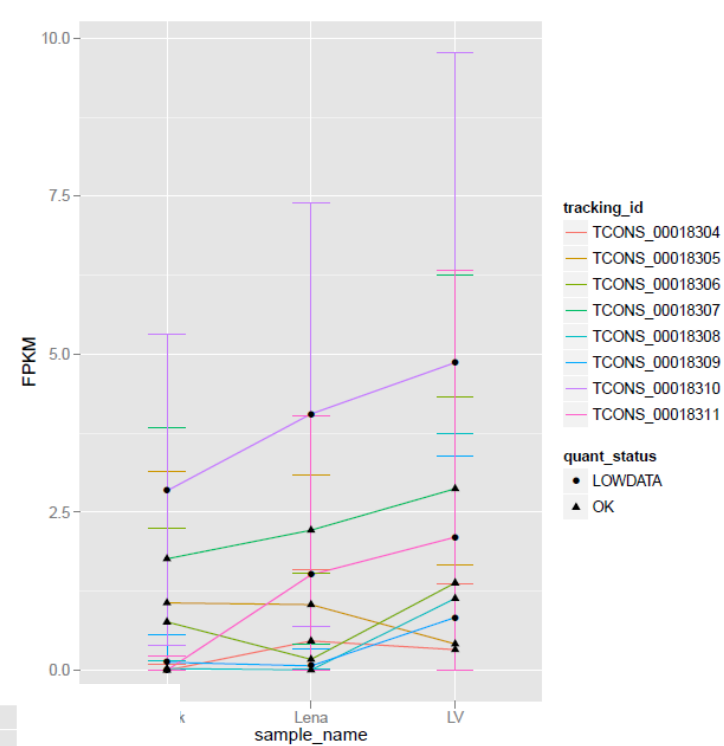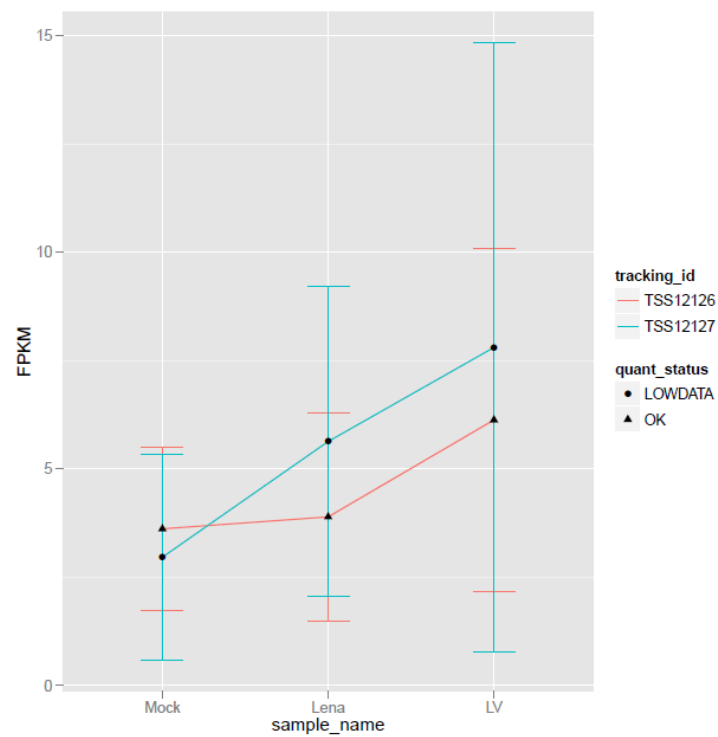

# TLR4

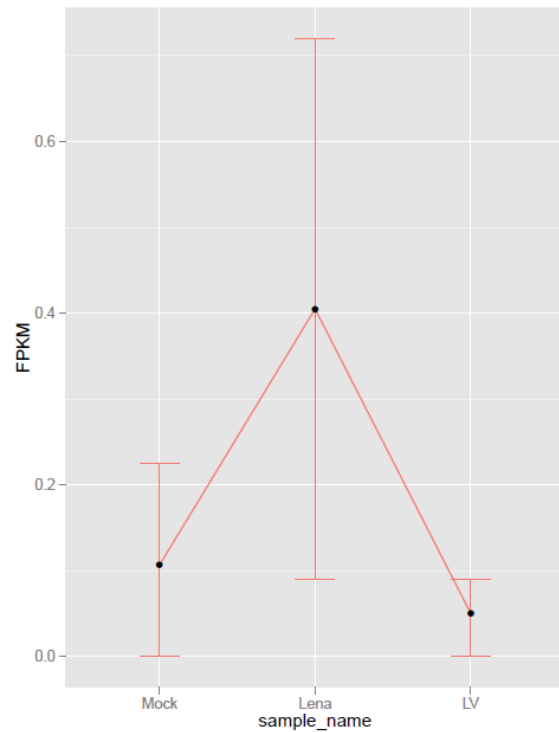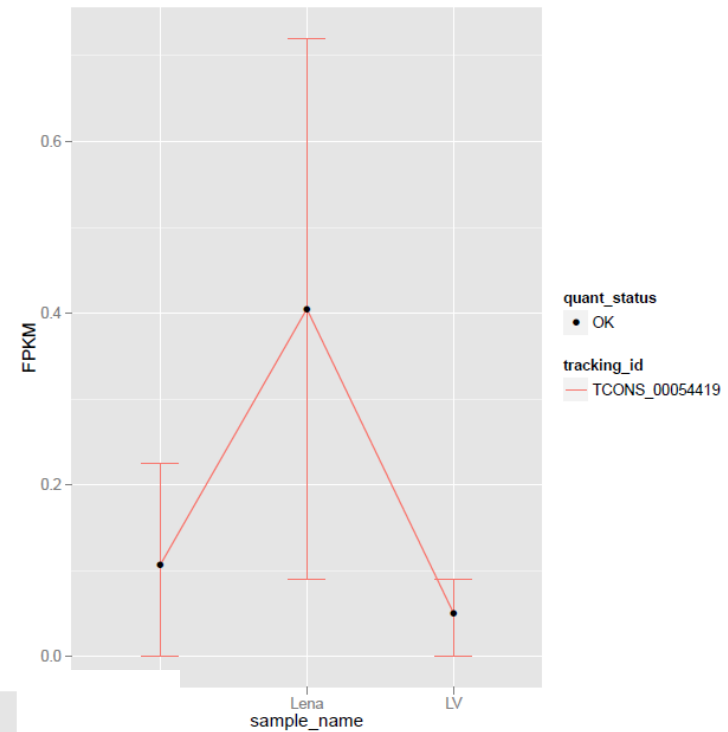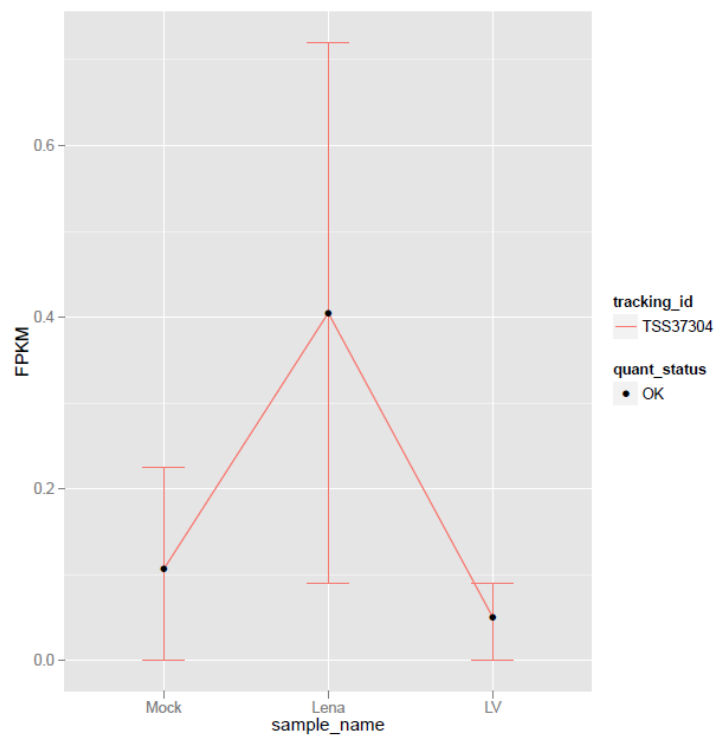

# TLR7

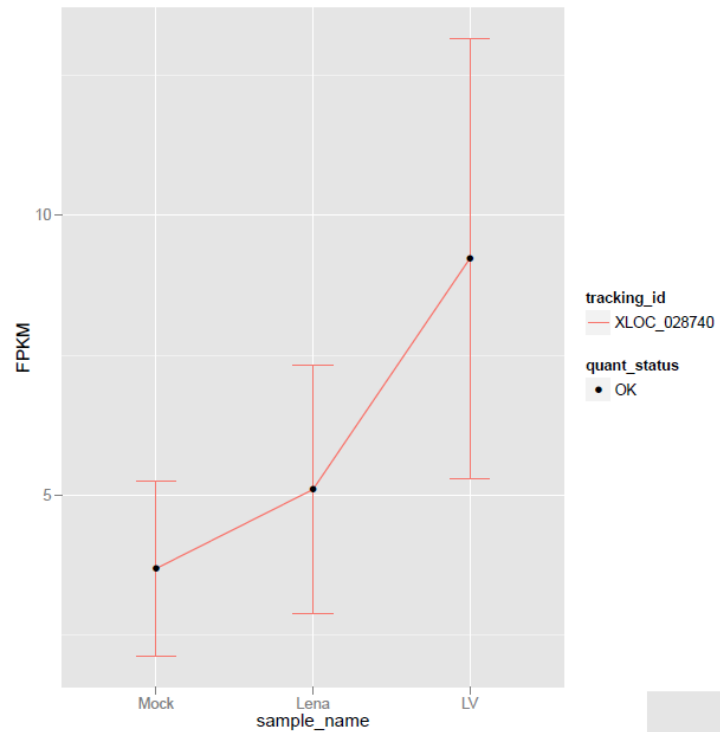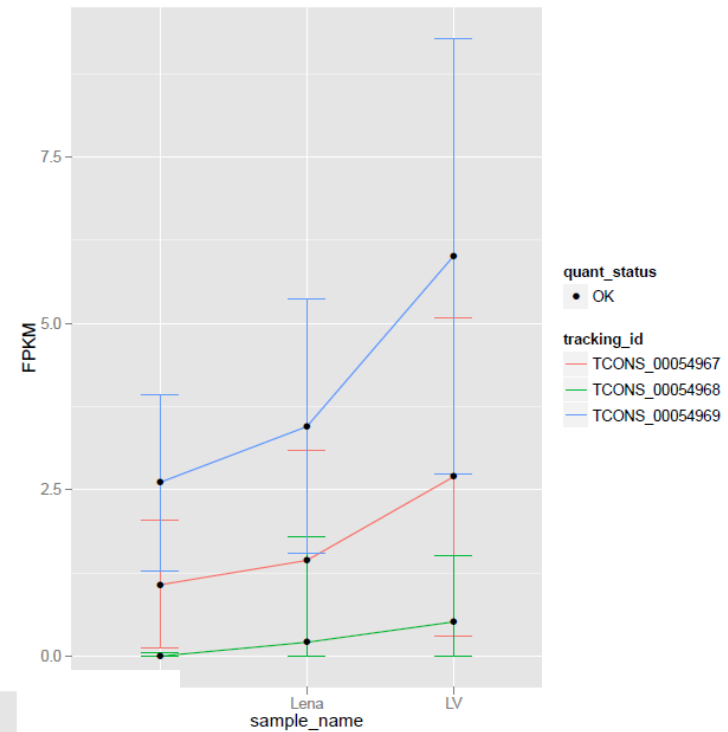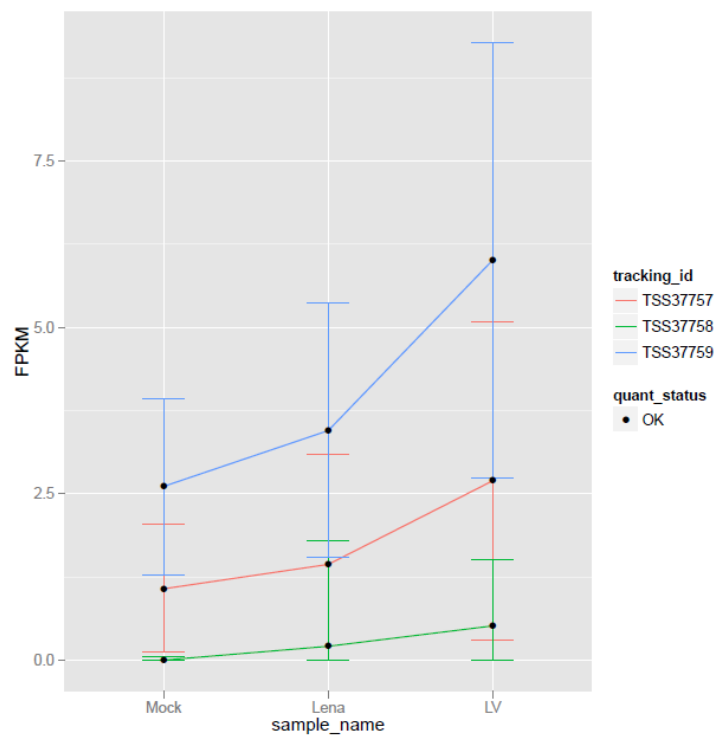

Supplement: Figure S6 — Differential expression ofTLR3, TLR4 and TLR7 between the LV and Lena groups. TLR7 and TLR3 were significantly up-regulated in the LV group, while neither TLR7 nor TLR3wasmodulated in the Lena group. Although TLR4 was expressed in small quantities (medium FPKM = 0.4 in Lena), it was more highly expressed in the Lena group than in the LV group (FC = 5.98) and was expressed at an even lower level during LV infection than during mock infection. (PDF) [file pone.0091918.s006.pdf]

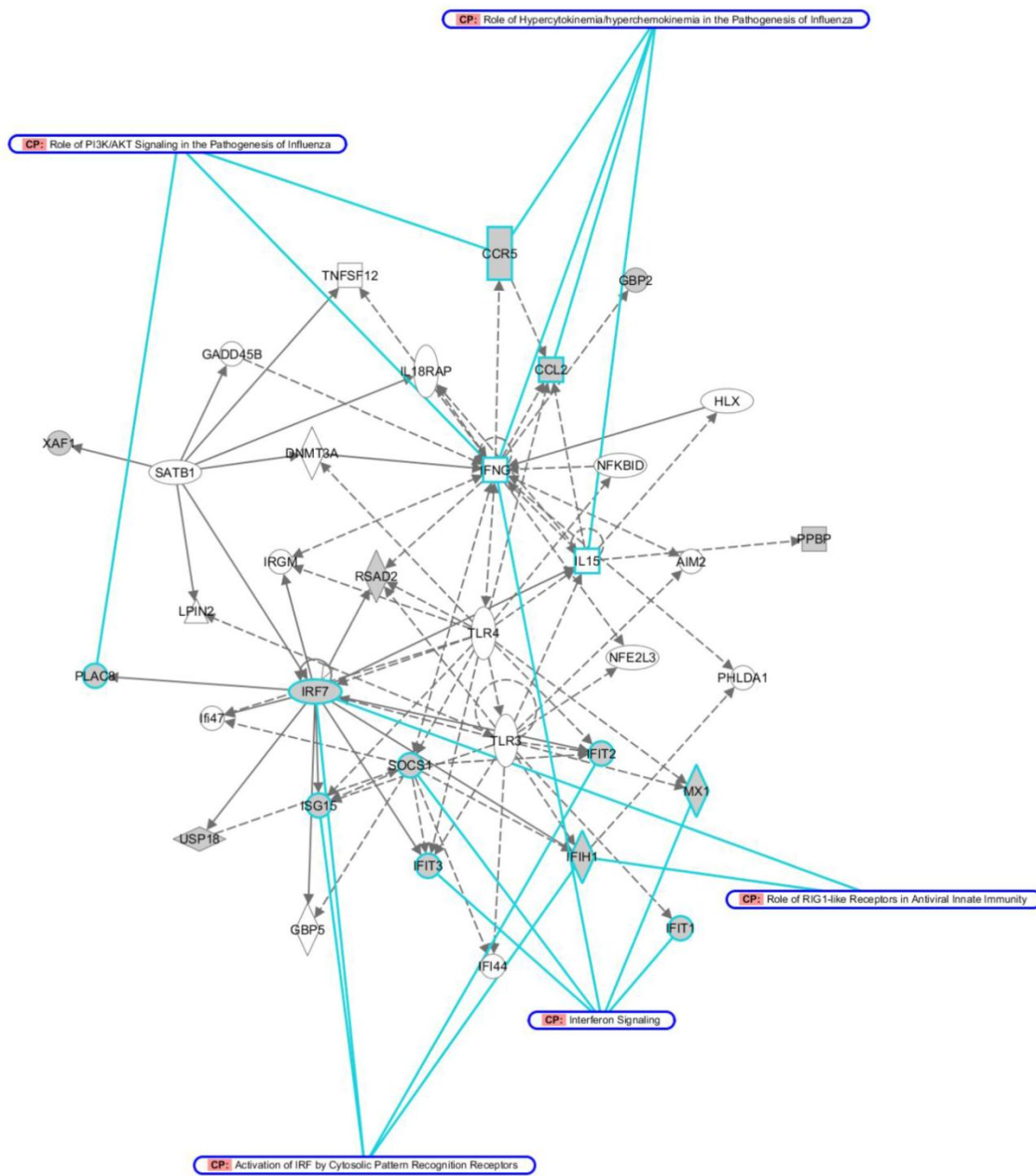

Supplement: Figure S7 — Network formed by common genes that were differentially expressed in the LV vs. mock and Lena vs. mock comparisons from one side and in an in vivo study performed by Zhou et al. (2001) from the other side. The canonical pathways that were significantly affected by this group of genes are highlighted with blue squares and include interferon signaling, the activation of IRF by cytosolic pattern recognition receptors, the role of hypercytokinemia/hyperchemokinemia in the pathogenesis of influenza, the role of RIG1-like receptors in antiviral innate immunity and the role of PI3K/AKT signaling in the pathogenesis of influenza. The networks were constructed using focus molecules as “seeds” that were connected together to form a network using the genes in the list. If needed, other non-focus molecules from the dataset were then added to complete the network. The resulting networks were scored and then sorted based on the score. The network scores represent the negative log of the p-value of the likelihood that the network molecules were found together by chance. Therefore, a high score represents an index indicating that the interconnection of the molecules within the network is more likely to be true. (PDF) [file pone.0091918.s007.pdf]
