# Supplementary material for: RNA-Sequence Analysis of Primary Alveolar Macrophages after In Vitro Infection with Porcine Reproductive and Respiratory Syndrome Virus Strains of Differing Virulence
Source: PLoS One. 2014 Mar 18;9(3):e91918. doi: 10.1371/journal.pone.0091918 (PMC3958415; doi:10.1371/journal.pone.0091918)
Supplement: Figure S5 — Transcriptional/post-transcriptional regulation of the genes involved in the top canonical pathways in the LV vs. mock, Lena vs. mock and LV vs. Lena comparisons. (A) Un-spliced and transcriptionally regulated genes,(B) spliced and post-transcriptionally regulated genes and(C) spliced and both transcriptionally and post-transcriptionally regulated genes. For each transcript,the “XLOC”, “TSS” and “TCONS” suffixes correspond to the genes, TSSs and isoforms, respectively. Differentially expressed isoforms with different TSSs are transcriptionally regulated, while isoforms with the same TSS are regulated at the post-transcriptional level (Figure S4). (PDF) [file pone.0091918.s005.pdf]

# Group 1: un-spliced and transcriptionally regulated genes

# CCL4

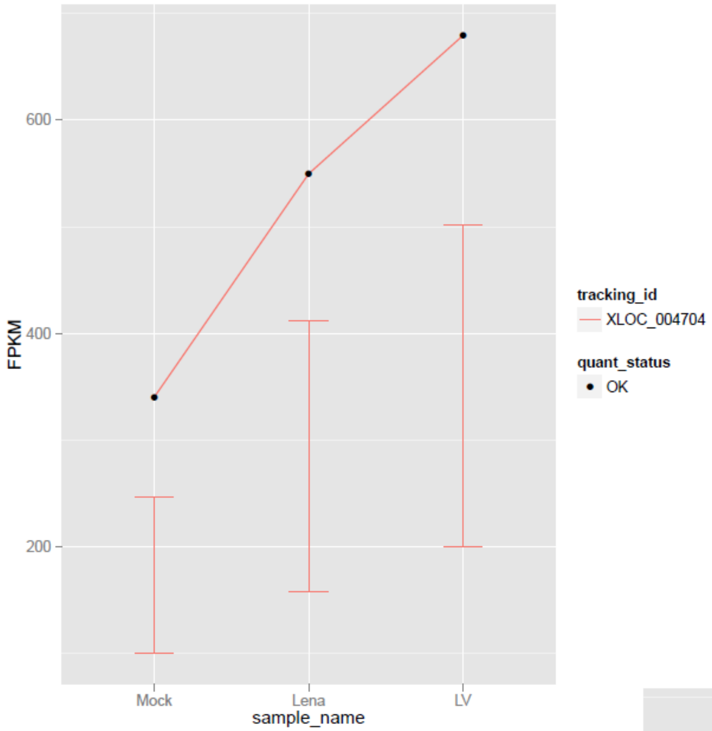
600
400
200
FPKM
Mock
Lena
LV
sample_name
tracking_id
XLOC_004704
quant_status
OK

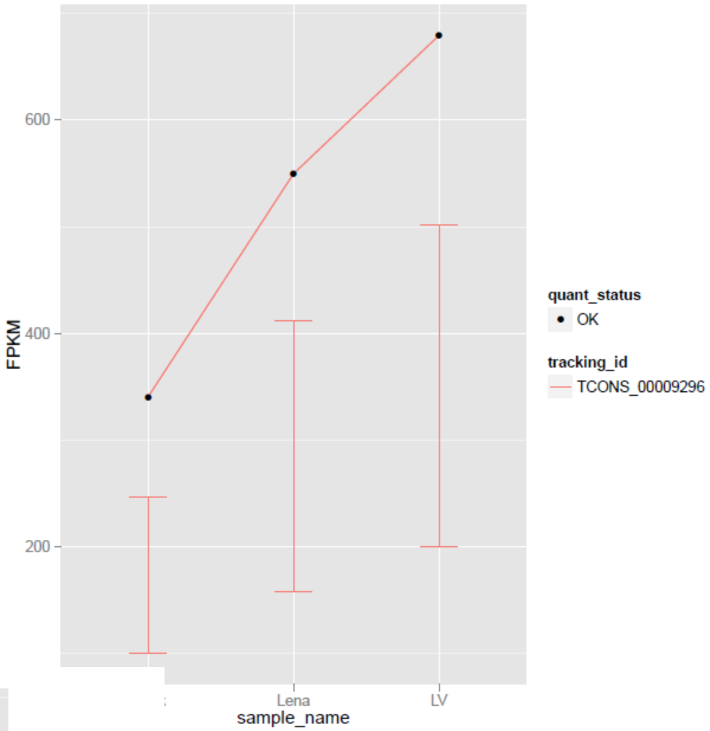
600
400
200
FPKM
Lena
LV
sample_name
quant_status
OK
tracking_id
TCONS_00009296

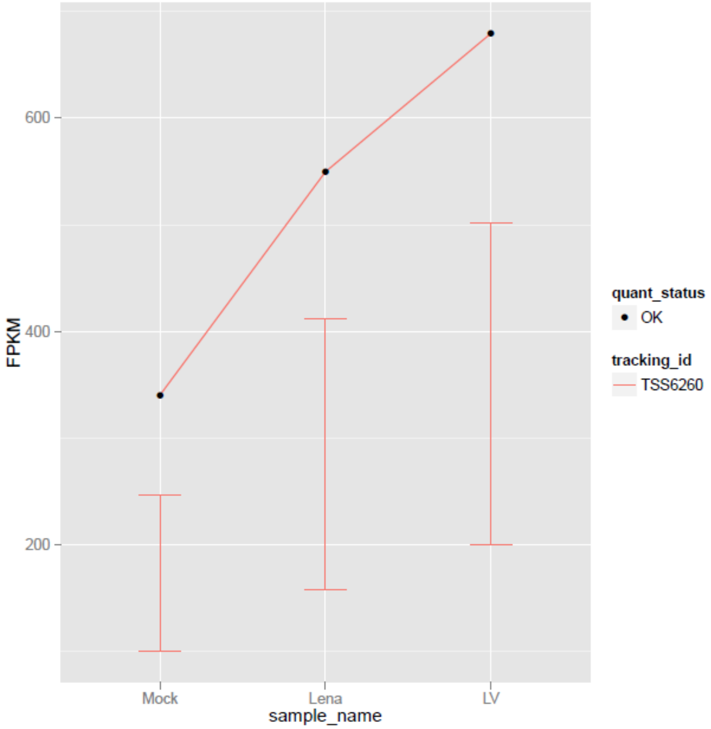
600
400
200
FPKM
Mock
Lena
LV
sample_name
quant_status
OK
tracking_id
TSS6260

# CCL2

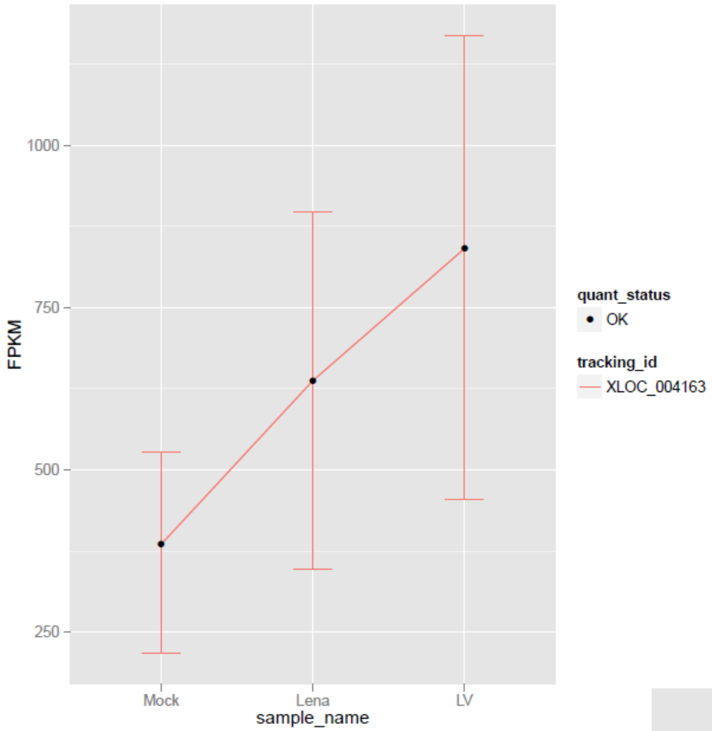

FPKM
1000
750
500
250
Mock
Lena
LV
sample_name
quant_status
OK
tracking_id
XLOC_004163


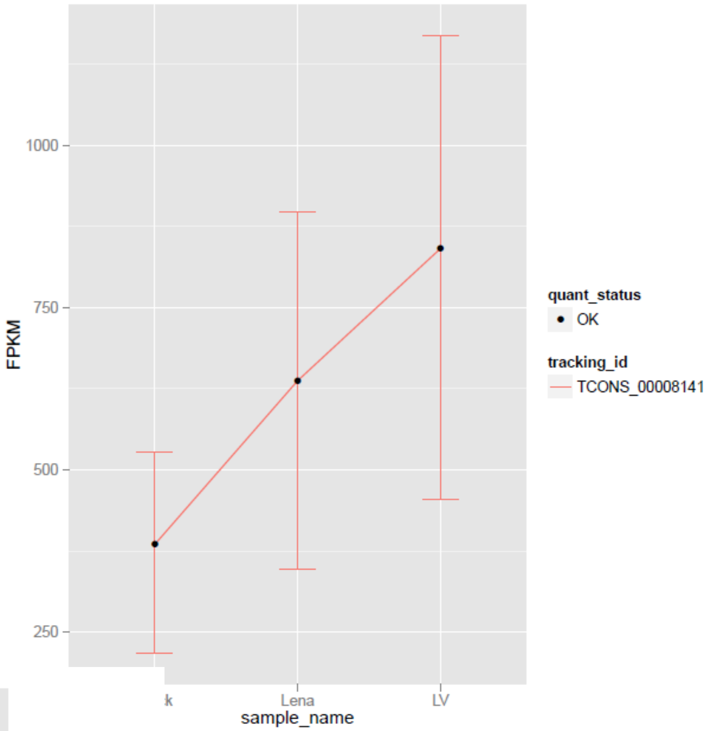

FPKM
1000
750
500
250
k
Lena
LV
sample_name
quant_status
OK
tracking_id
TCONS_00008141


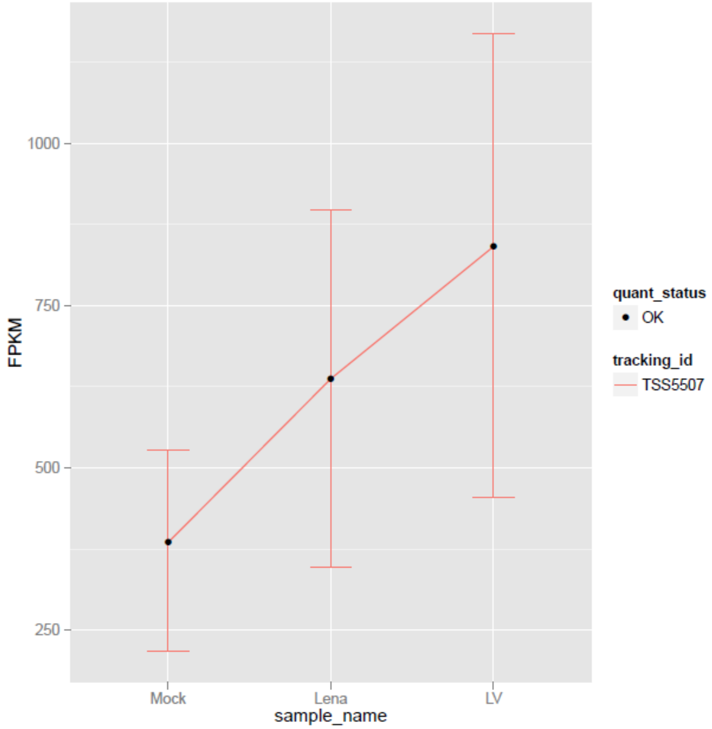

FPKM
1000
750
500
250
Mock
Lena
LV
sample_name
quant_status
OK
tracking_id
TSS5507

# CCR5

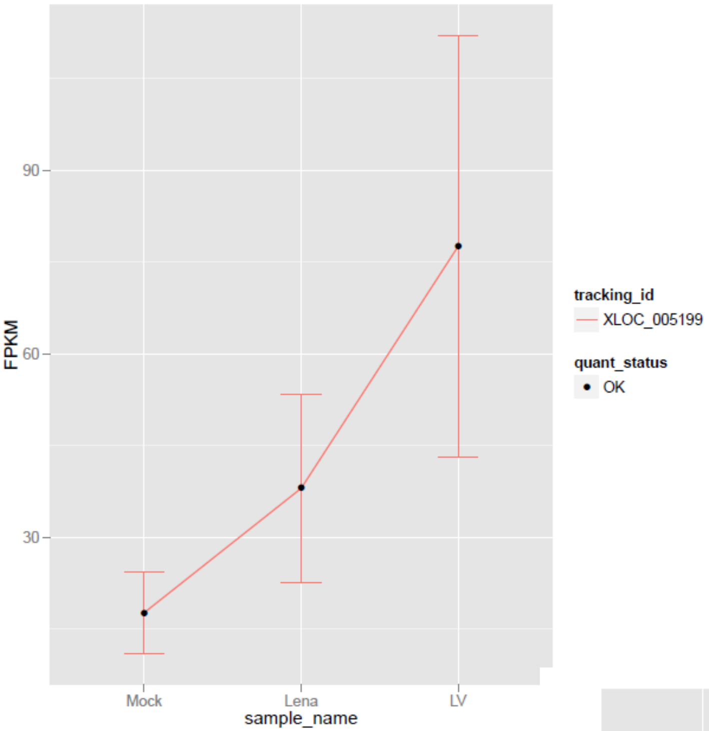

FPKM
90
60
30
Mock
Lena
LV
sample_name
tracking_id
XLOC_005199
quant_status
OK


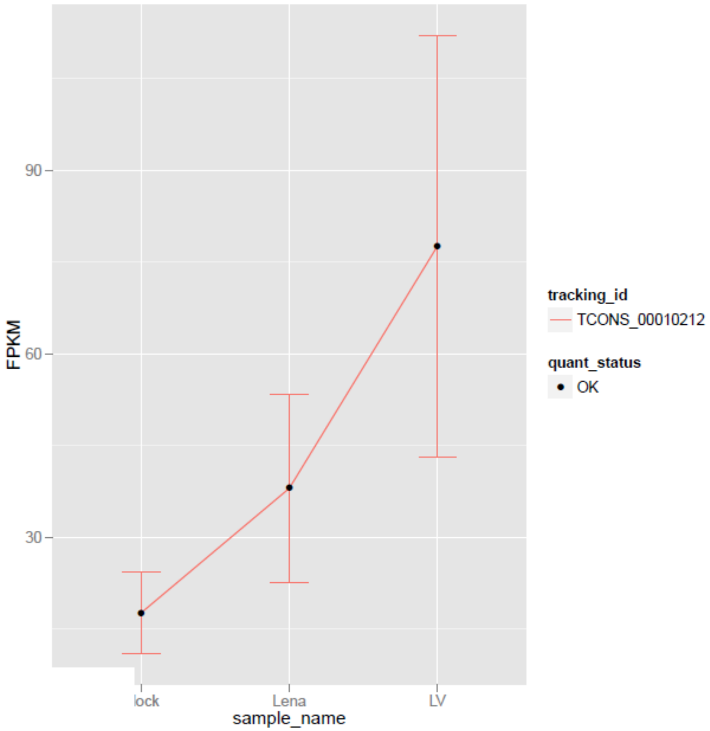

FPKM
90
60
30
Lena
LV
sample_name
tracking_id
TCONS_00010212
quant_status
OK


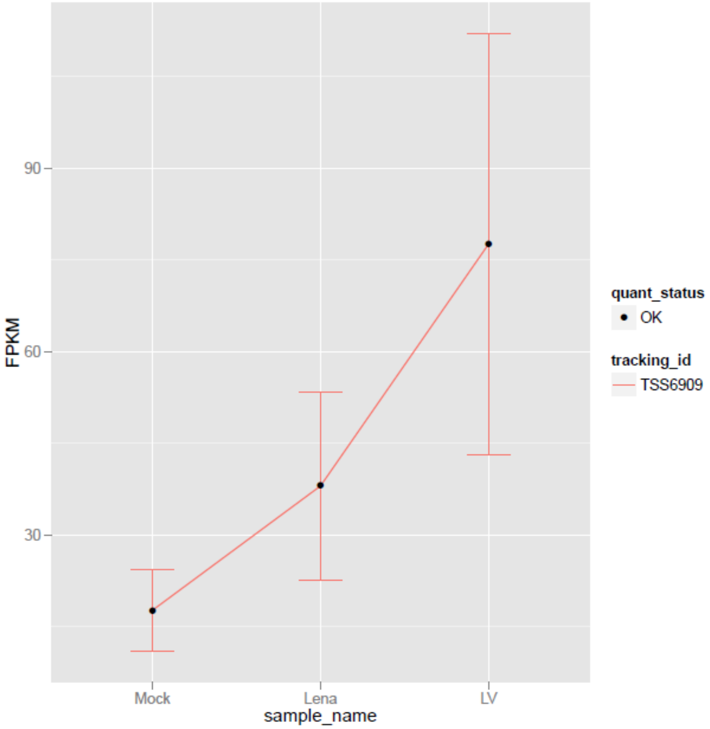

FPKM
90
60
30
Mock
Lena
LV
sample_name
quant_status
OK
tracking_id
TSS6909

# IL8

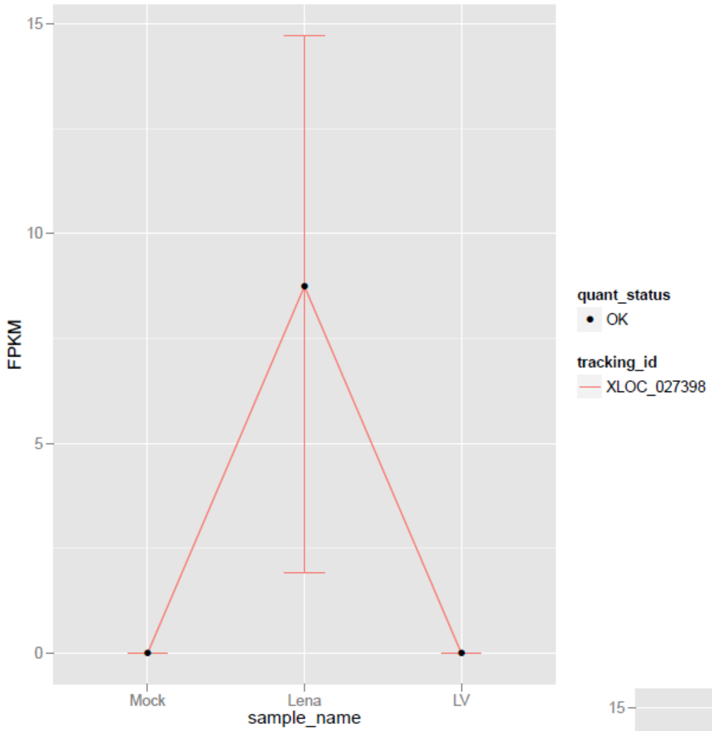

FPKM
15
10
5
0
Mock
Lena
LV
sample_name
quant_status
OK
tracking_id
XLOC_027398


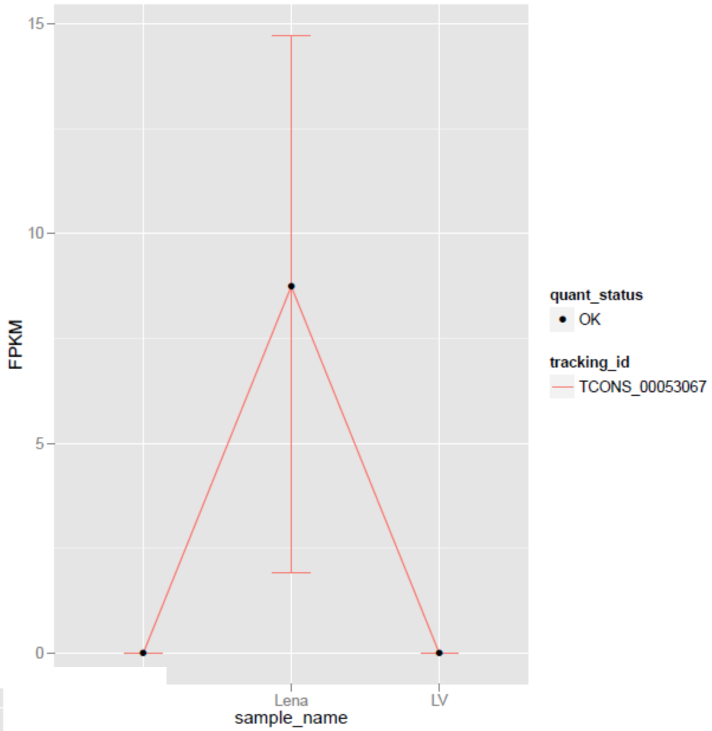

FPKM
15
10
5
0
Lena
LV
sample_name
quant_status
OK
tracking_id
TCONS_00053067


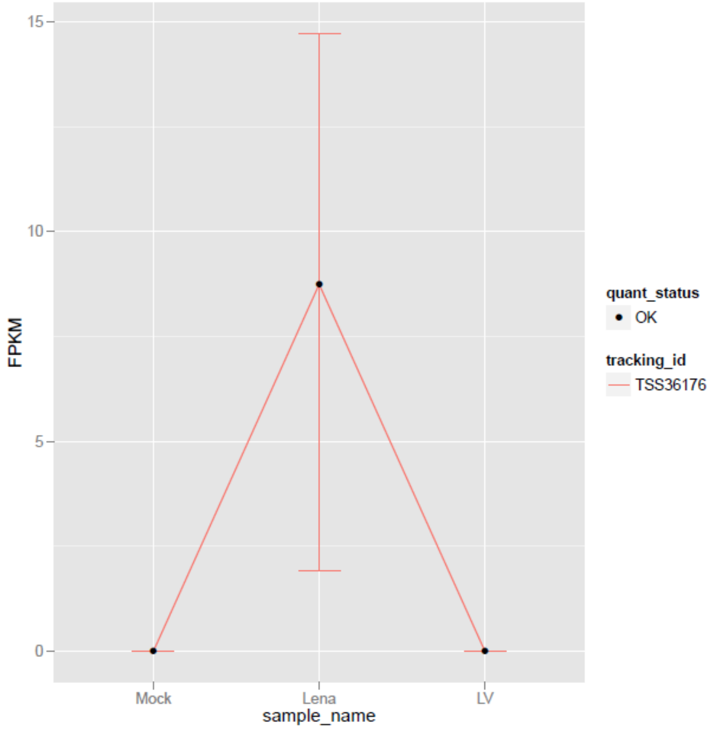

FPKM
15
10
5
0
Mock
Lena
LV
sample_name
quant_status
OK
tracking_id
TSS36176

# TNF

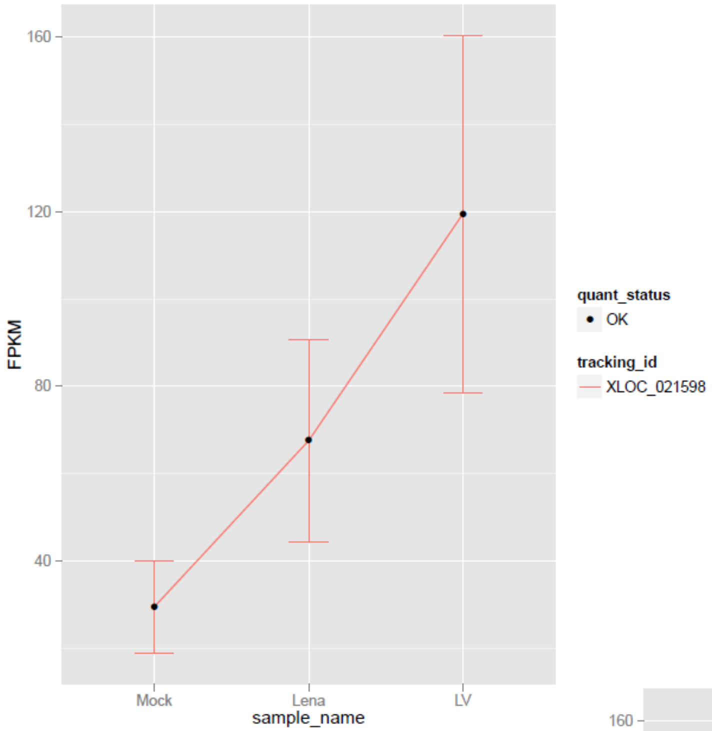

160
120
80
40
FPKM
Mock
Lena
LV
sample_name
quant_status
OK
tracking_id
XLOC_021598


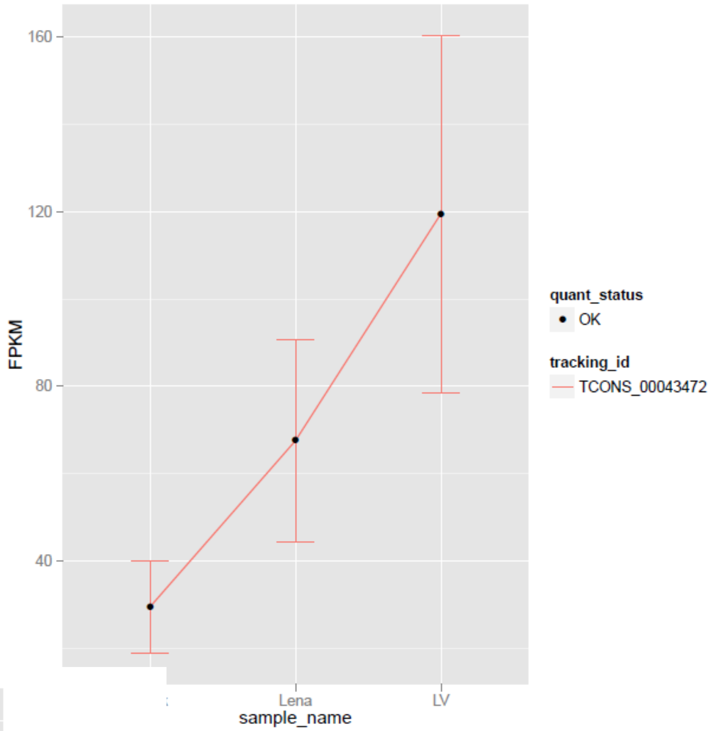

160
120
80
40
FPKM
Lena
LV
sample_name
quant_status
OK
tracking_id
TCONS_00043472


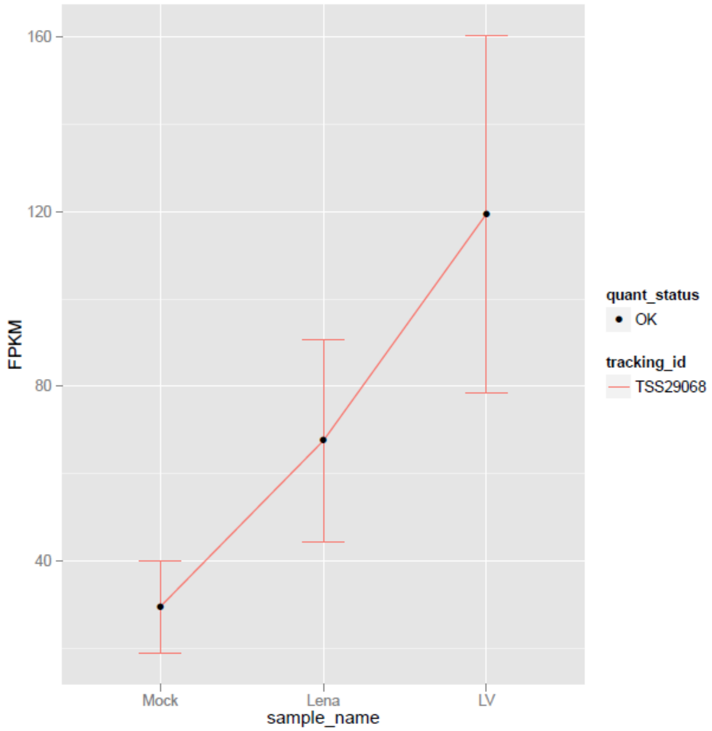

160
120
80
40
FPKM
Mock
Lena
LV
sample_name
quant_status
OK
tracking_id
TSS29068

# BID

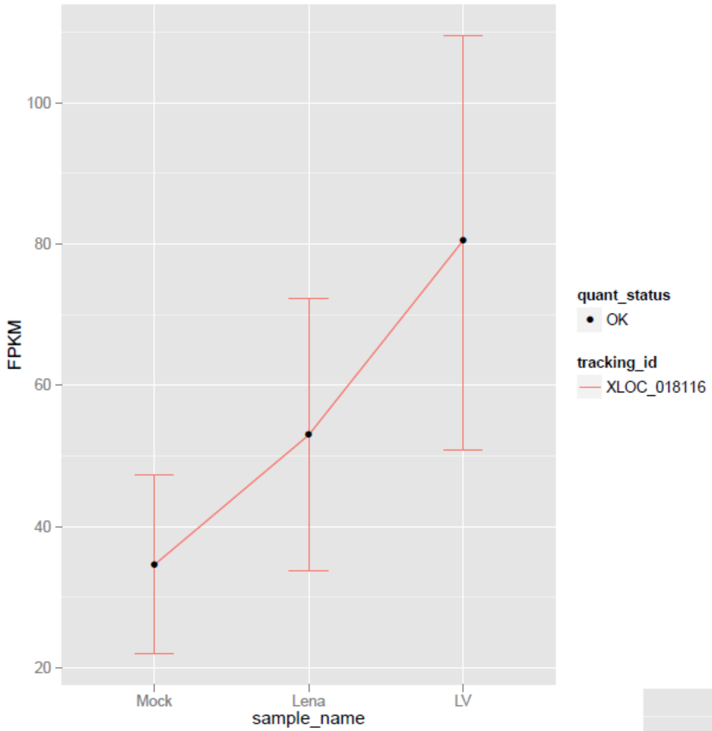

100
80
60
40
20
FPKM
Mock
Lena
LV
sample_name
quant_status
OK
tracking_id
XLOC_018116


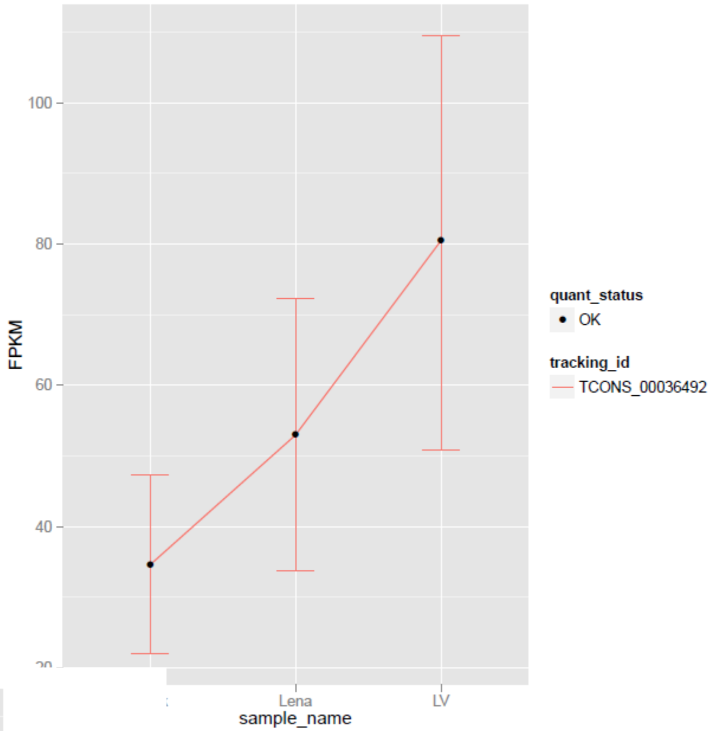

100
80
60
40
FPKM
Lena
LV
sample_name
quant_status
OK
tracking_id
TCONS_00036492


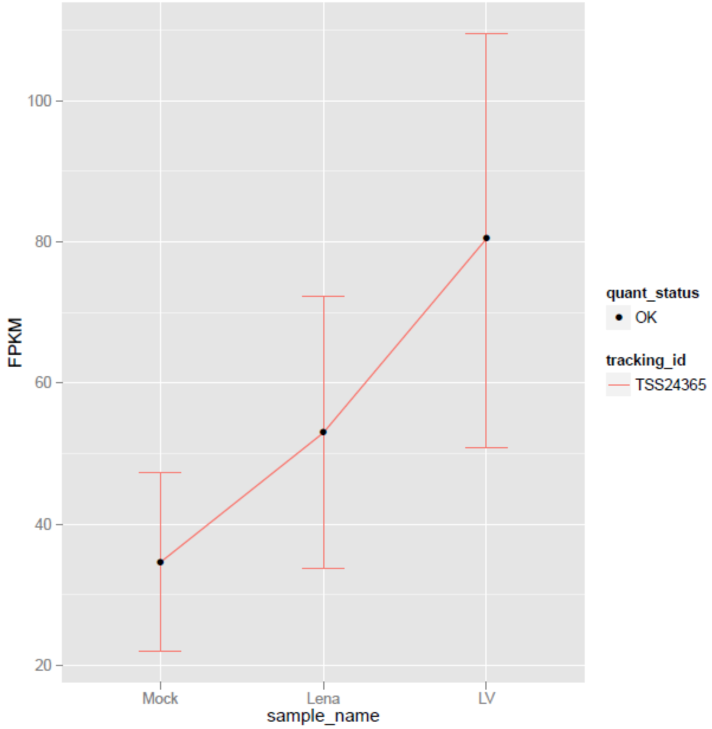

100
80
60
40
20
FPKM
Mock
Lena
LV
sample_name
quant_status
OK
tracking_id
TSS24365

# ZC3HAV1

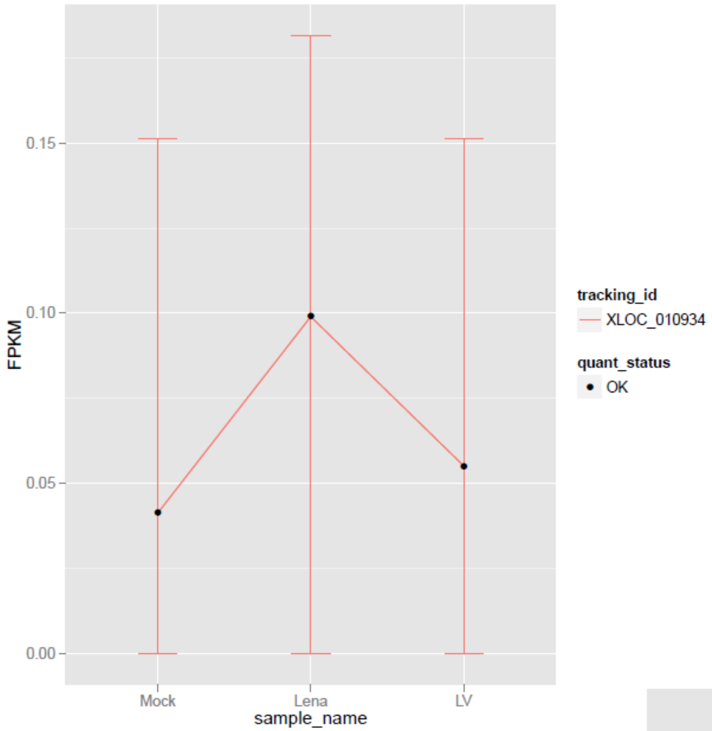

0.15
0.10
0.05
0.00
FPKM
Mock
Lena
LV
sample_name
tracking_id
XLOC_010934
quant_status
OK


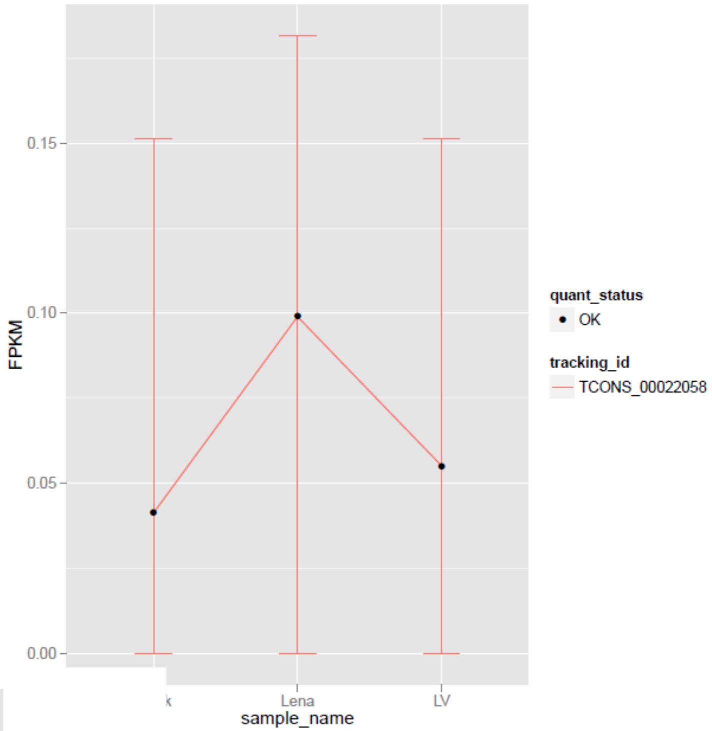

0.15
0.10
0.05
0.00
FPKM
Lena
LV
sample_name
quant_status
OK
tracking_id
TCONS_00022058


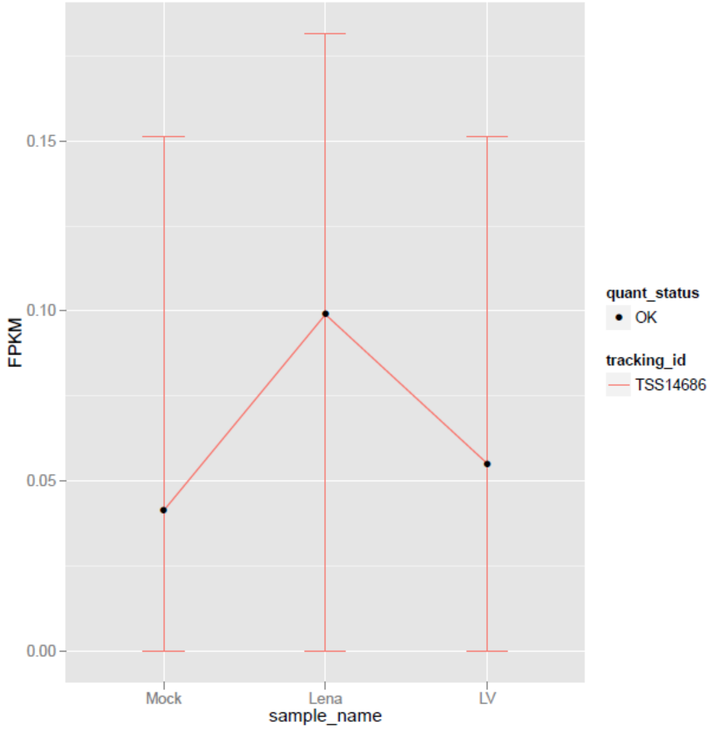

0.15
0.10
0.05
0.00
FPKM
Mock
Lena
LV
sample_name
quant_status
OK
tracking_id
TSS14686

# CASP8

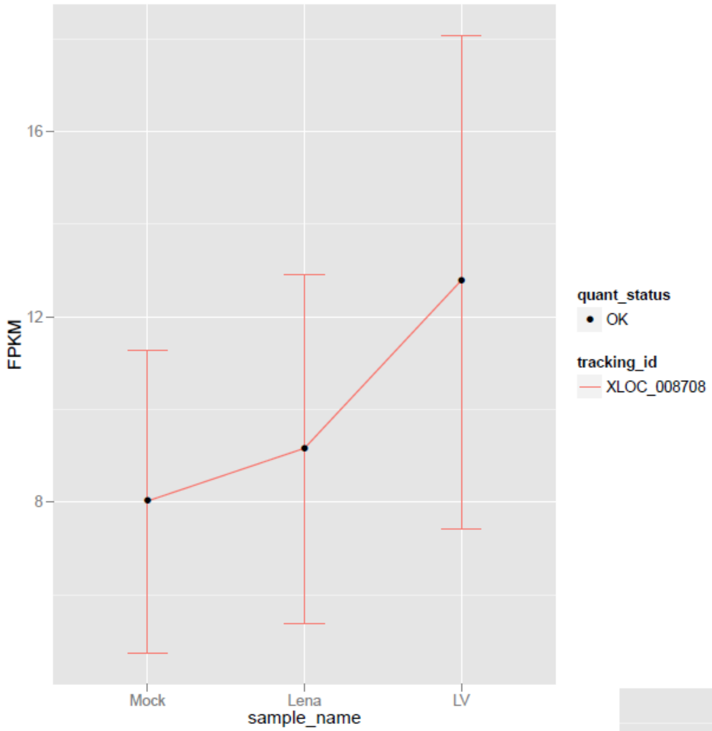
16
12
8
FPKM
Mock
Lena
LV
sample_name
quant_status
OK
tracking_id
XLOC_008708

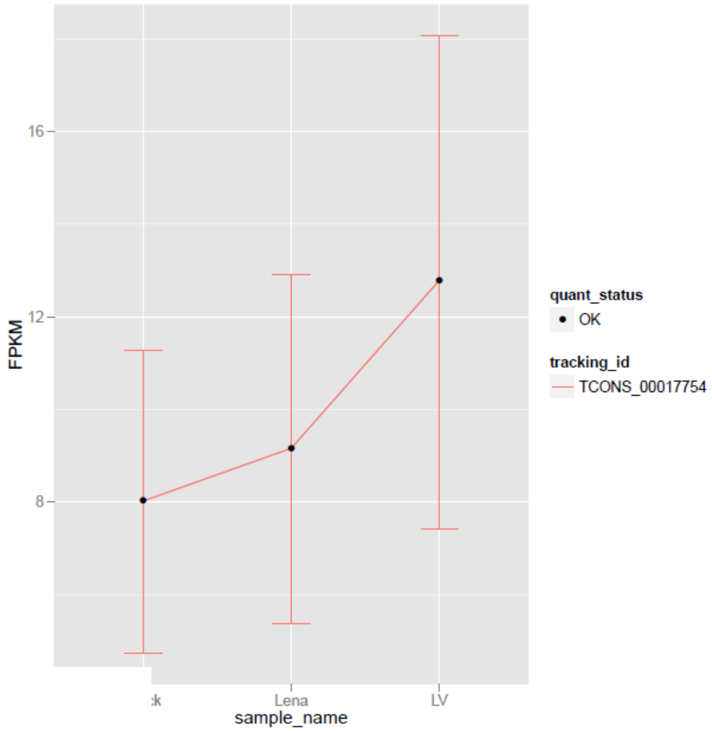
16
12
8
FPKM
Lena
LV
sample_name
quant_status
OK
tracking_id
TCONS_00017754

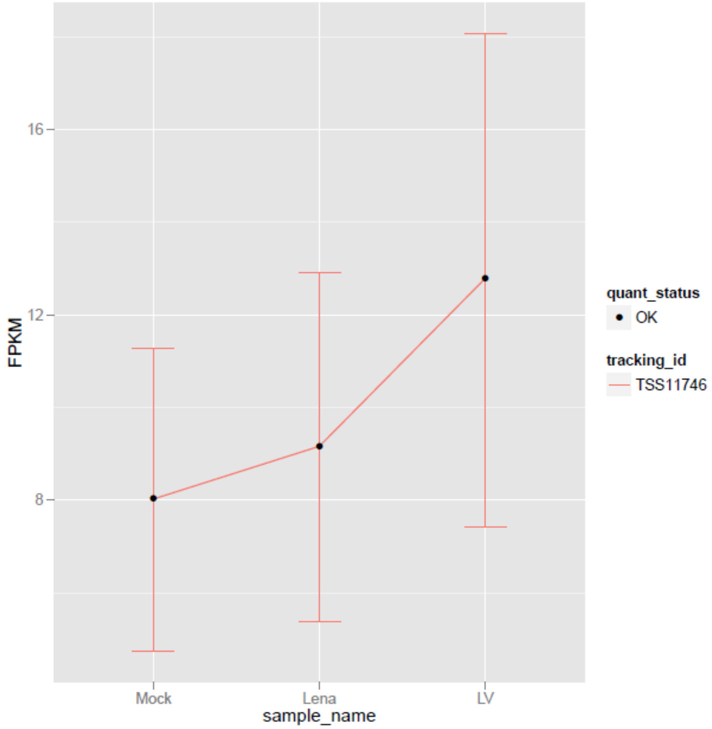
16
12
8
FPKM
Mock
Lena
LV
sample_name
quant_status
OK
tracking_id
TSS11746

# TLR4

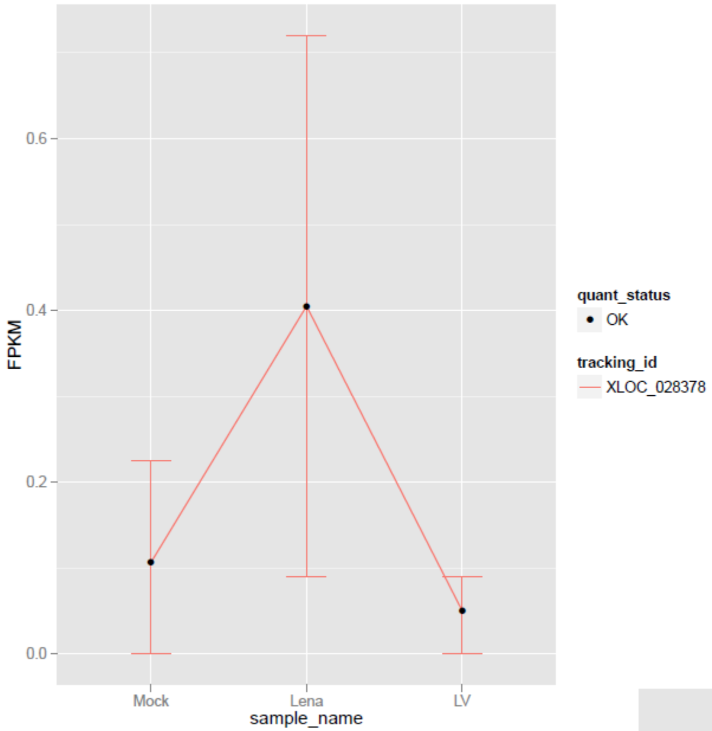

FPKM
0.0
0.2
0.4
0.6
Mock
Lena
LV
sample_name
quant_status
OK
tracking_id
XLOC_028378


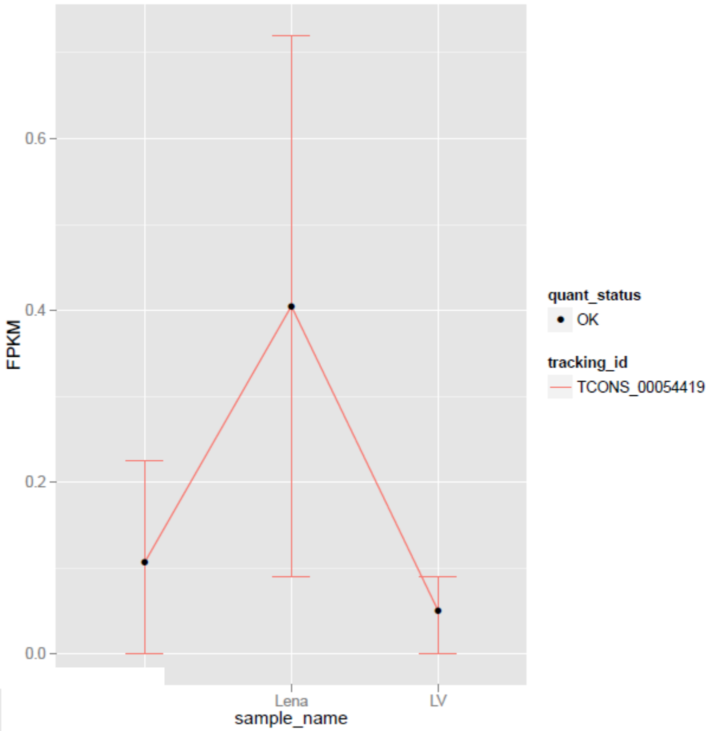

FPKM
0.0
0.2
0.4
0.6
Lena
LV
sample_name
quant_status
OK
tracking_id
TCONS_00054419


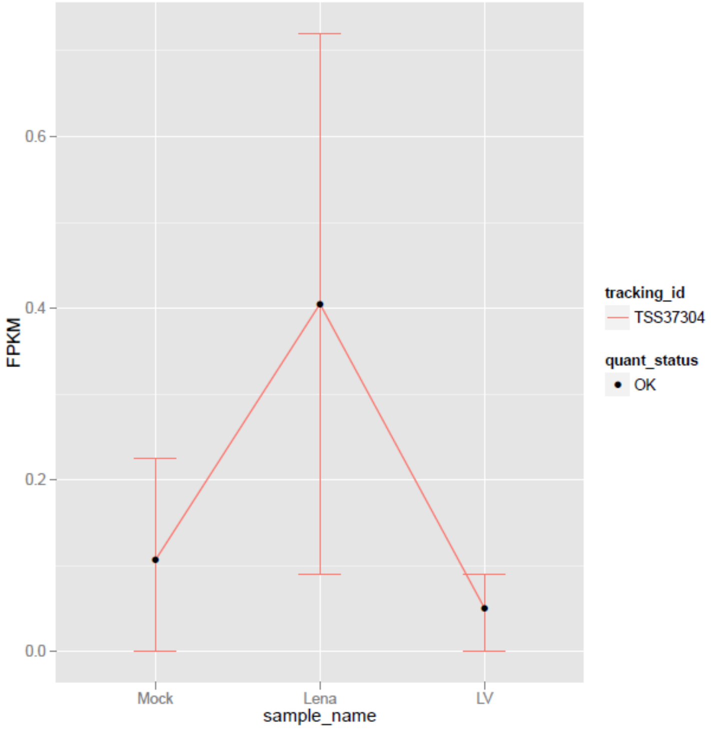

FPKM
0.0
0.2
0.4
0.6
Mock
Lena
LV
sample_name
tracking_id
TSS37304
quant_status
OK

# Group 2: Spliced and post-transcriptionally regulated" genes

# SOCS1

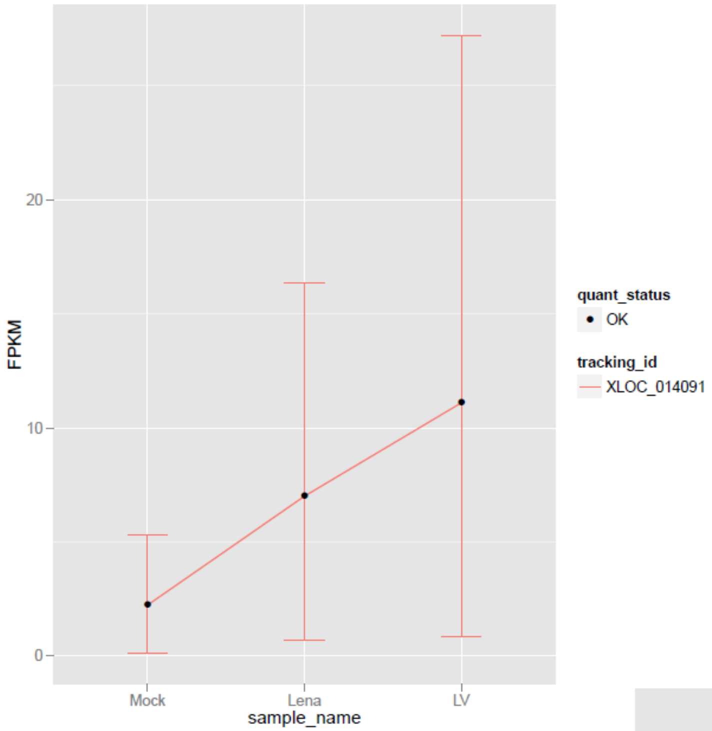
FPKM
0
10
20
Mock
Lena
LV
sample_name
quant_status
OK
tracking_id
XLOC_014091

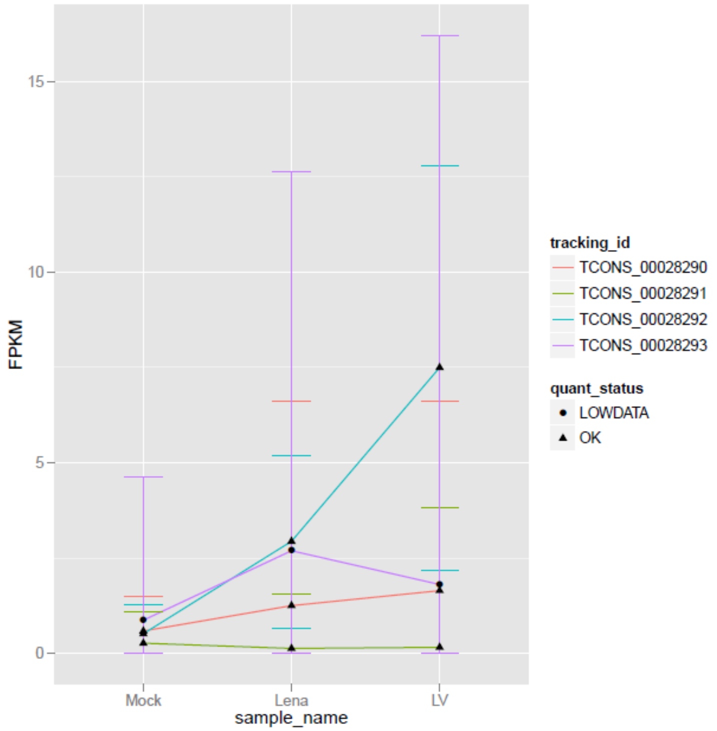
FPKM
0
5
10
15
Mock
Lena
LV
sample_name
tracking_id
TCONS_00028290
TCONS_00028291
TCONS_00028292
TCONS_00028293
quant_status
LOWDATA
OK

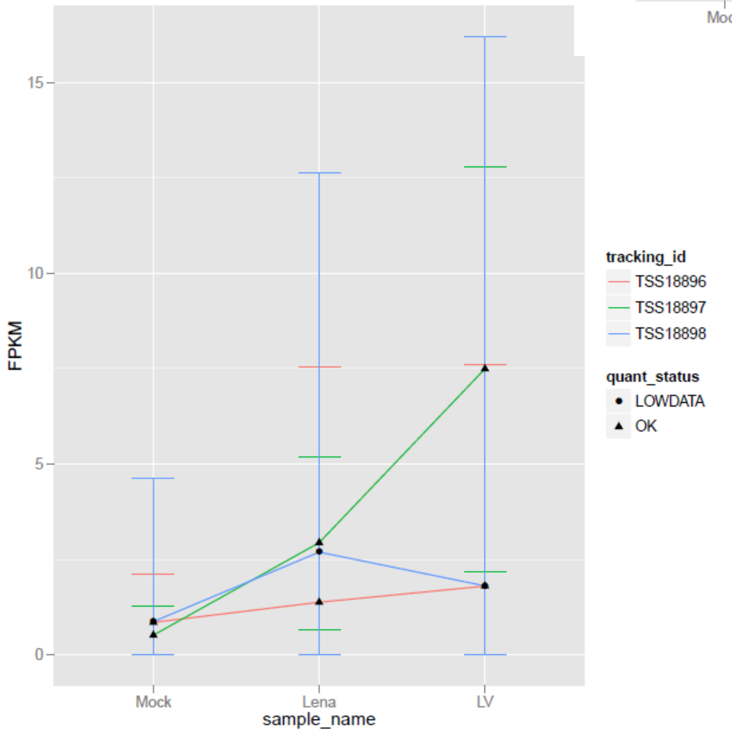
FPKM
0
5
10
15
Mock
Lena
LV
sample_name
tracking_id
TSS18896
TSS18897
TSS18898
quant_status
LOWDATA
OK

# STAT2

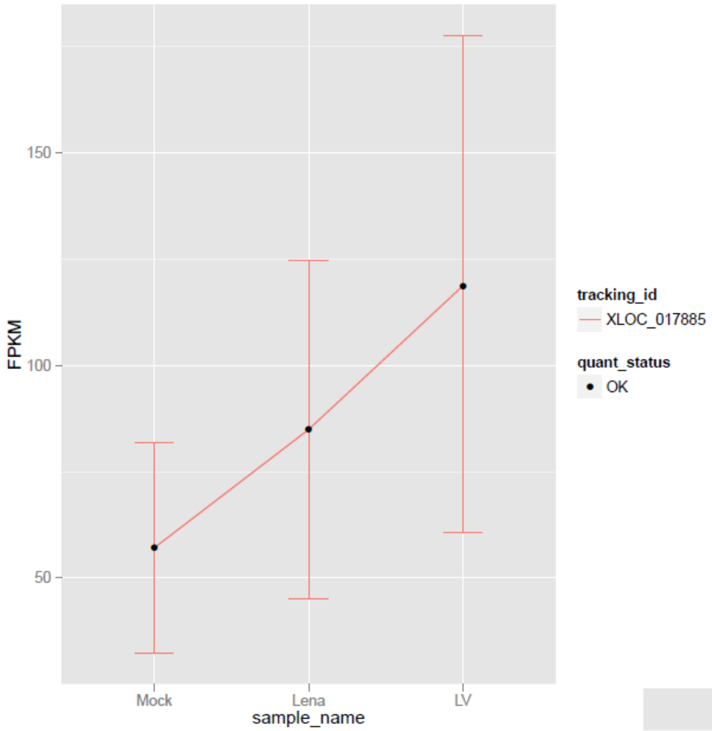

FPKM
150
100
50
Mock
Lena
LV
sample_name
tracking_id
XLOC_017885
quant_status
OK


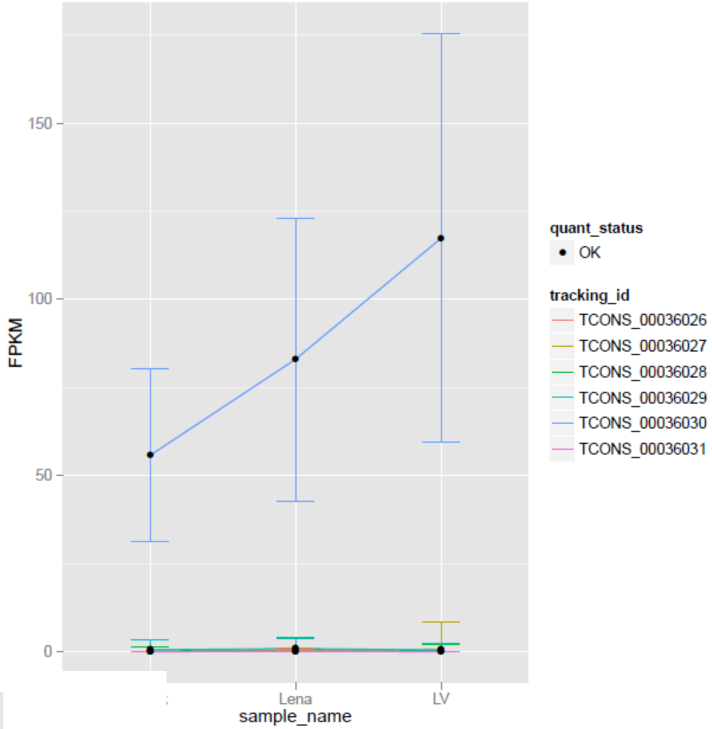

FPKM
150
100
50
0
Lena
LV
sample_name
quant_status
OK
tracking_id
TCONS_00036026
TCONS_00036027
TCONS_00036028
TCONS_00036029
TCONS_00036030
TCONS_00036031


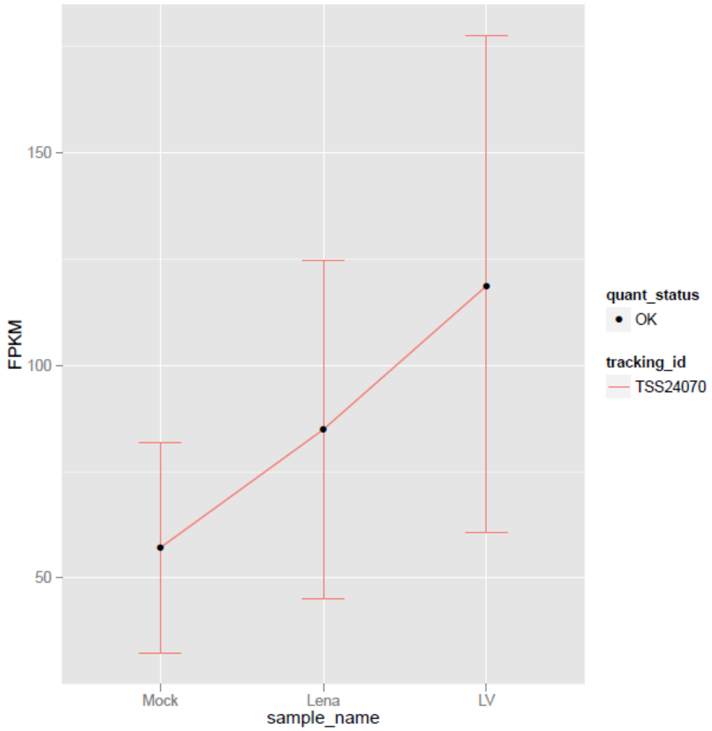

FPKM
150
100
50
Mock
Lena
LV
sample_name
quant_status
OK
tracking_id
TSS24070

# OAS2

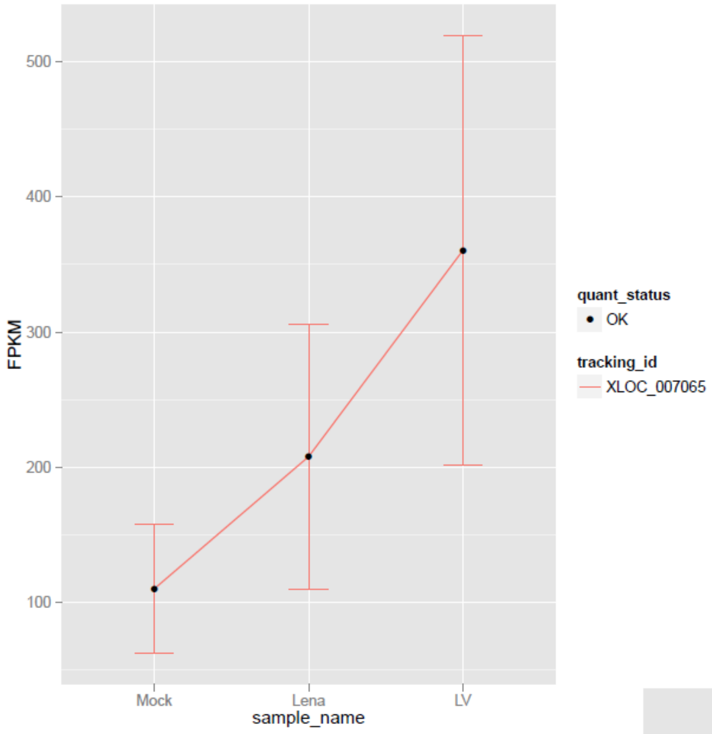

FPKM
500
400
300
200
100
Mock
Lena
LV
sample_name
quant_status
OK
tracking_id
XLOC_007065


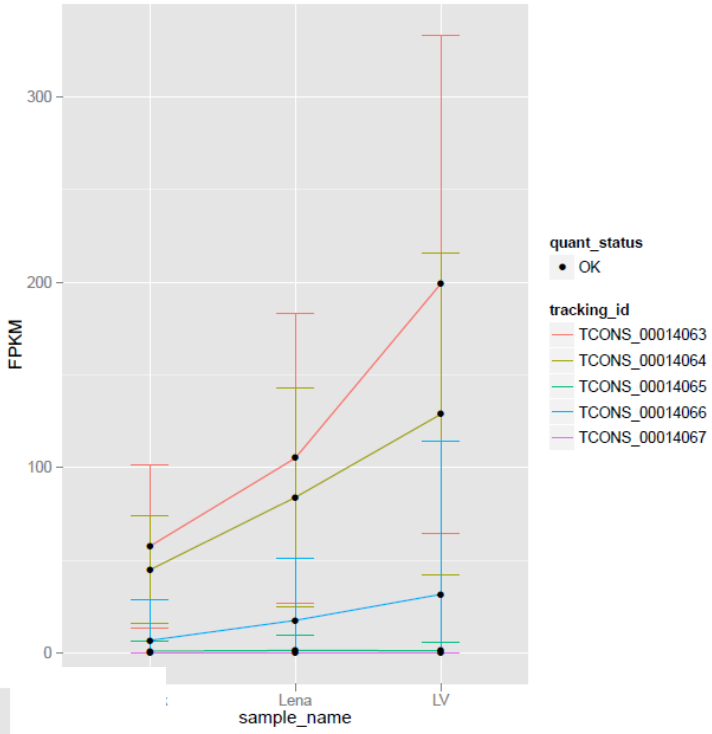

FPKM
300
200
100
0
Lena
LV
sample_name
quant_status
OK
tracking_id
TCONS_00014063
TCONS_00014064
TCONS_00014065
TCONS_00014066
TCONS_00014067


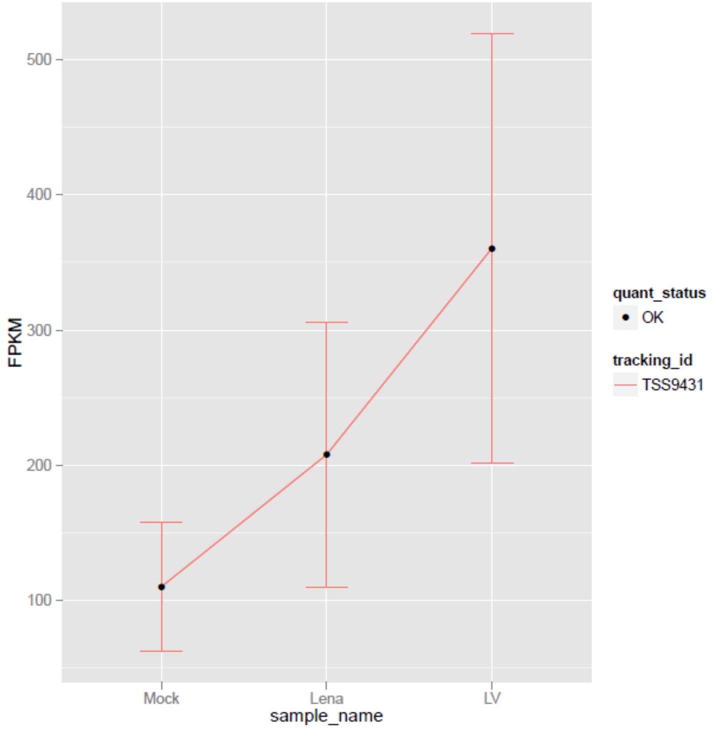

FPKM
500
400
300
200
100
Mock
Lena
LV
sample_name
quant_status
OK
tracking_id
TSS9431

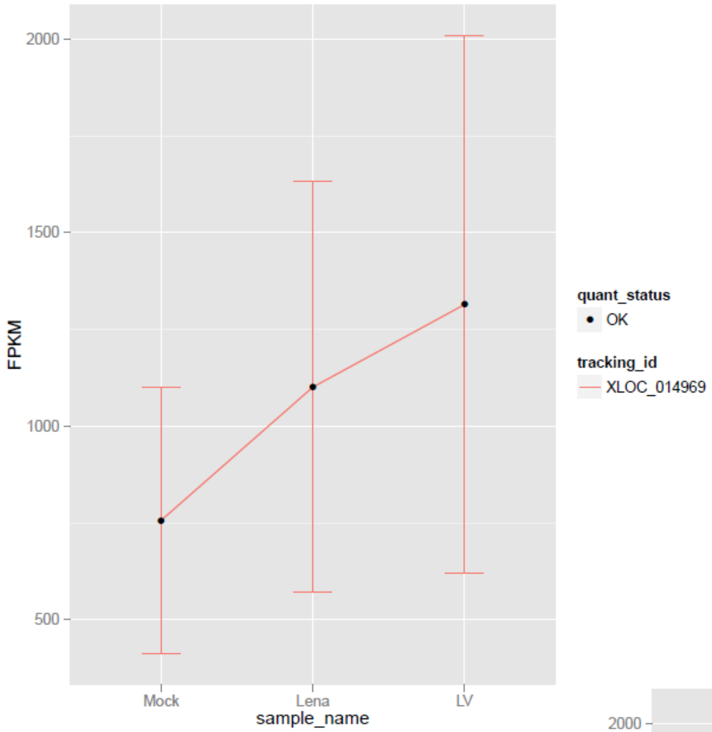

2000
1500
1000
500
FPKM
Mock
Lena
LV
sample_name
quant_status
OK
tracking_id
XLOC_014969


# IL1B

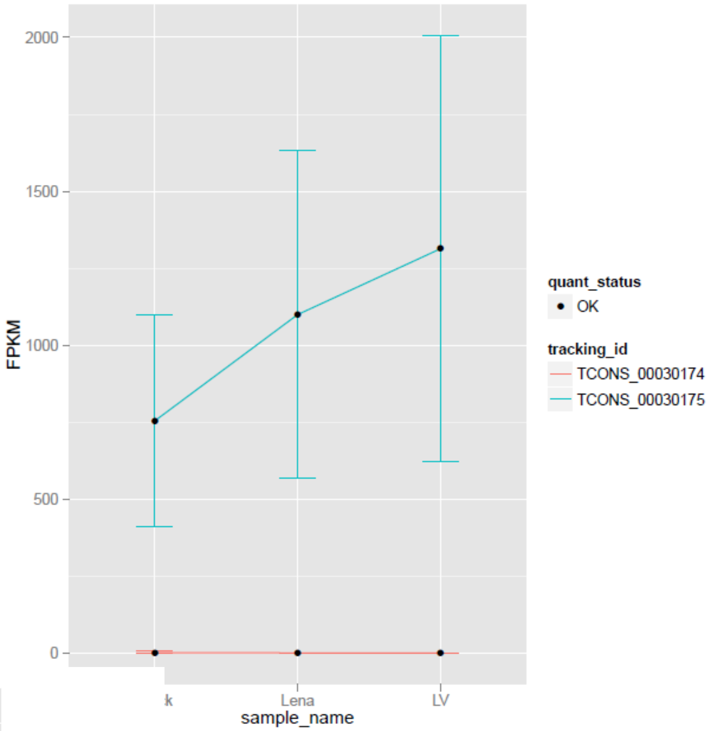

2000
1500
1000
500
0
FPKM
k
Lena
LV
sample_name
quant_status
OK
tracking_id
TCONS_00030174
TCONS_00030175


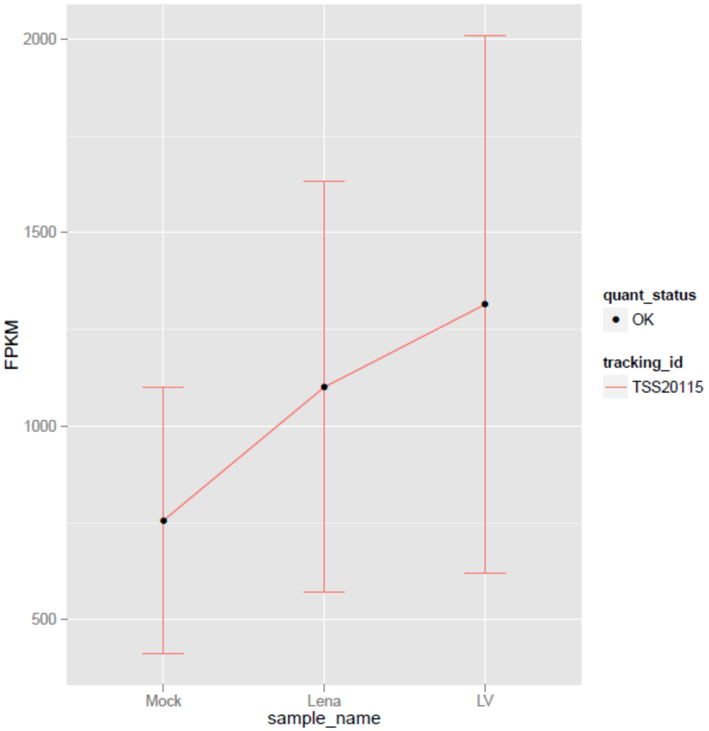

2000
1500
1000
500
FPKM
Mock
Lena
LV
sample_name
quant_status
OK
tracking_id
TSS20115

# CASP1

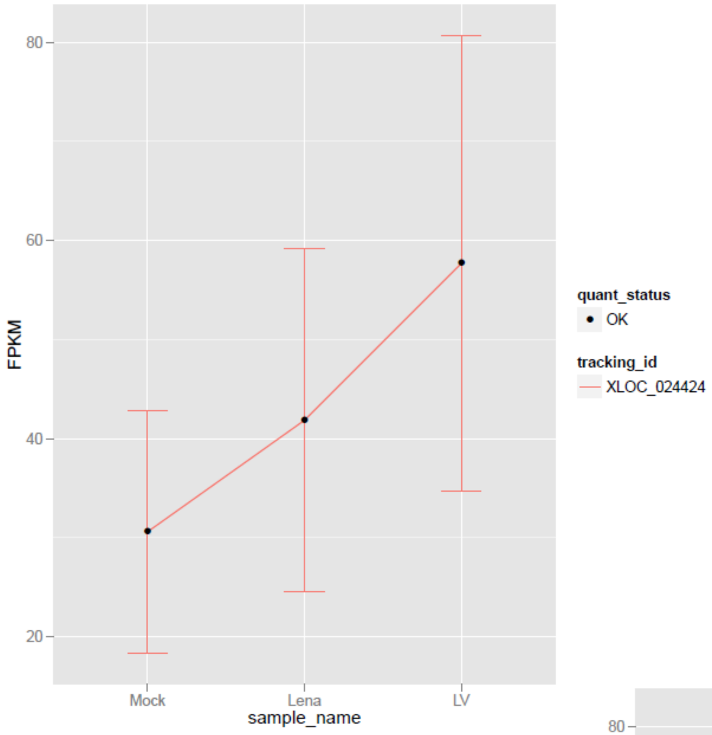

FPKM
80
60
40
20
Mock
Lena
LV
sample_name
quant_status
OK
tracking_id
XLOC_024424


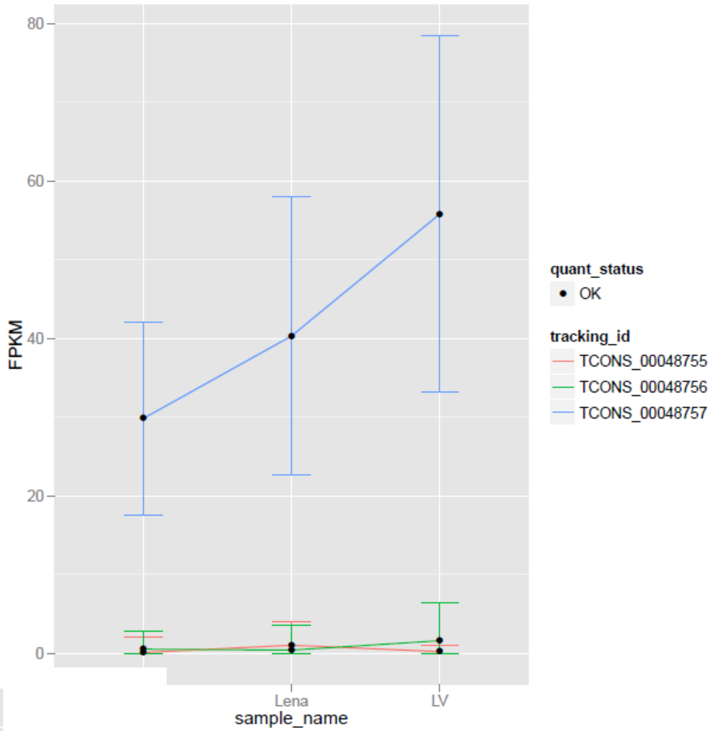

FPKM
80
60
40
20
0
Lena
LV
sample_name
quant_status
OK
tracking_id
TCONS_00048755
TCONS_00048756
TCONS_00048757


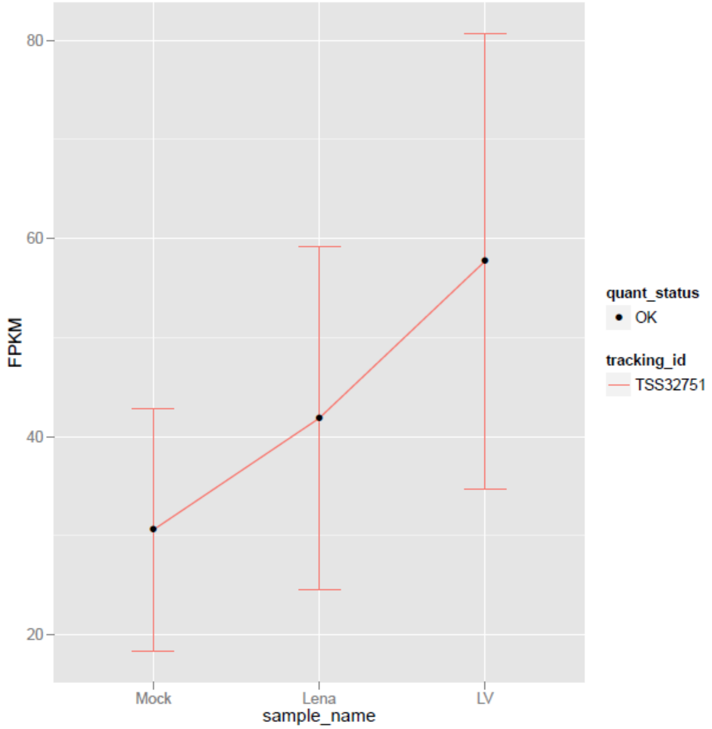

FPKM
80
60
40
20
Mock
Lena
LV
sample_name
quant_status
OK
tracking_id
TSS32751

# IRF7

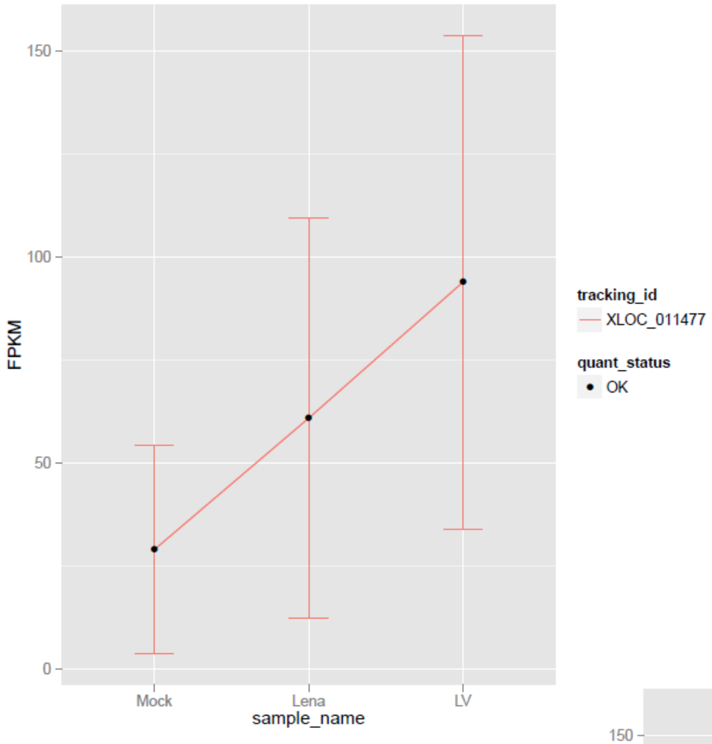

150
100
50
0
FPKM
Mock
Lena
LV
sample_name
tracking_id
XLOC_011477
quant_status
OK


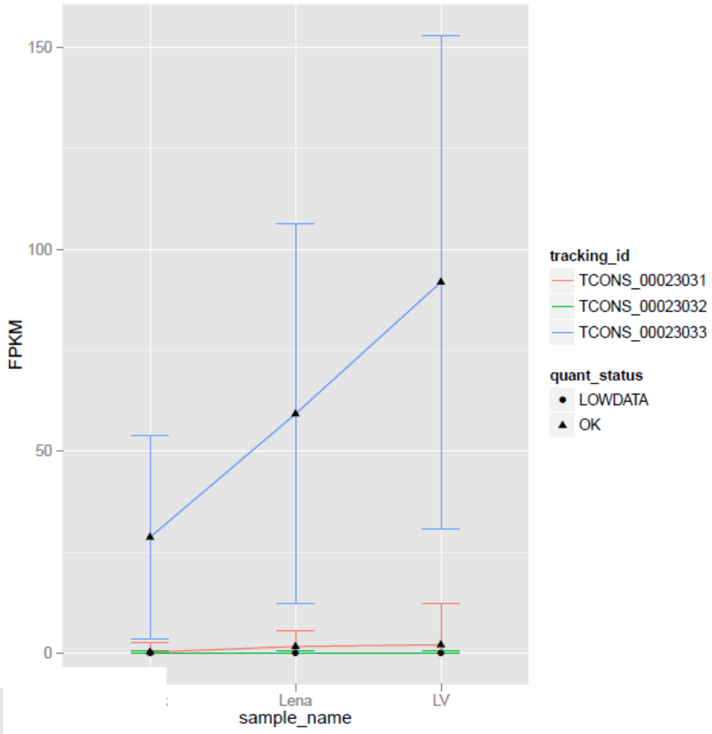

150
100
50
0
FPKM
Lena
LV
sample_name
tracking_id
TCONS_00023031
TCONS_00023032
TCONS_00023033
quant_status
LOWDATA
OK


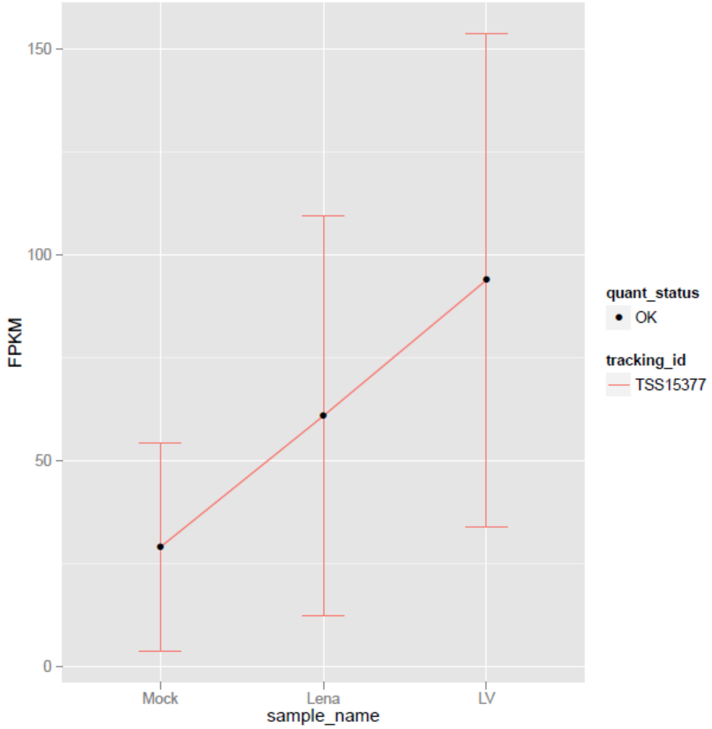

150
100
50
0
FPKM
Mock
Lena
LV
sample_name
quant_status
OK
tracking_id
TSS15377

# MICB

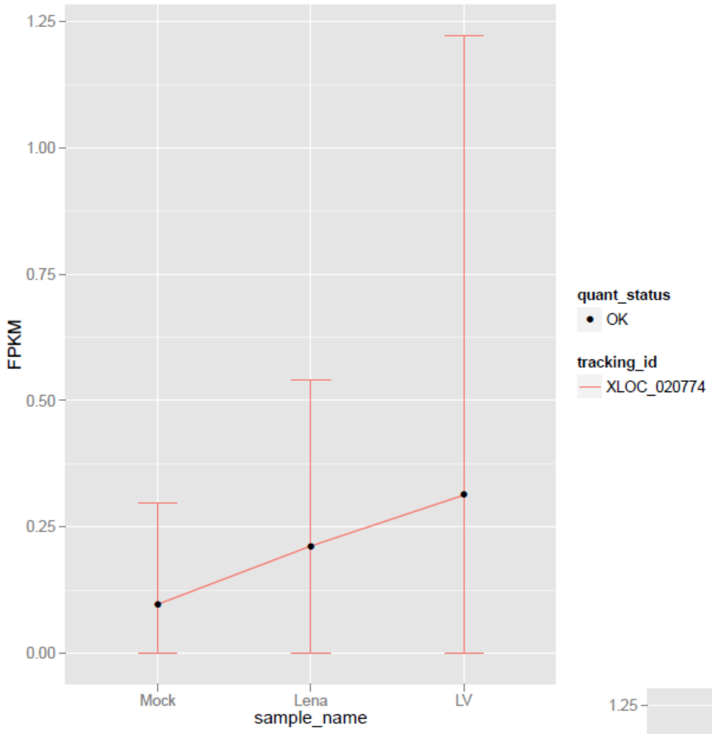

1.25
1.00
0.75
0.50
0.25
0.00
FPKM
Mock
Lena
LV
sample_name
quant_status
OK
tracking_id
XLOC_020774


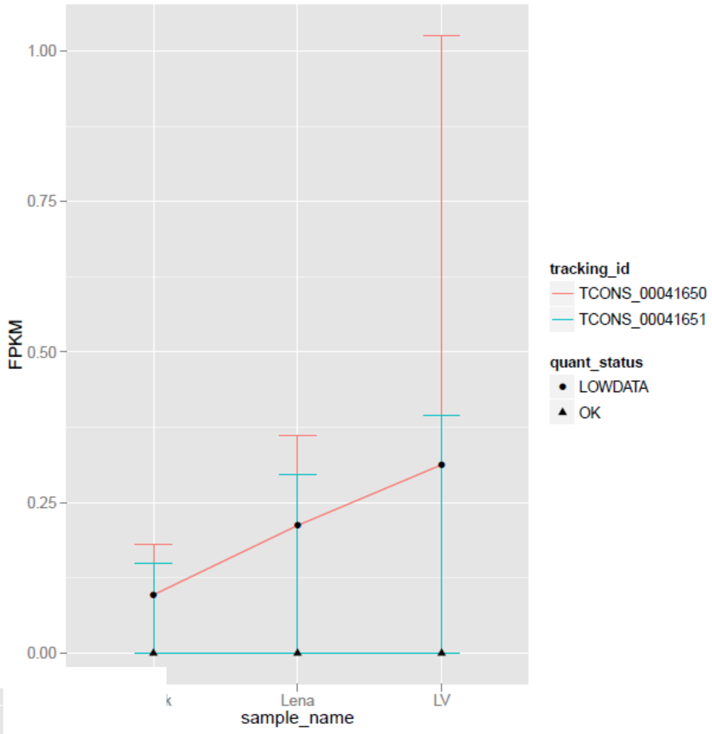

1.00
0.75
0.50
0.25
0.00
FPKM
Lena
LV
sample_name
tracking_id
TCONS_00041650
TCONS_00041651
quant_status
LOWDATA
OK


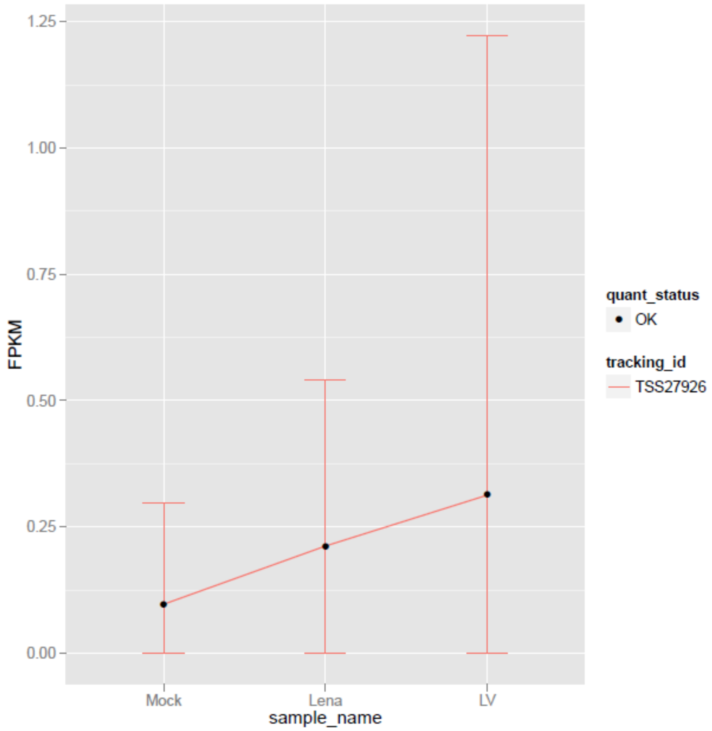

1.25
1.00
0.75
0.50
0.25
0.00
FPKM
Mock
Lena
LV
sample_name
quant_status
OK
tracking_id
TSS27926

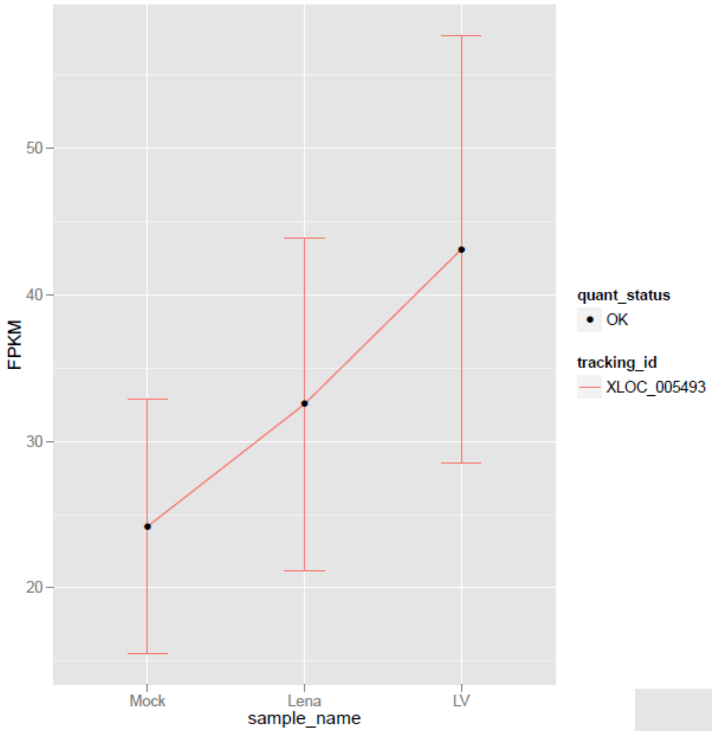
FPKM
50
40
30
20
Mock
Lena
LV
sample_name
quant_status
OK
tracking_id
XLOC_005493

# TIPARP

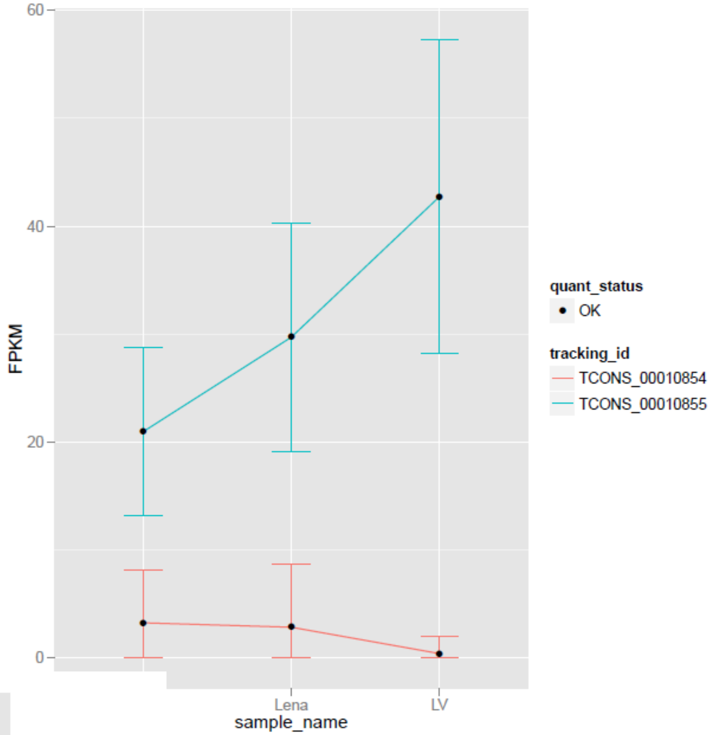
FPKM
60
40
20
0
Lena
LV
sample_name
quant_status
OK
tracking_id
TCONS_00010854
TCONS_00010855

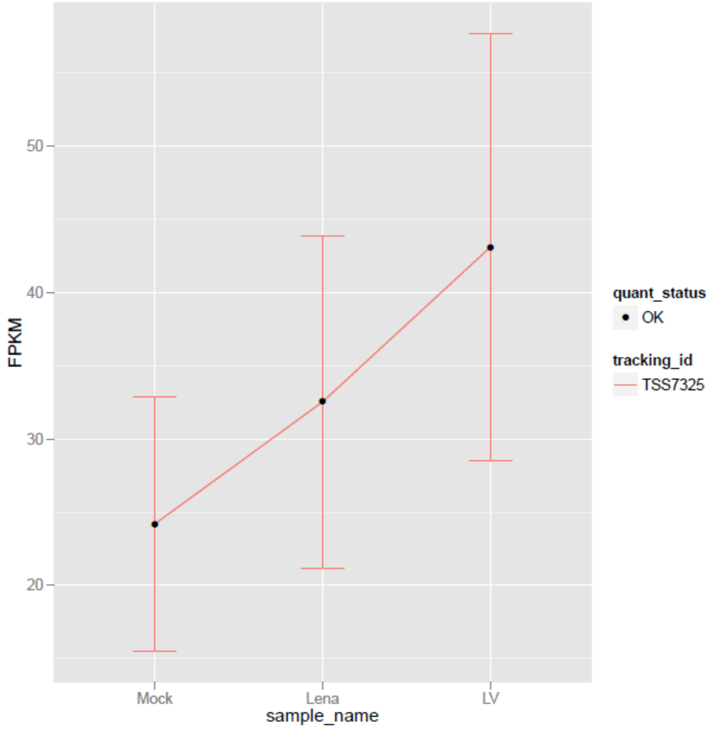
FPKM
50
40
30
20
Mock
Lena
LV
sample_name
quant_status
OK
tracking_id
TSS7325

# STAT1

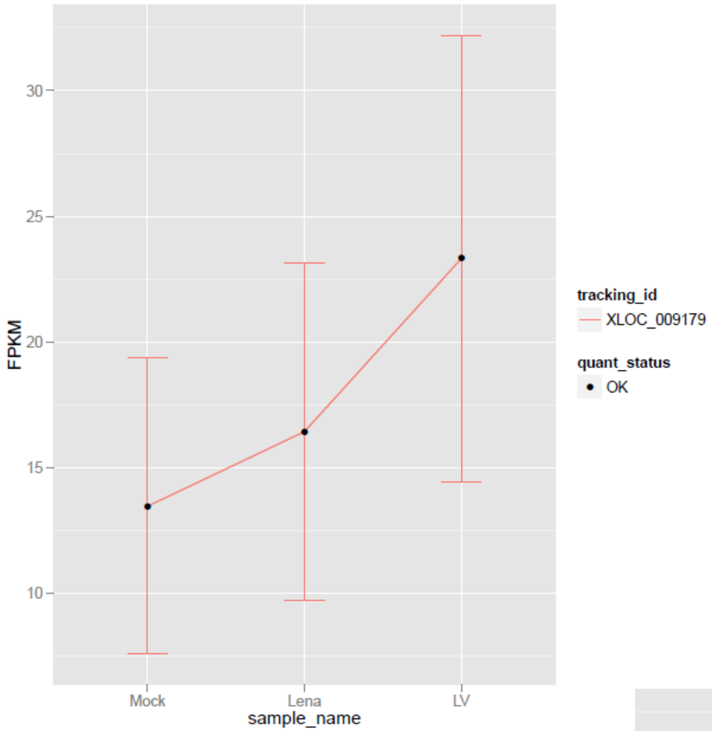

FPKM
30
25
20
15
10
Mock
Lena
LV
sample_name
tracking_id
XLOC_009179
quant_status
OK


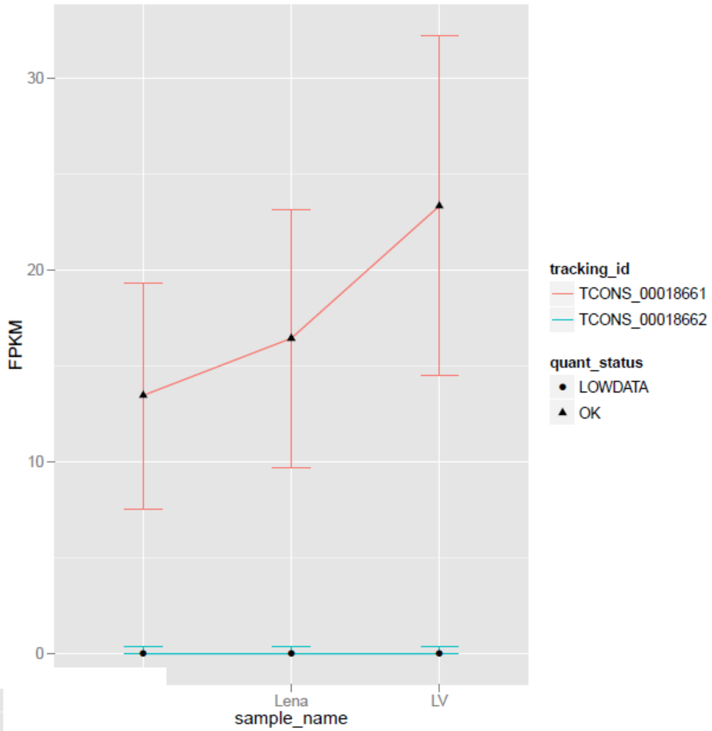

FPKM
30
20
10
0
Lena
LV
sample_name
tracking_id
TCONS_00018661
TCONS_00018662
quant_status
LOWDATA
OK


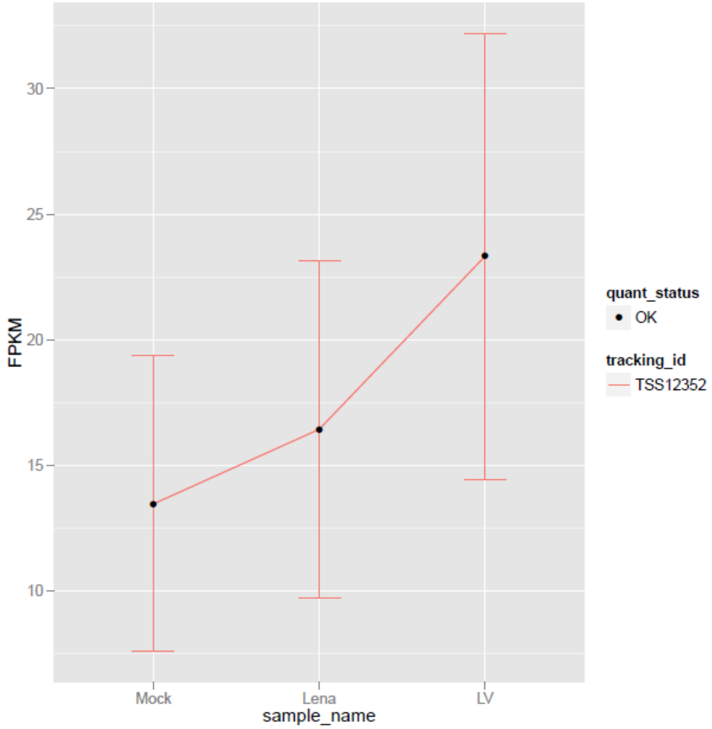

FPKM
30
25
20
15
10
Mock
Lena
LV
sample_name
quant_status
OK
tracking_id
TSS12352

# IFNB1

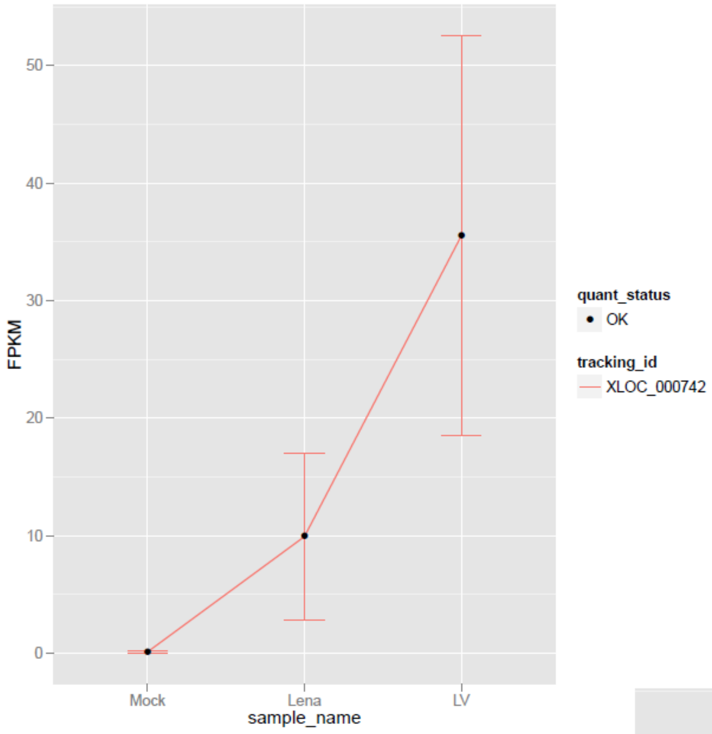

FPKM
50
40
30
20
10
0
Mock
Lena
LV
sample_name
quant_status
OK
tracking_id
XLOC_000742


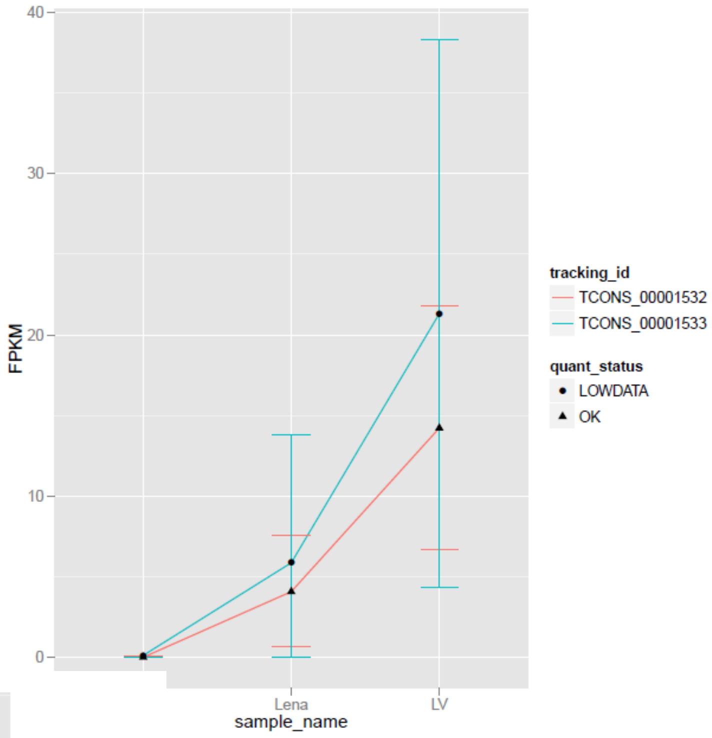

FPKM
40
30
20
10
0
Lena
LV
sample_name
tracking_id
TCONS_00001532
TCONS_00001533
quant_status
LOWDATA
OK


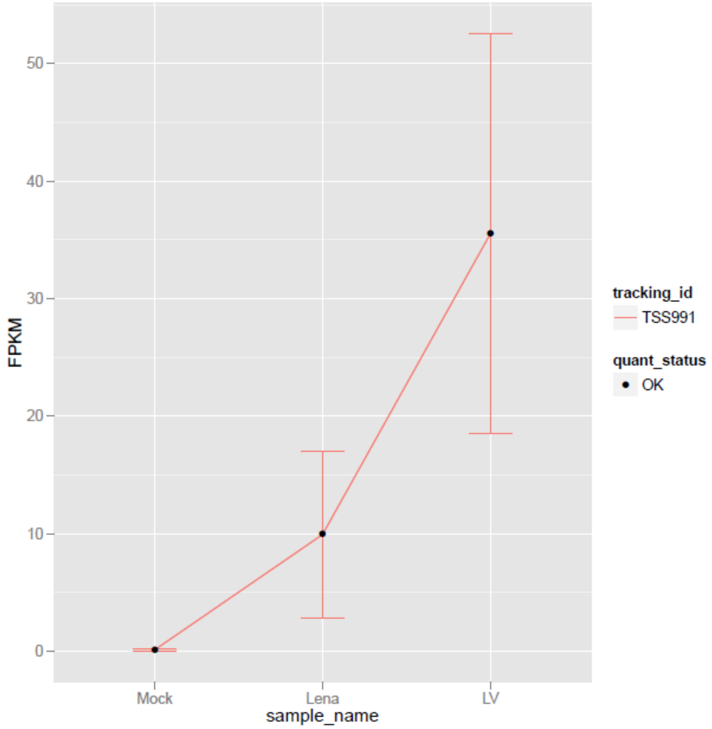

FPKM
50
40
30
20
10
0
Mock
Lena
LV
sample_name
tracking_id
TSS991
quant_status
OK

# Group 3:
# Spliced and both transcriptionally and post-transcriptionally regulated

# IFIT3

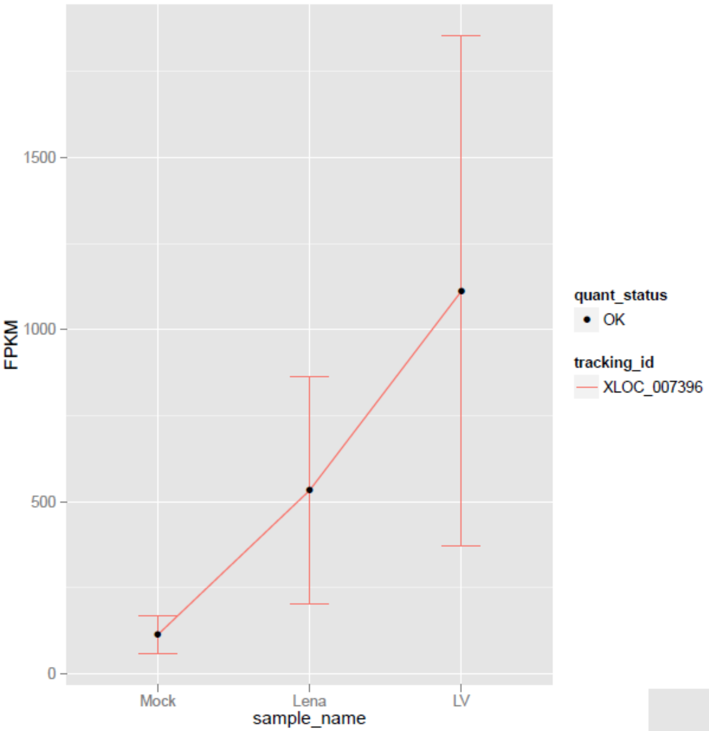

FPKM
1500
1000
500
0
Mock
Lena
LV
sample_name
quant_status
OK
tracking_id
XLOC_007396


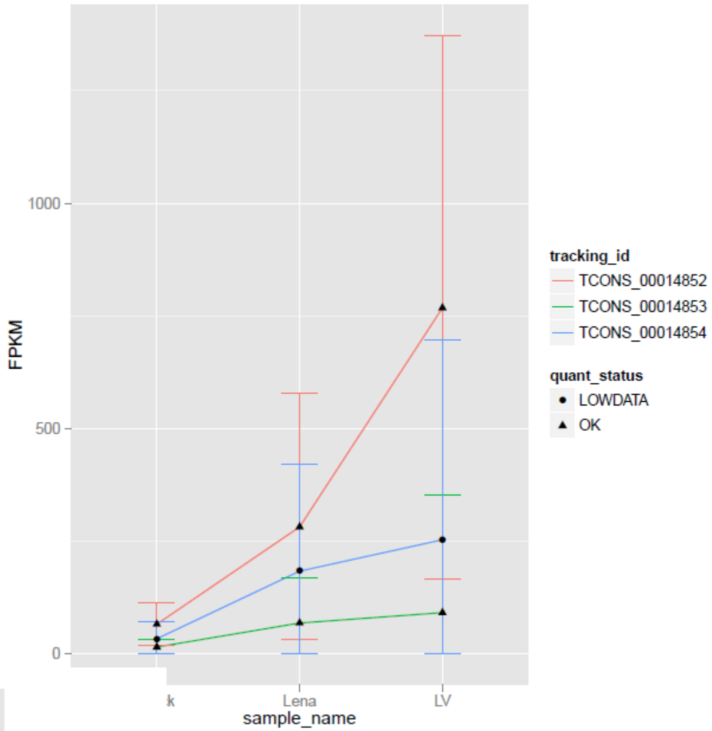

FPKM
1000
500
0
k
Lena
LV
sample_name
tracking_id
TCONS_00014852
TCONS_00014853
TCONS_00014854
quant_status
LOWDATA
OK


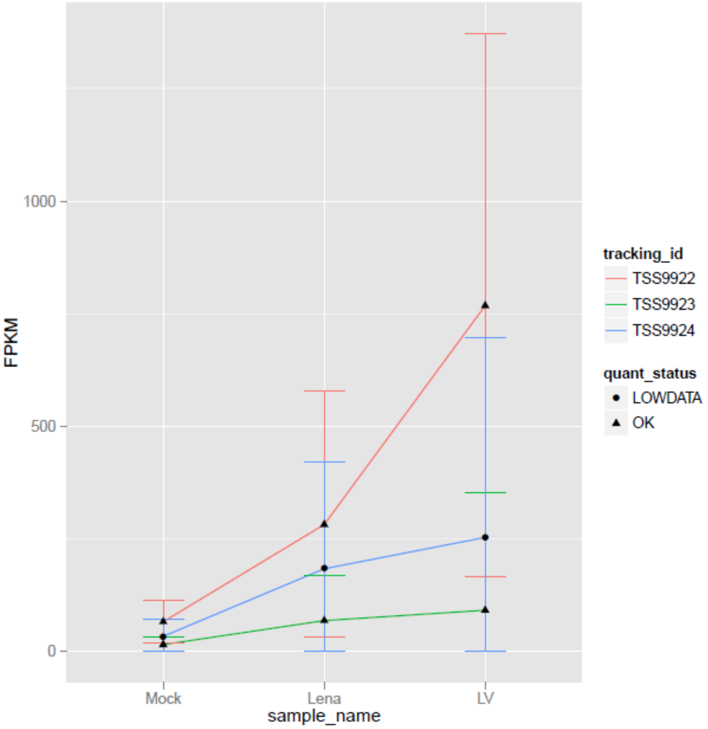

FPKM
1000
500
0
Mock
Lena
LV
sample_name
tracking_id
TSS9922
TSS9923
TSS9924
quant_status
LOWDATA
OK

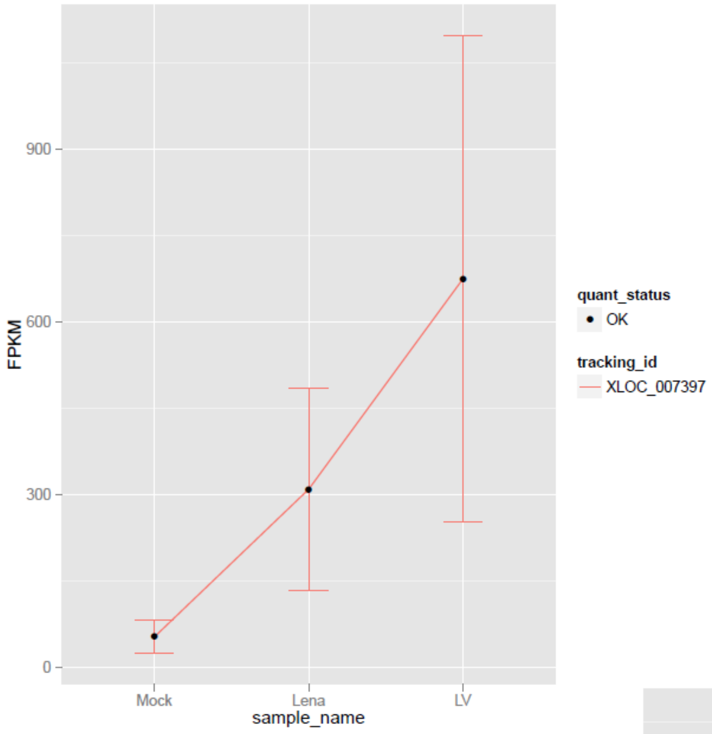

FPKM
900
600
300
0
Mock
Lena
LV
sample_name
quant_status
OK
tracking_id
XLOC_007397


# IFIT1

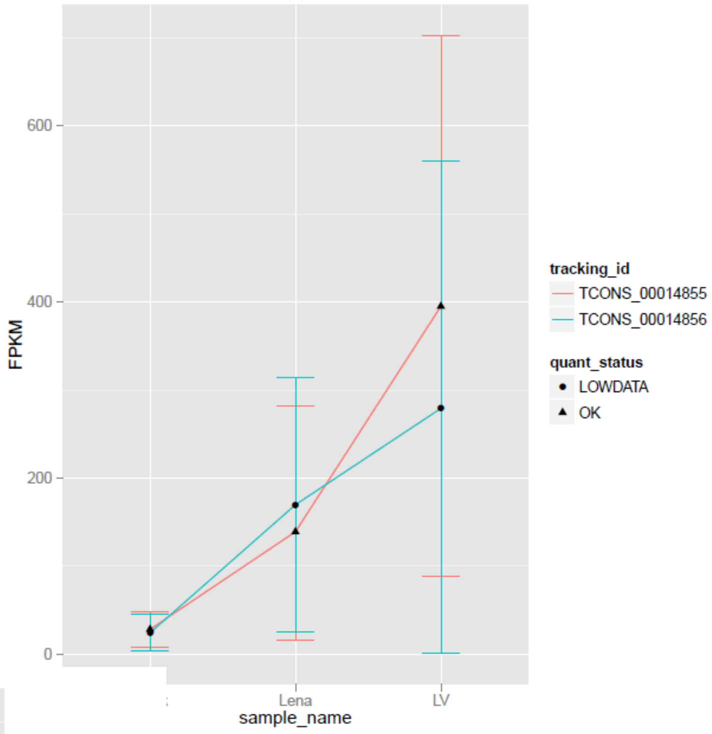

FPKM
600
400
200
0
Lena
LV
sample_name
tracking_id
TCONS_00014855
TCONS_00014856
quant_status
LOWDATA
OK


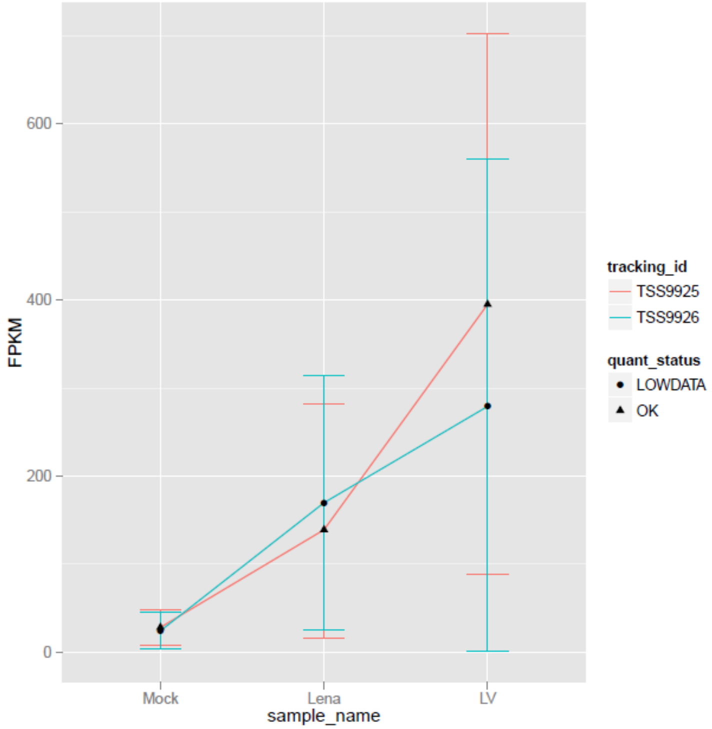

FPKM
600
400
200
0
Mock
Lena
LV
sample_name
tracking_id
TSS9925
TSS9926
quant_status
LOWDATA
OK

# OAS1

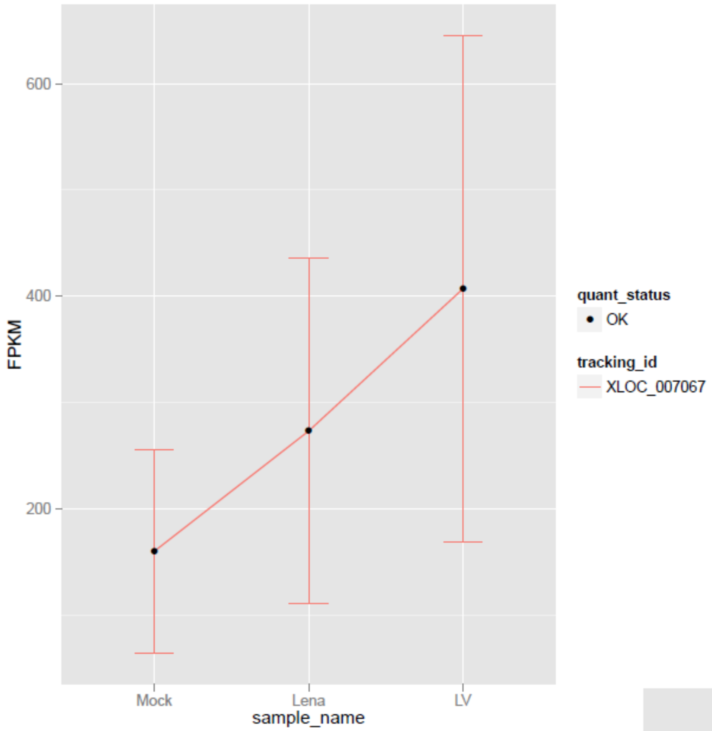

600
400
200
FPKM
Mock
Lena
LV
sample_name
quant_status
OK
tracking_id
XLOC_007067


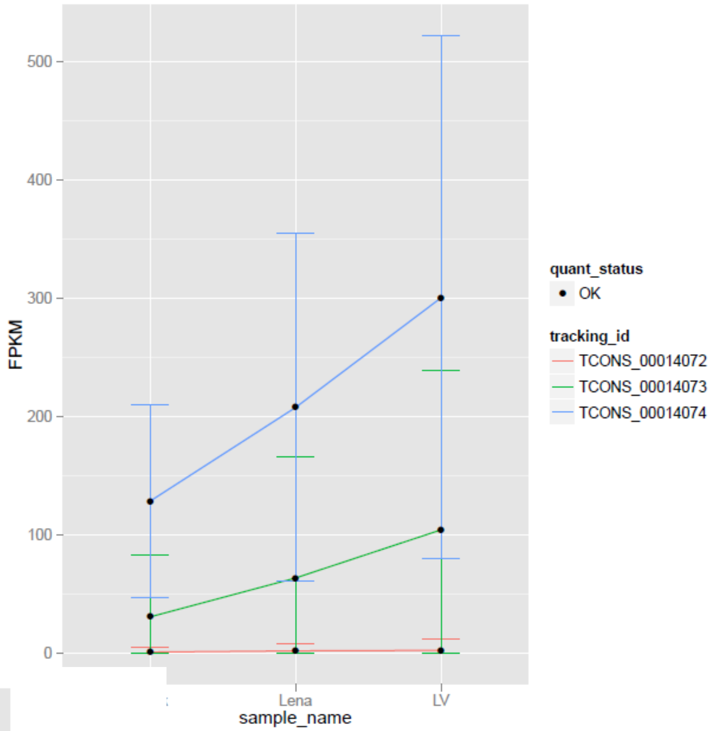

500
400
300
200
100
0
FPKM
Lena
LV
sample_name
quant_status
OK
tracking_id
TCONS_00014072
TCONS_00014073
TCONS_00014074


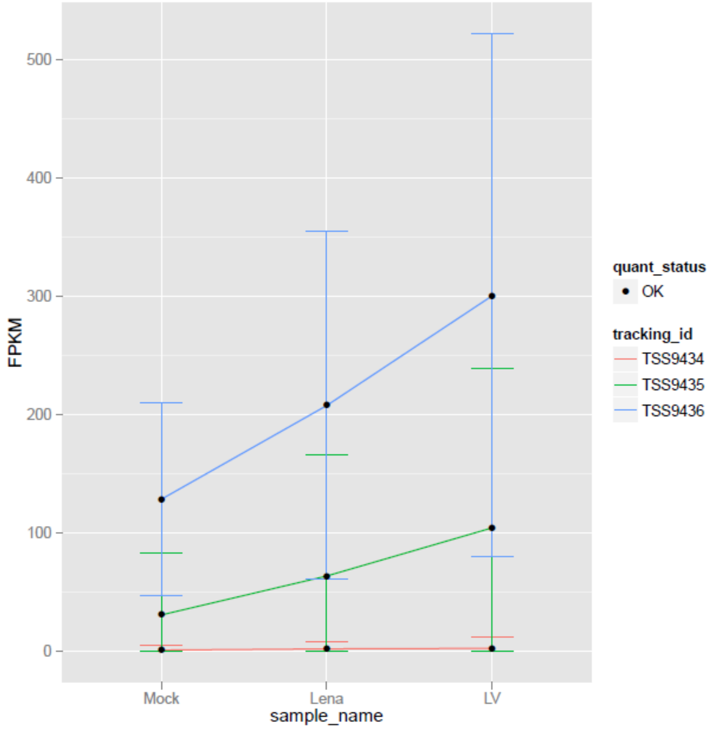

500
400
300
200
100
0
FPKM
Mock
Lena
LV
sample_name
quant_status
OK
tracking_id
TSS9434
TSS9435
TSS9436

# MX1

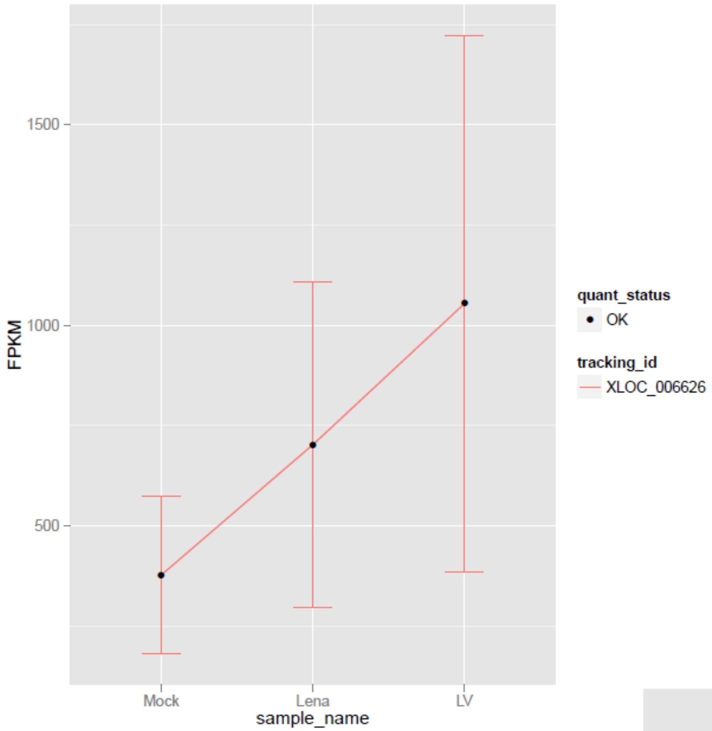
FPKM
1500
1000
500
Mock
Lena
LV
sample_name
quant_status
OK
tracking_id
XLOC_006626

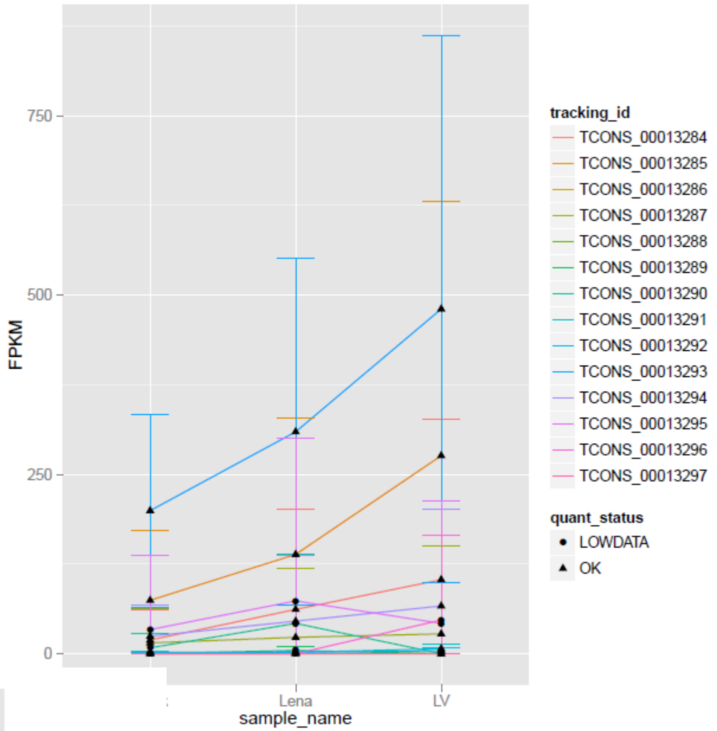
FPKM
750
500
250
0
Lena
LV
sample_name
tracking_id
TCONS_00013284
TCONS_00013285
TCONS_00013286
TCONS_00013287
TCONS_00013288
TCONS_00013289
TCONS_00013290
TCONS_00013291
TCONS_00013292
TCONS_00013293
TCONS_00013294
TCONS_00013295
TCONS_00013296
TCONS_00013297
quant_status
LOWDATA
OK

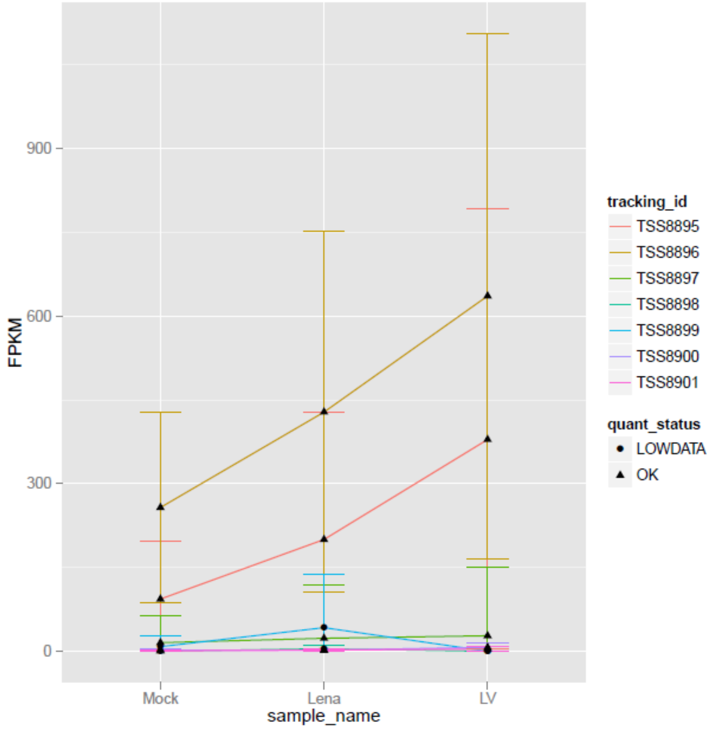
FPKM
900
600
300
0
Mock
Lena
LV
sample_name
tracking_id
TSS8895
TSS8896
TSS8897
TSS8898
TSS8899
TSS8900
TSS8901
quant_status
LOWDATA
OK

# IFNB1

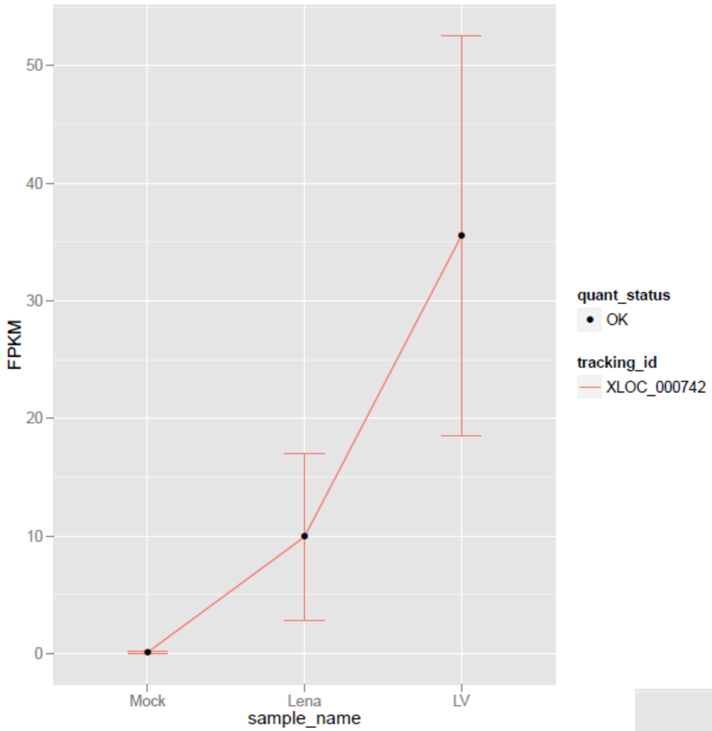
FPKM
50
40
30
20
10
0
Mock
Lena
LV
sample_name
quant_status
OK
tracking_id
XLOC_000742

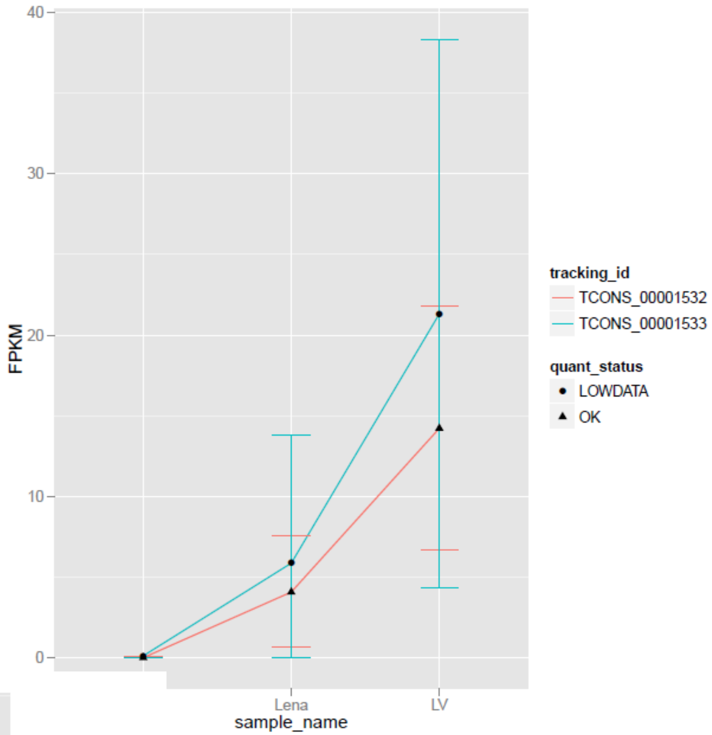
FPKM
40
30
20
10
0
Lena
LV
sample_name
tracking_id
TCONS_00001532
TCONS_00001533
quant_status
LOWDATA
OK

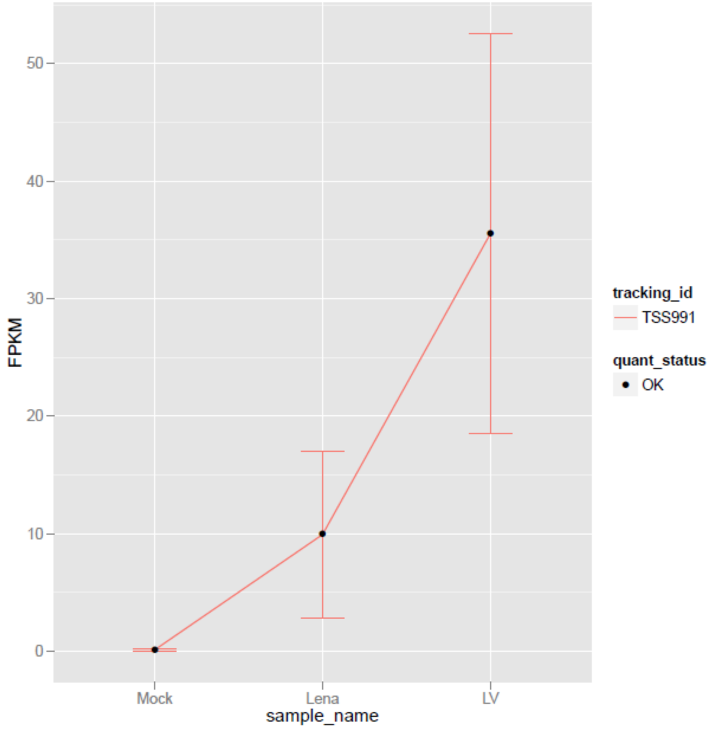
FPKM
50
40
30
20
10
0
Mock
Lena
LV
sample_name
tracking_id
TSS991
quant_status
OK

# IFITM1

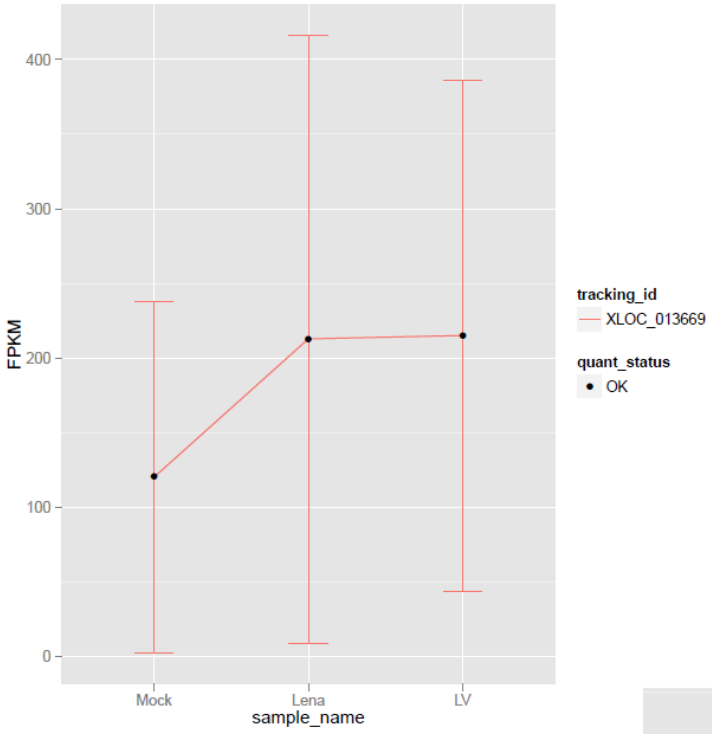

FPKM
400
300
200
100
0
Mock
Lena
LV
sample_name
tracking_id
XLOC_013669
quant_status
OK


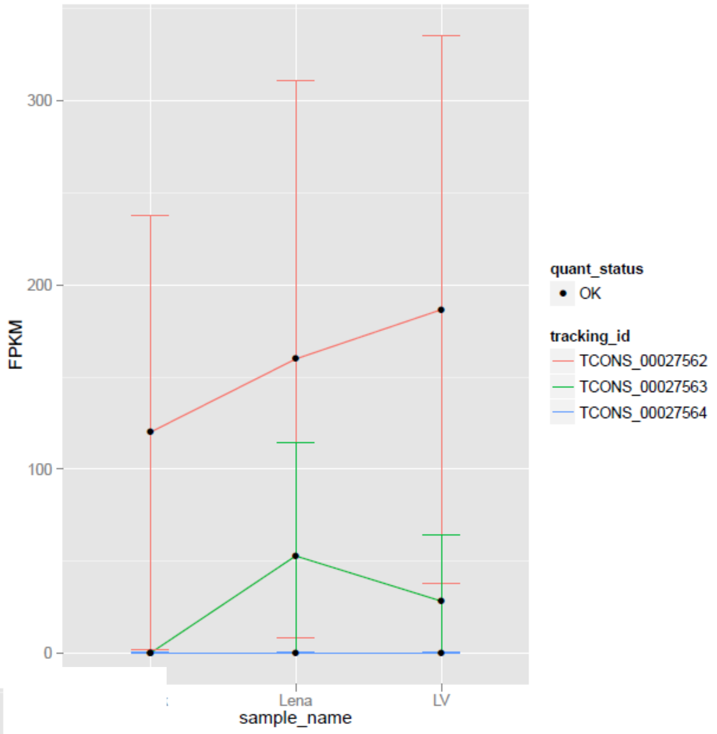

FPKM
300
200
100
0
Lena
LV
sample_name
quant_status
OK
tracking_id
TCONS_00027562
TCONS_00027563
TCONS_00027564


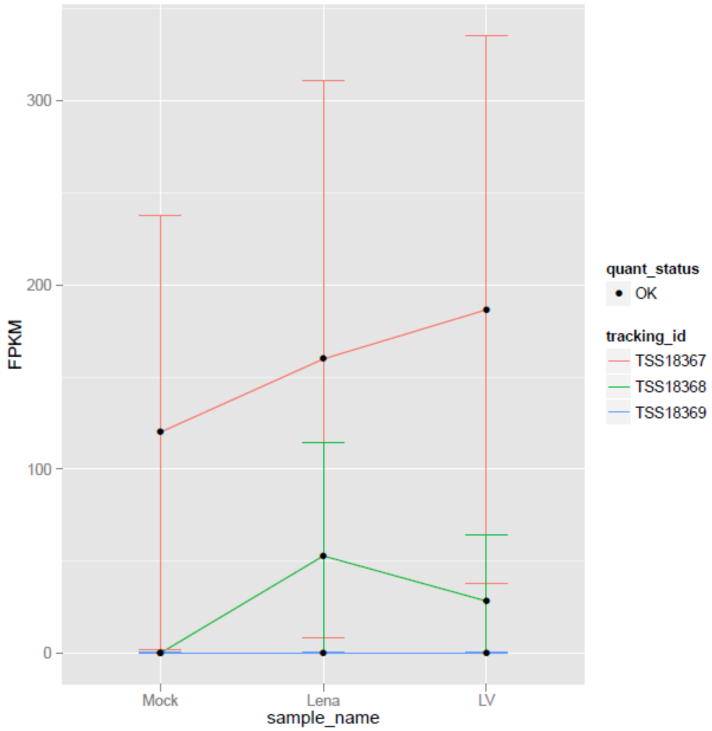

FPKM
300
200
100
0
Mock
Lena
LV
sample_name
quant_status
OK
tracking_id
TSS18367
TSS18368
TSS18369

# PSMB8

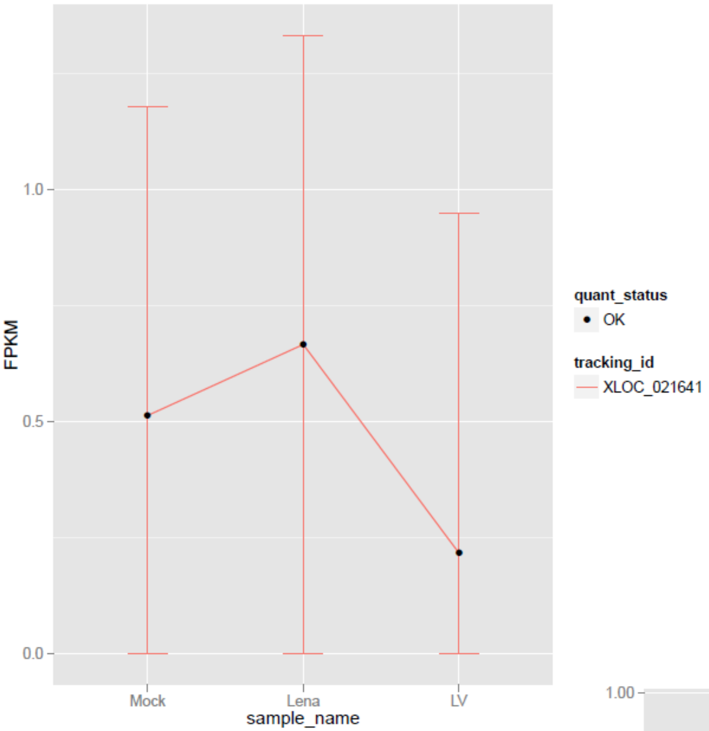
FPKM
1.0
0.5
0.0
Mock
Lena
LV
sample_name
quant_status
OK
tracking_id
XLOC_021641

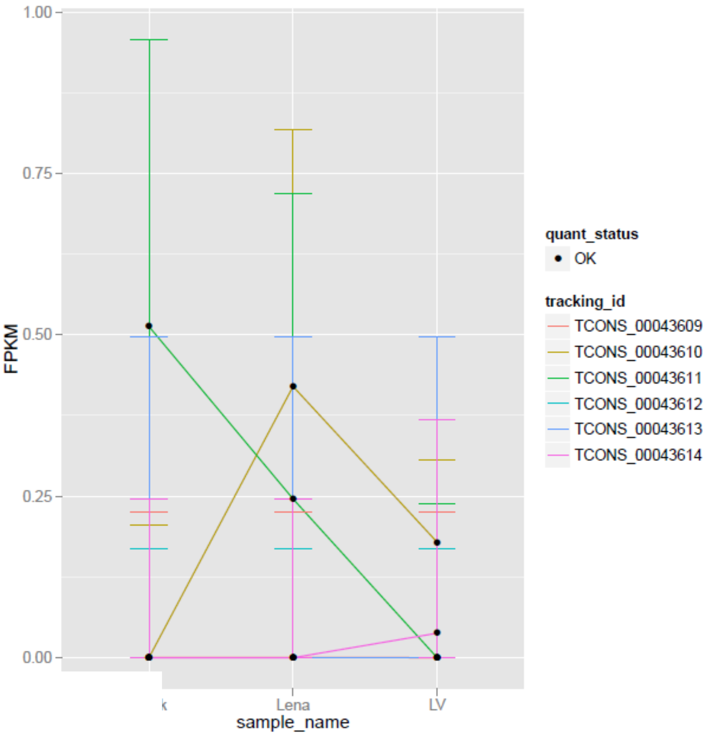
FPKM
1.00
0.75
0.50
0.25
0.00
Lena
LV
sample_name
quant_status
OK
tracking_id
TCONS_00043609
TCONS_00043610
TCONS_00043611
TCONS_00043612
TCONS_00043613
TCONS_00043614

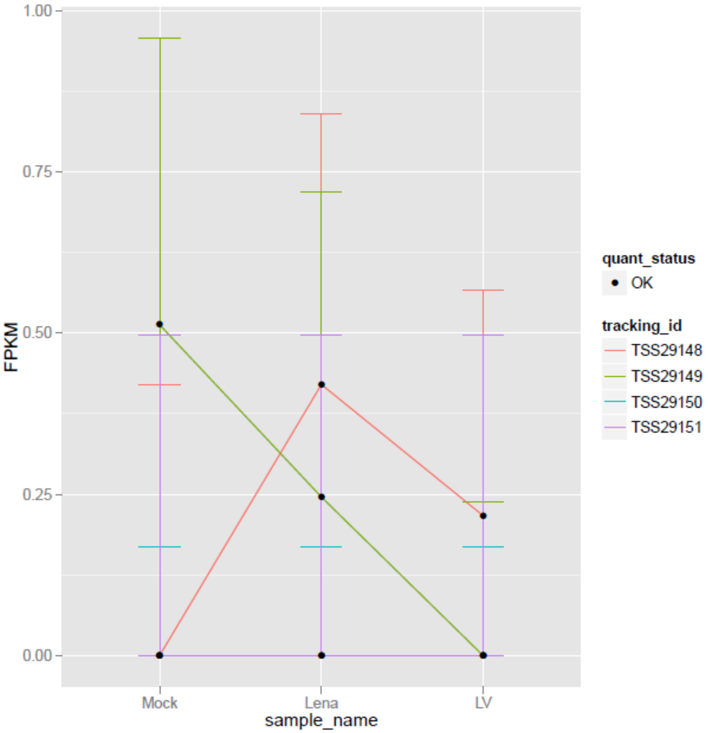
FPKM
1.00
0.75
0.50
0.25
0.00
Mock
Lena
LV
sample_name
quant_status
OK
tracking_id
TSS29148
TSS29149
TSS29150
TSS29151

# JAK2

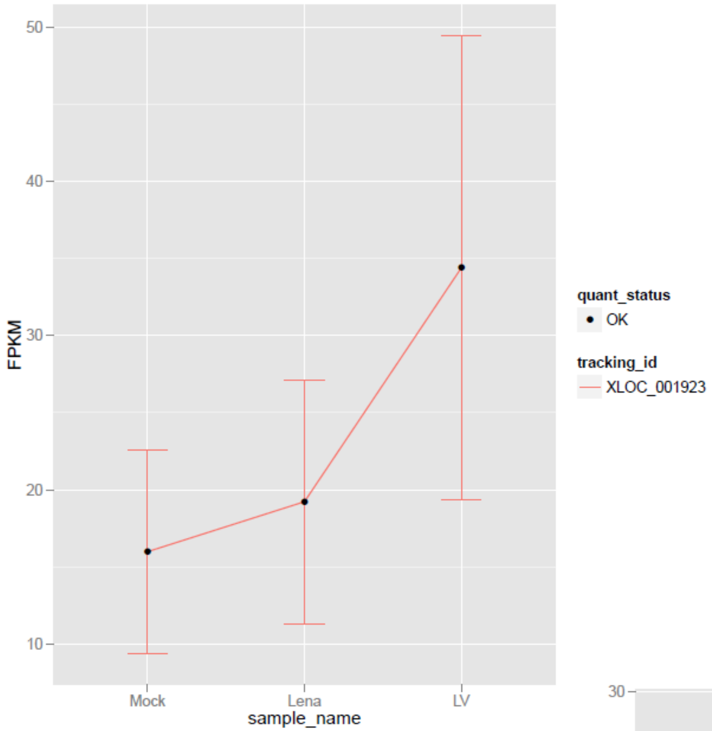
FPKM
50
40
30
20
10
Mock
Lena
LV
sample_name
quant_status
OK
tracking_id
XLOC_001923

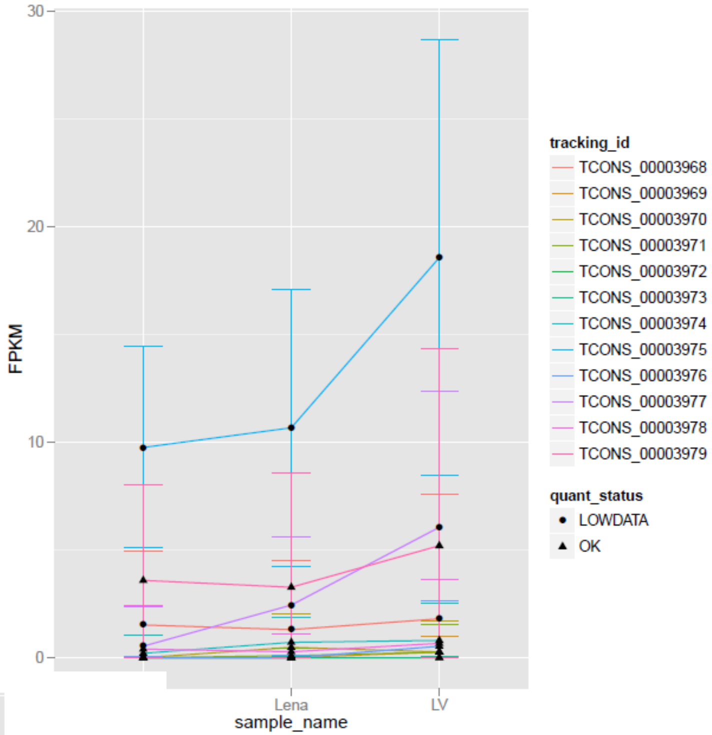
FPKM
30
20
10
0
Lena
LV
sample_name
tracking_id
TCONS_00003968
TCONS_00003969
TCONS_00003970
TCONS_00003971
TCONS_00003972
TCONS_00003973
TCONS_00003974
TCONS_00003975
TCONS_00003976
TCONS_00003977
TCONS_00003978
TCONS_00003979
quant_status
LOWDATA
OK

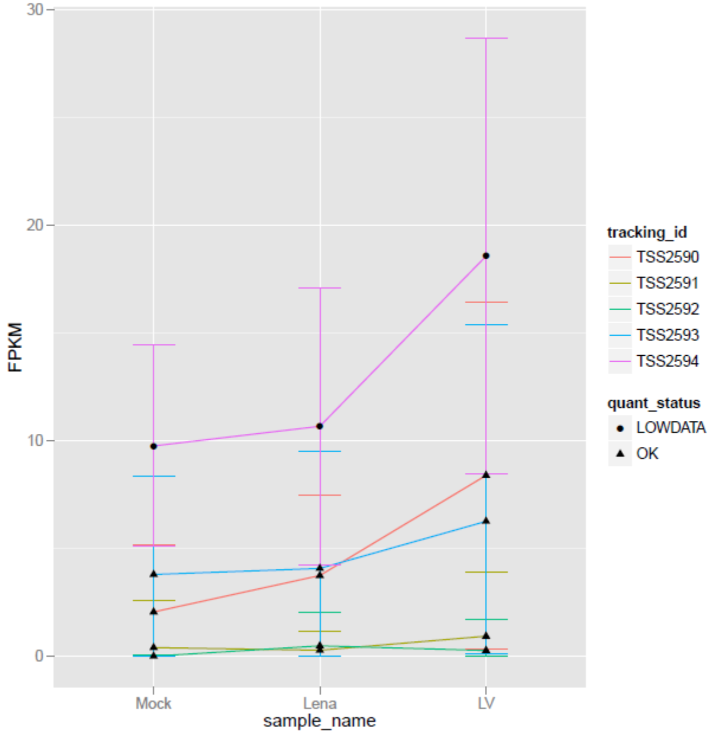
FPKM
30
20
10
0
Mock
Lena
LV
sample_name
tracking_id
TSS2590
TSS2591
TSS2592
TSS2593
TSS2594
quant_status
LOWDATA
OK

# IRF1

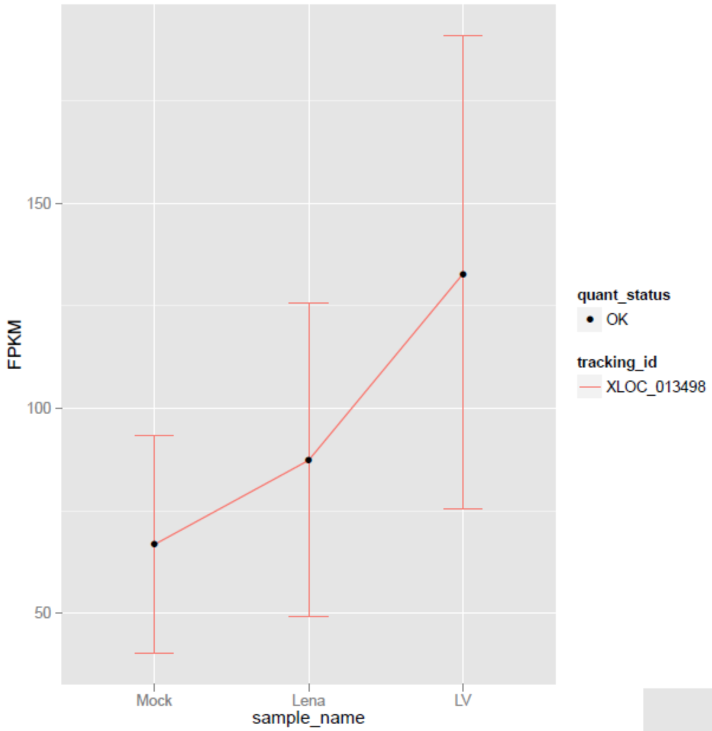

FPKM
150
100
50
Mock
Lena
LV
sample_name
quant_status
OK
tracking_id
XLOC_013498


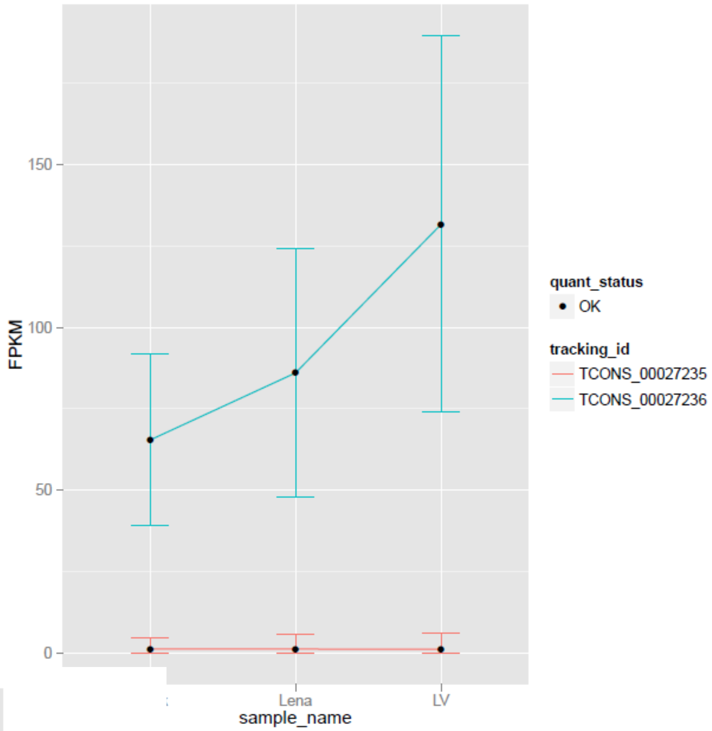

FPKM
150
100
50
0
Lena
LV
sample_name
quant_status
OK
tracking_id
TCONS_00027235
TCONS_00027236


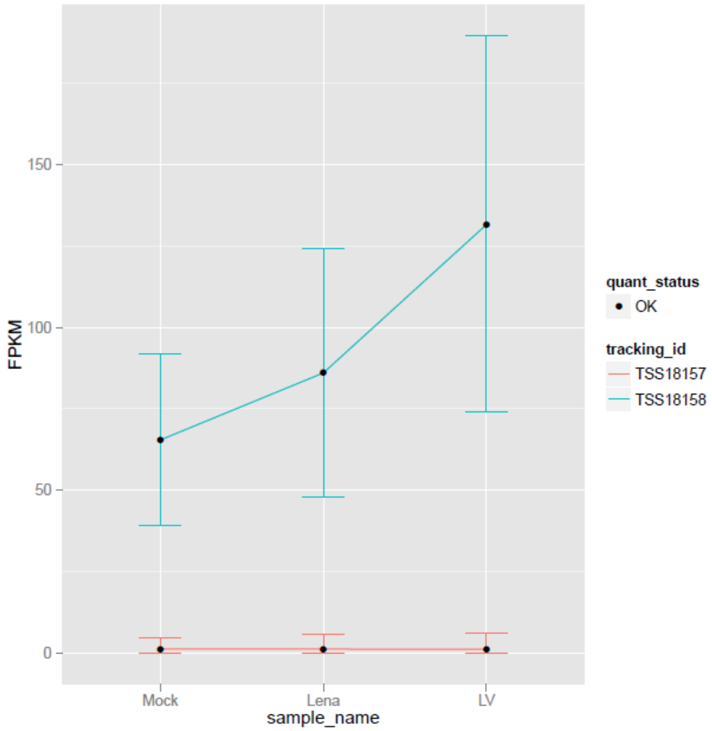

FPKM
150
100
50
0
Mock
Lena
LV
sample_name
quant_status
OK
tracking_id
TSS18157
TSS18158

# DHX58

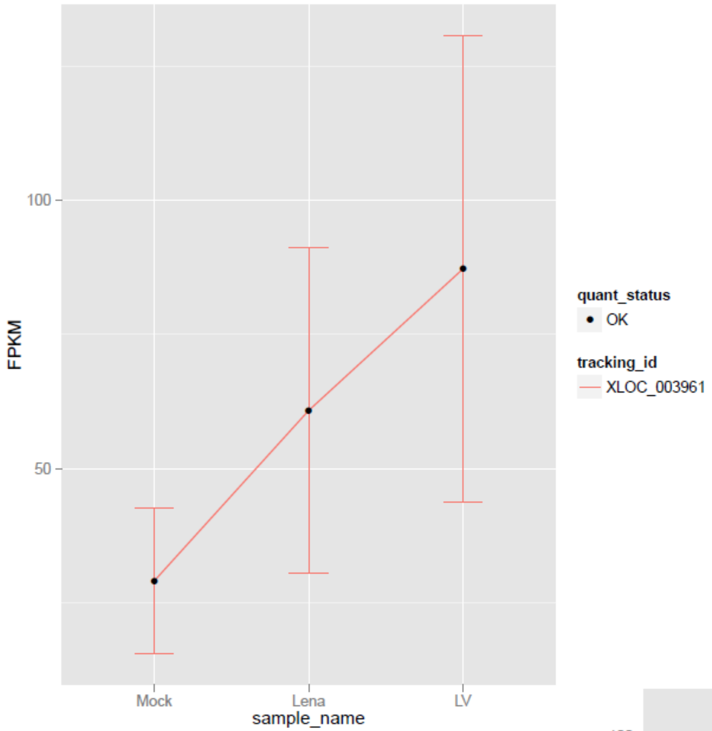

100
50
FPKM
Mock
Lena
LV
sample_name
quant_status
OK
tracking_id
XLOC_003961


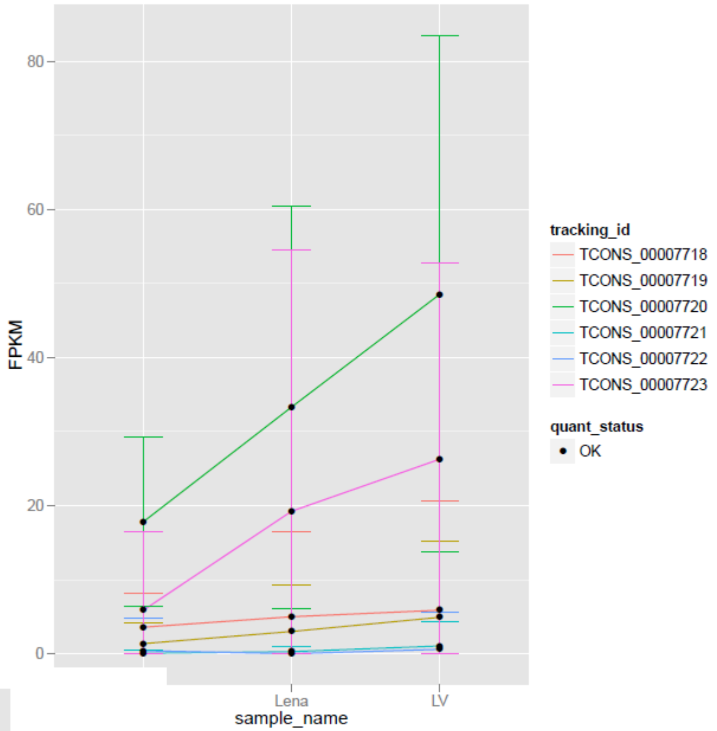

80
60
40
20
0
FPKM
Lena
LV
sample_name
tracking_id
TCONS_00007718
TCONS_00007719
TCONS_00007720
TCONS_00007721
TCONS_00007722
TCONS_00007723
quant_status
OK


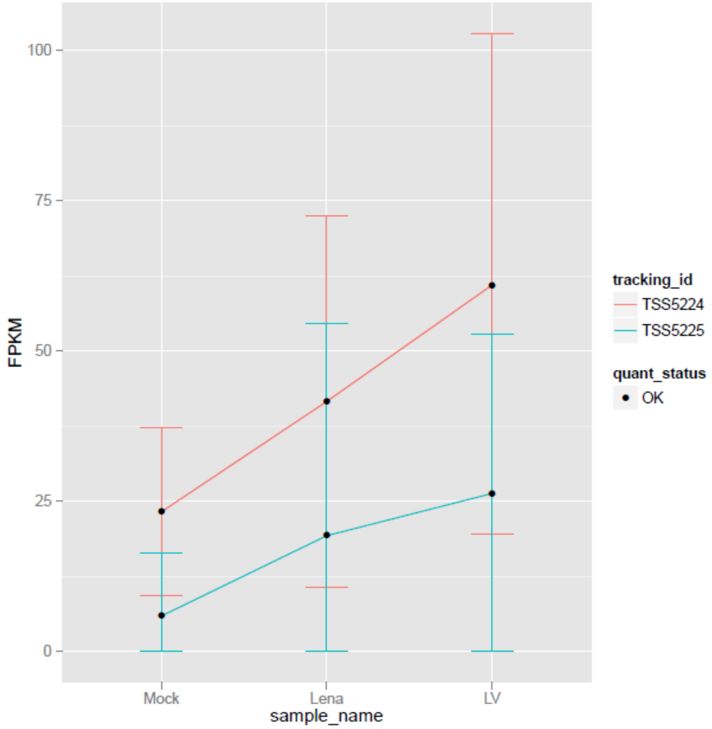

100
75
50
25
0
FPKM
Mock
Lena
LV
sample_name
tracking_id
TSS5224
TSS5225
quant_status
OK

# IL10

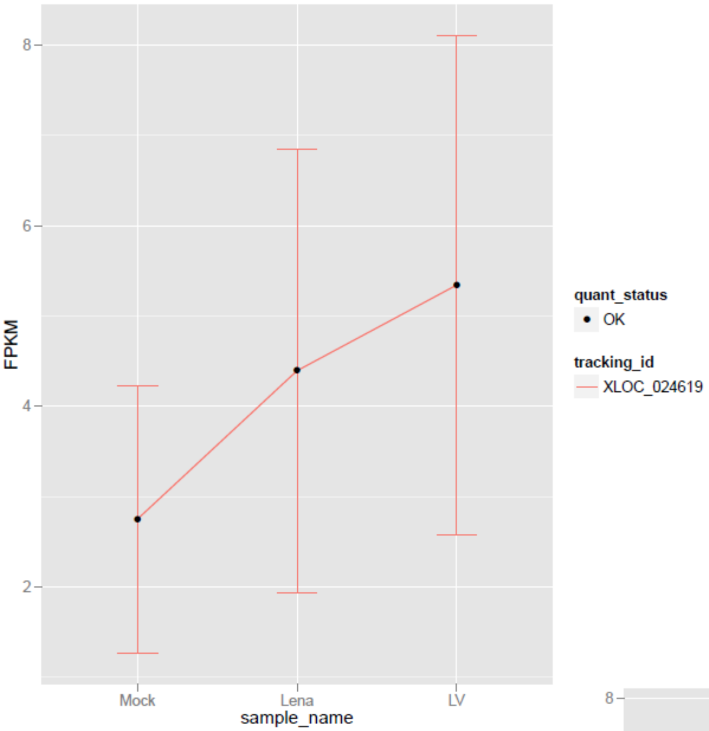

FPKM
8
6
4
2
Mock
Lena
LV
sample_name
quant_status
OK
tracking_id
XLOC_024619


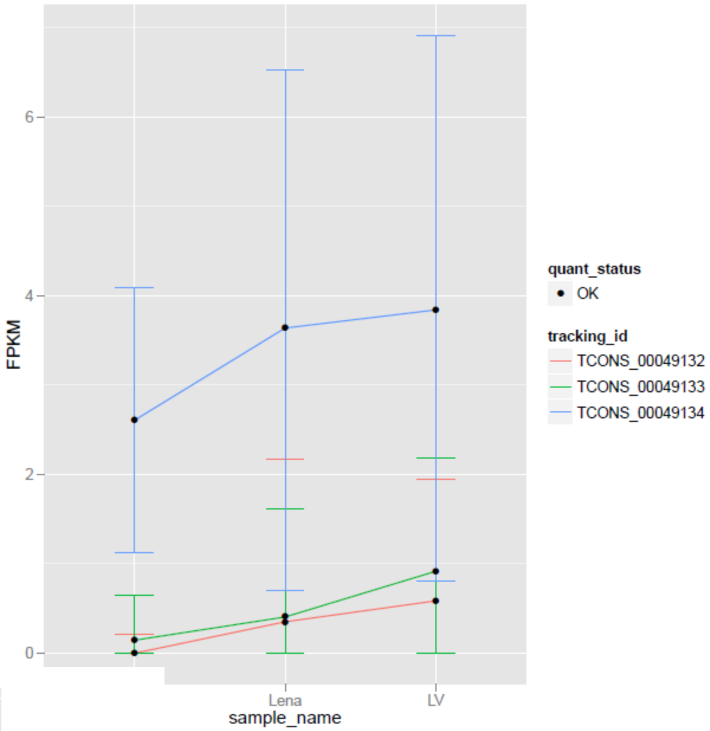

FPKM
6
4
2
0
Lena
LV
sample_name
quant_status
OK
tracking_id
TCONS_00049132
TCONS_00049133
TCONS_00049134


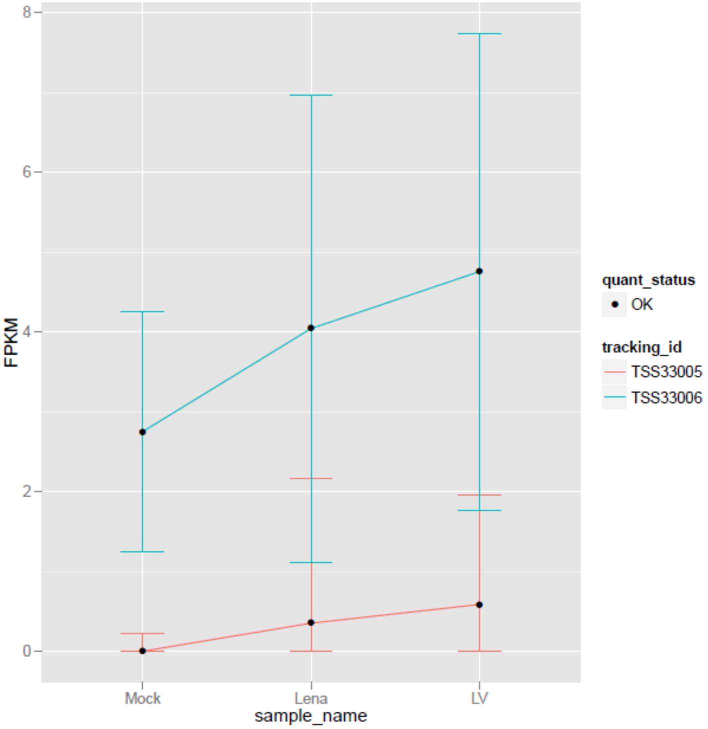

FPKM
8
6
4
2
0
Mock
Lena
LV
sample_name
quant_status
OK
tracking_id
TSS33005
TSS33006

# ZBP1

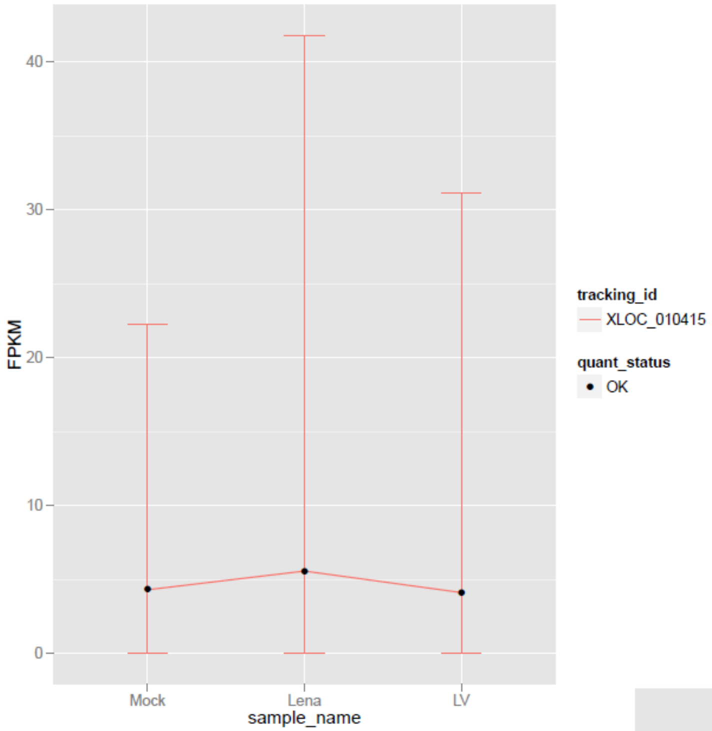
FPKM
0
10
20
30
40
Mock
Lena
LV
sample_name
tracking_id
XLOC_010415
quant_status
OK

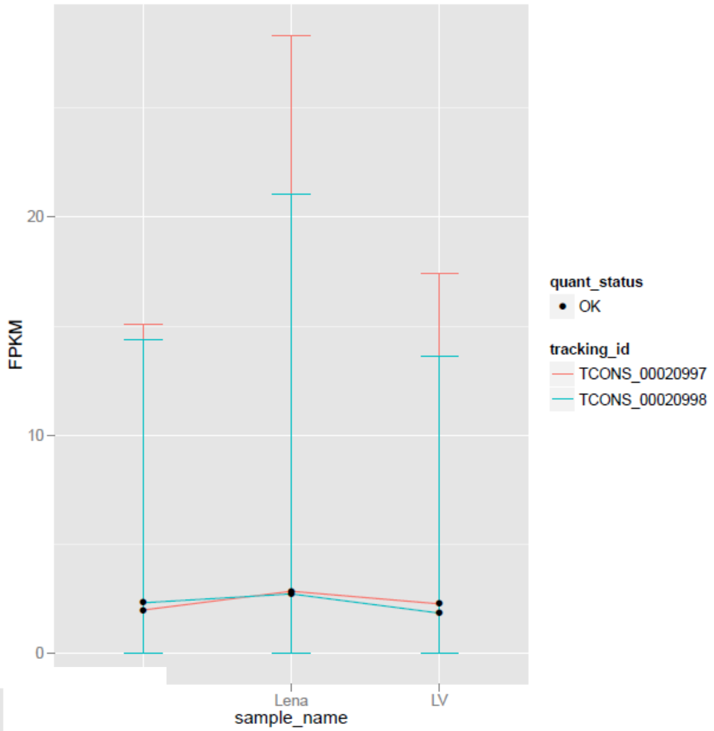
FPKM
0
10
20
Lena
LV
sample_name
quant_status
OK
tracking_id
TCONS_00020997
TCONS_00020998

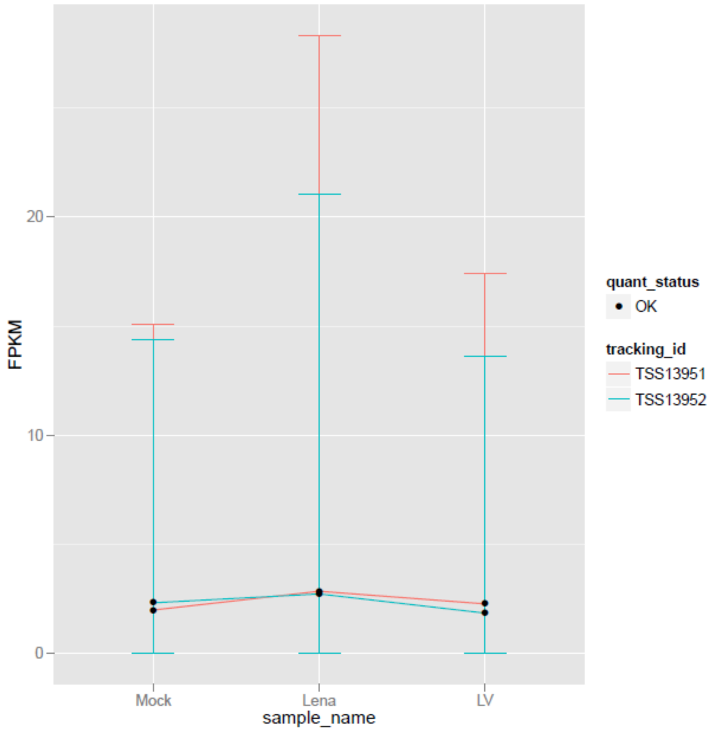
FPKM
0
10
20
Mock
Lena
LV
sample_name
quant_status
OK
tracking_id
TSS13951
TSS13952

# ADAR

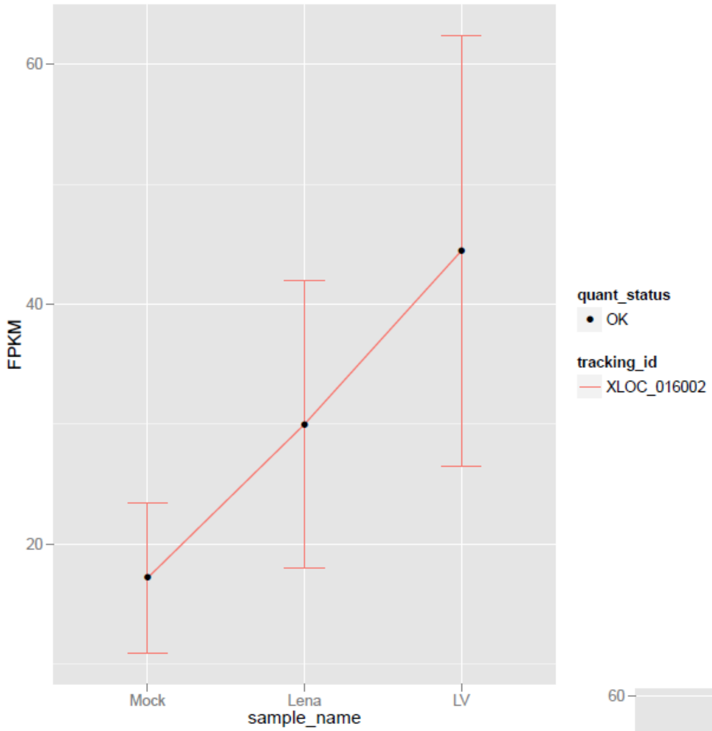

60
40
20
FPKM
Mock
Lena
LV
sample_name
quant_status
OK
tracking_id
XLOC_016002


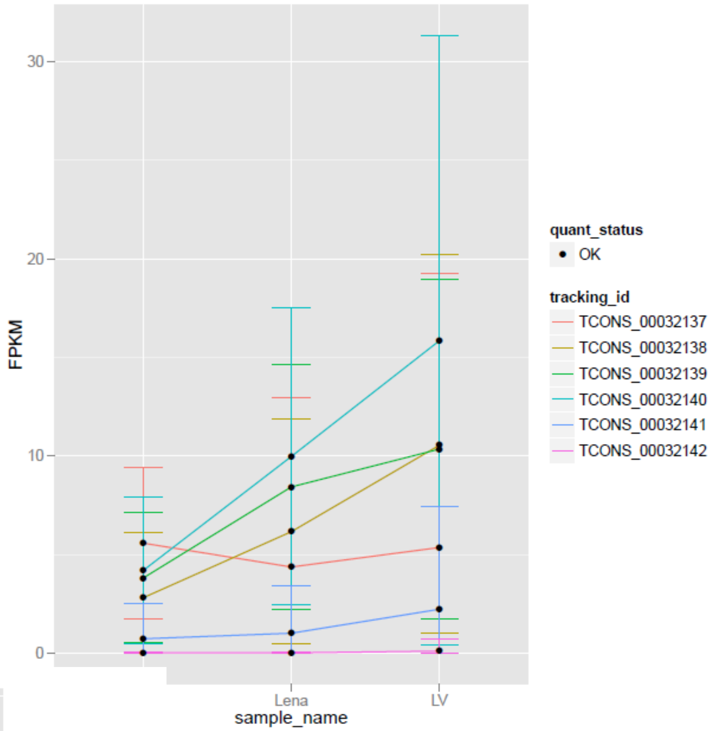

30
20
10
0
FPKM
Lena
LV
sample_name
quant_status
OK
tracking_id
TCONS_00032137
TCONS_00032138
TCONS_00032139
TCONS_00032140
TCONS_00032141
TCONS_00032142


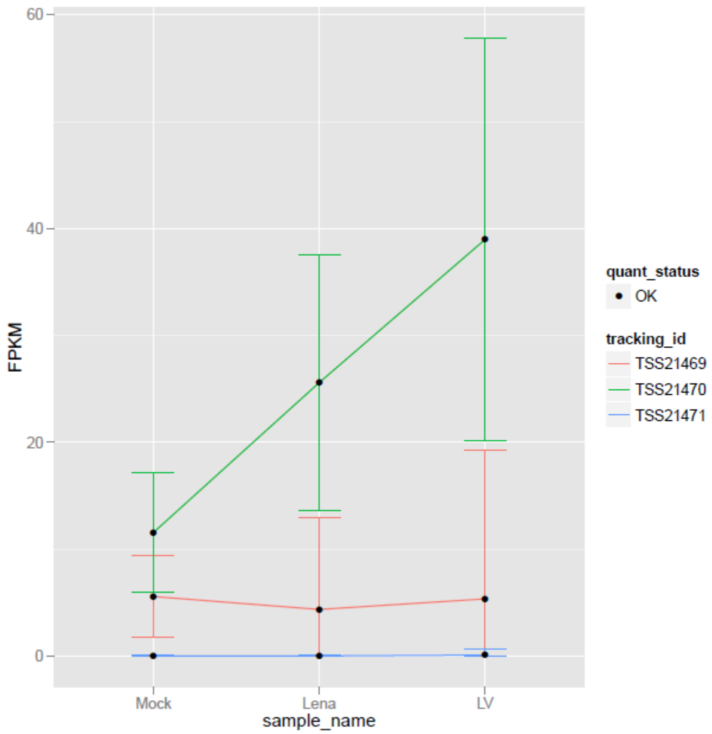

60
40
20
0
FPKM
Mock
Lena
LV
sample_name
quant_status
OK
tracking_id
TSS21469
TSS21470
TSS21471

# ISG15

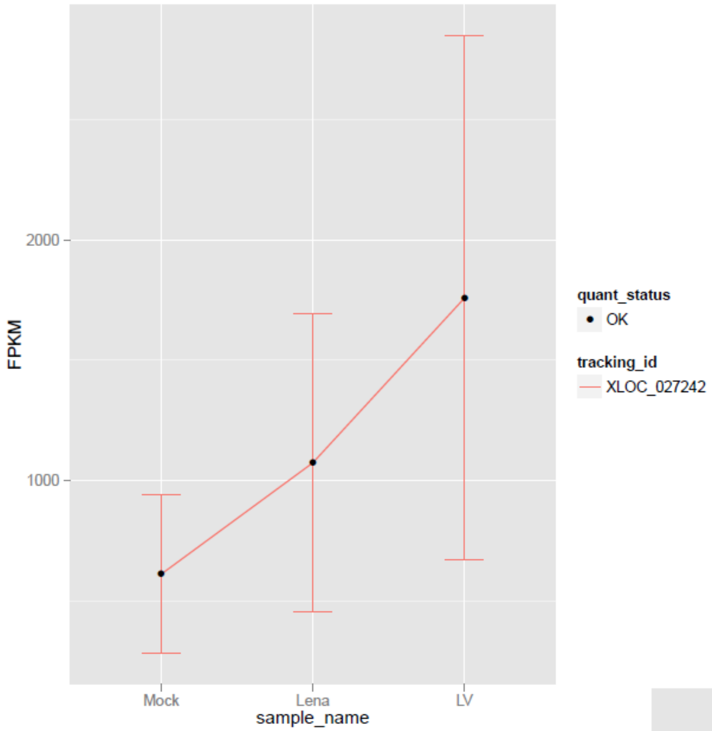

FPKM
2000
1000
Mock
Lena
LV
sample_name
quant_status
OK
tracking_id
XLOC_027242


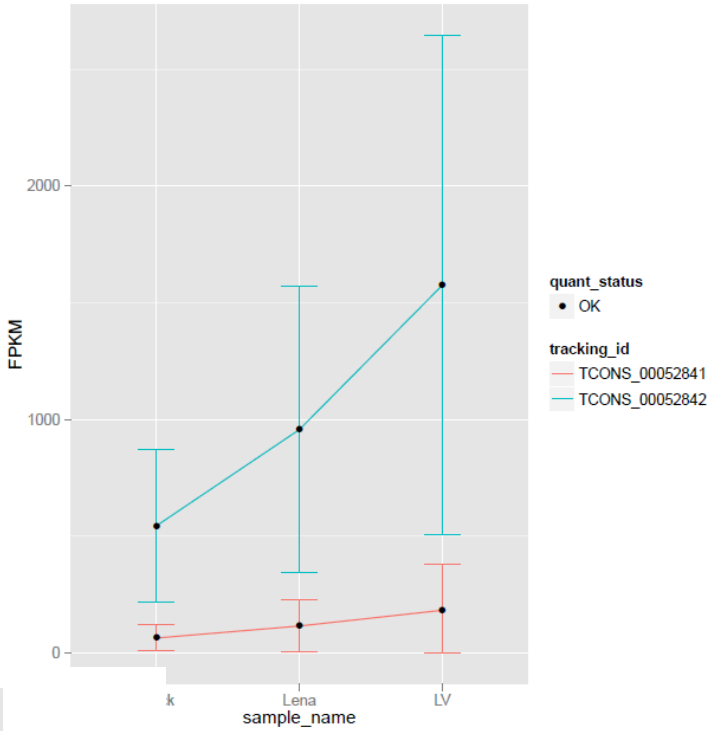

FPKM
2000
1000
0
Lena
LV
sample_name
quant_status
OK
tracking_id
TCONS_00052841
TCONS_00052842


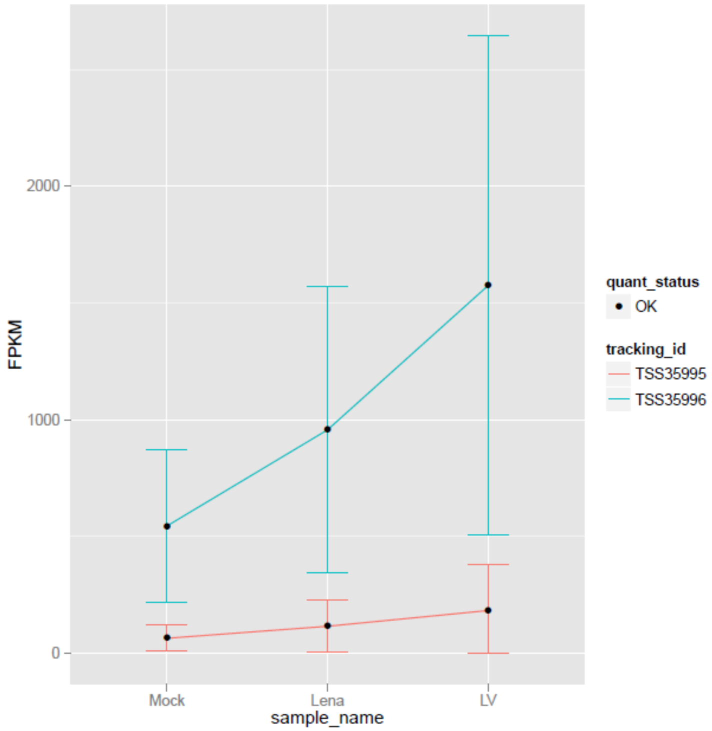

FPKM
2000
1000
0
Mock
Lena
LV
sample_name
quant_status
OK
tracking_id
TSS35995
TSS35996

# IFIH1

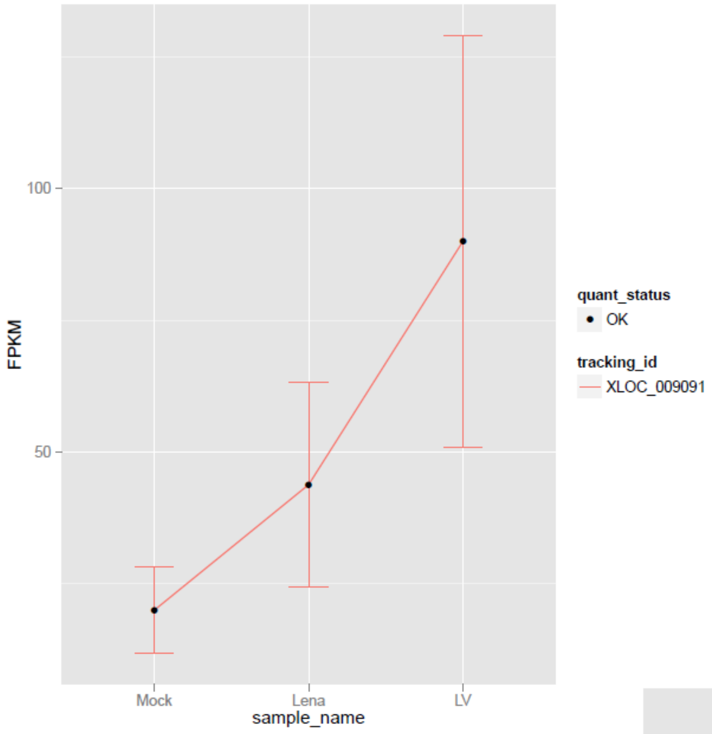

FPKM
100
50
Mock
Lena
LV
sample_name
quant_status
OK
tracking_id
XLOC_009091


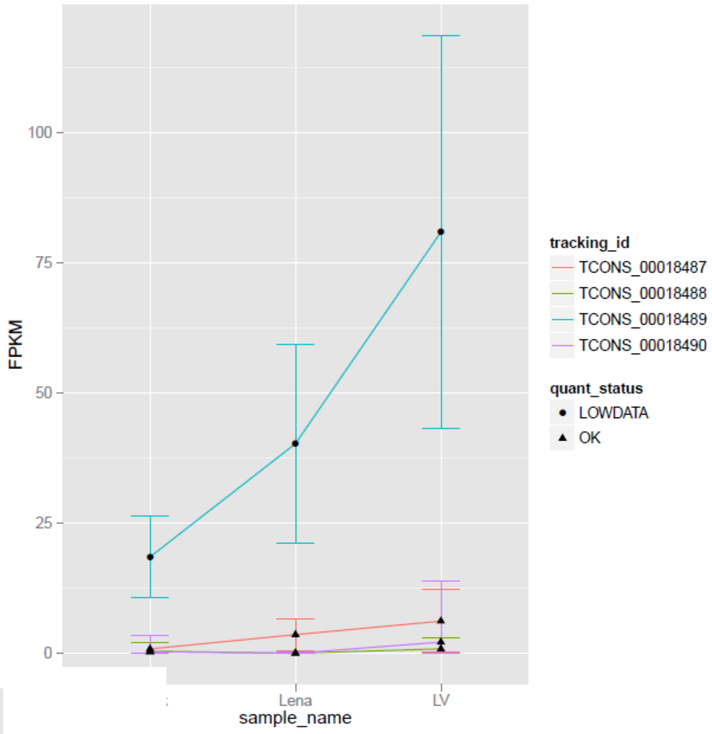

FPKM
100
75
50
25
0
Lena
LV
sample_name
tracking_id
TCONS_00018487
TCONS_00018488
TCONS_00018489
TCONS_00018490
quant_status
LOWDATA
OK


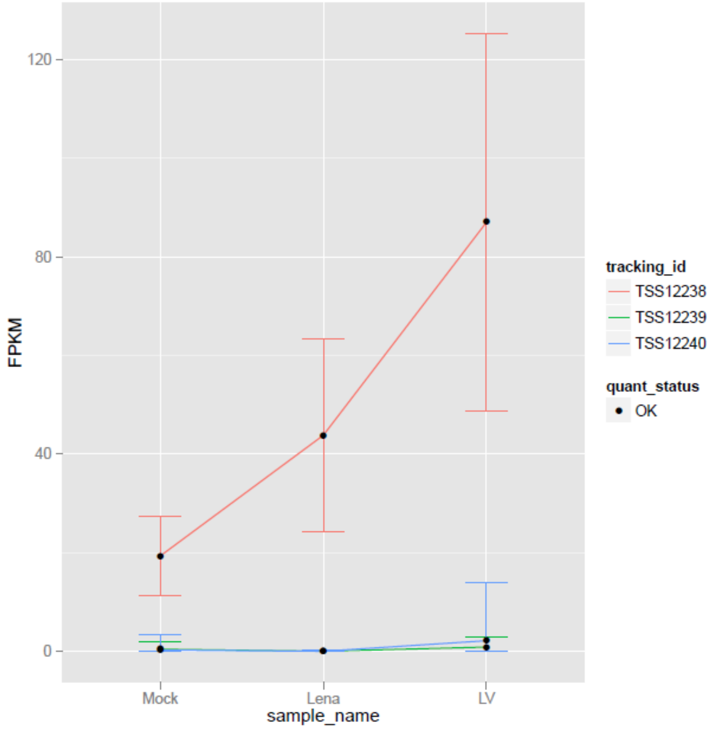

FPKM
120
80
40
0
Mock
Lena
LV
sample_name
tracking_id
TSS12238
TSS12239
TSS12240
quant_status
OK

# NFKBIA

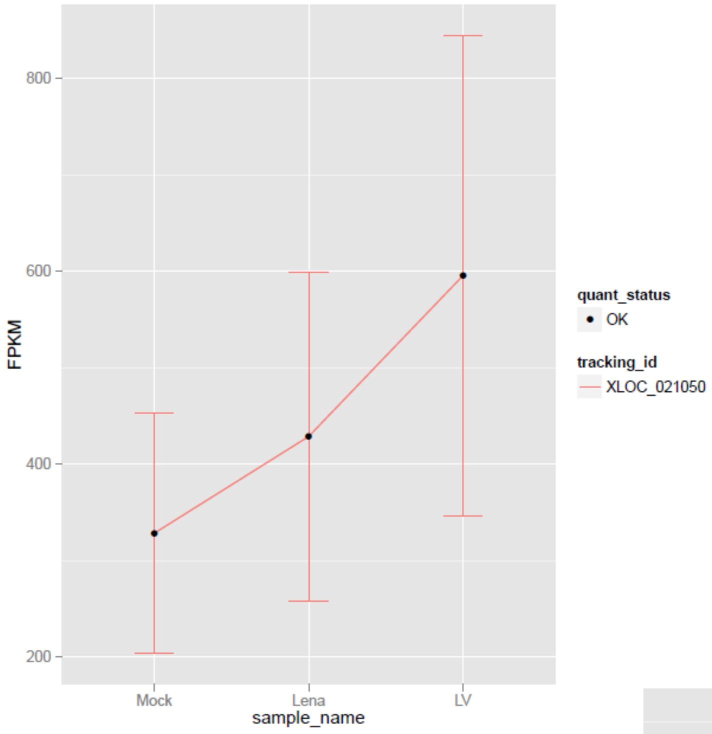

800
600
400
200
FPKM
Mock
Lena
LV
sample_name
quant_status
OK
tracking_id
XLOC_021050


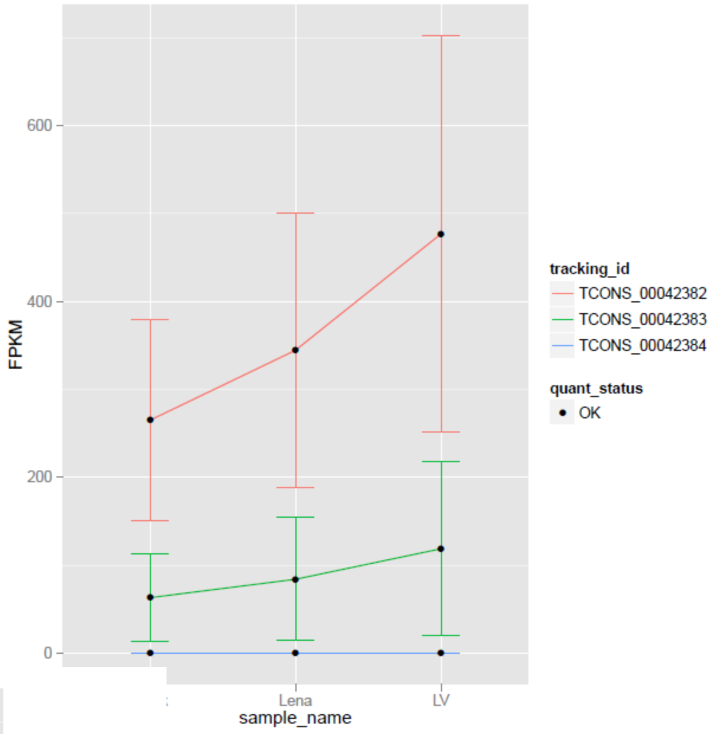

600
400
200
0
FPKM
Lena
LV
sample_name
tracking_id
TCONS_00042382
TCONS_00042383
TCONS_00042384
quant_status
OK


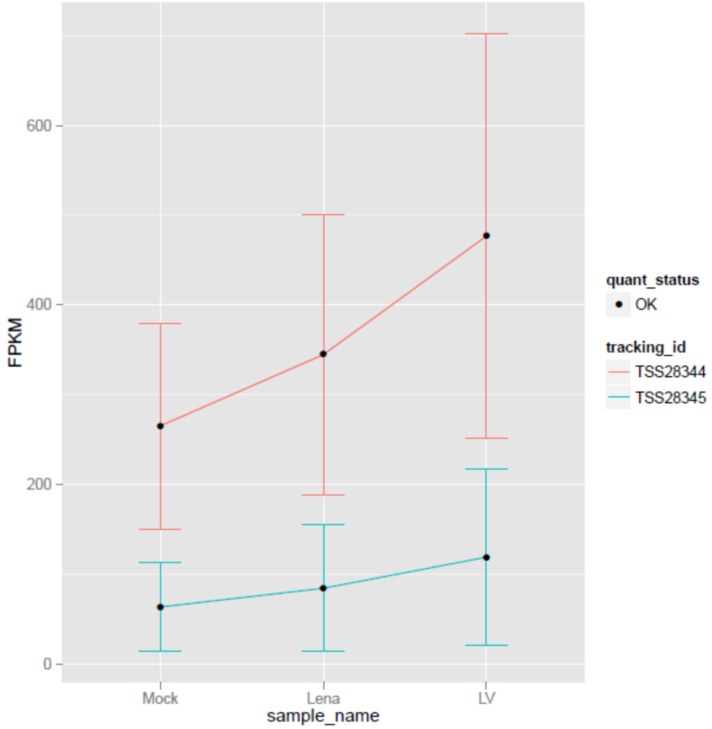

600
400
200
0
FPKM
Mock
Lena
LV
sample_name
quant_status
OK
tracking_id
TSS28344
TSS28345

# IFIT2

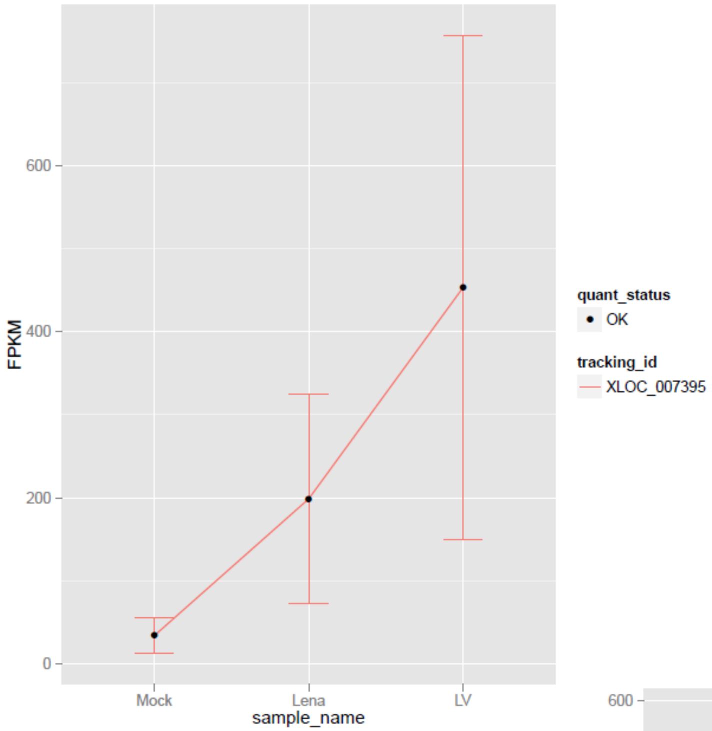

FPKM
600
400
200
0
Mock
Lena
LV
sample_name
quant_status
OK
tracking_id
XLOC_007395


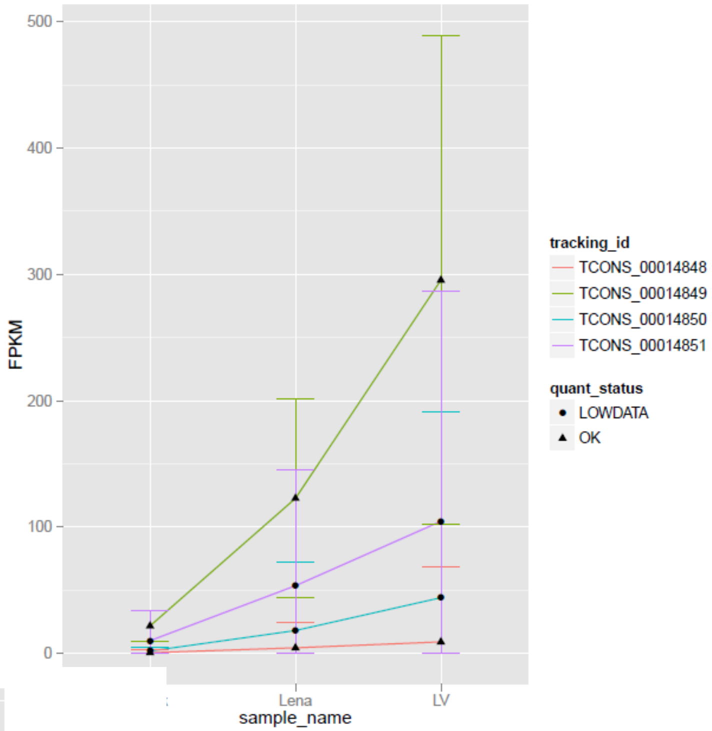

FPKM
500
400
300
200
100
0
Lena
LV
sample_name
tracking_id
TCONS_00014848
TCONS_00014849
TCONS_00014850
TCONS_00014851
quant_status
LOWDATA
OK


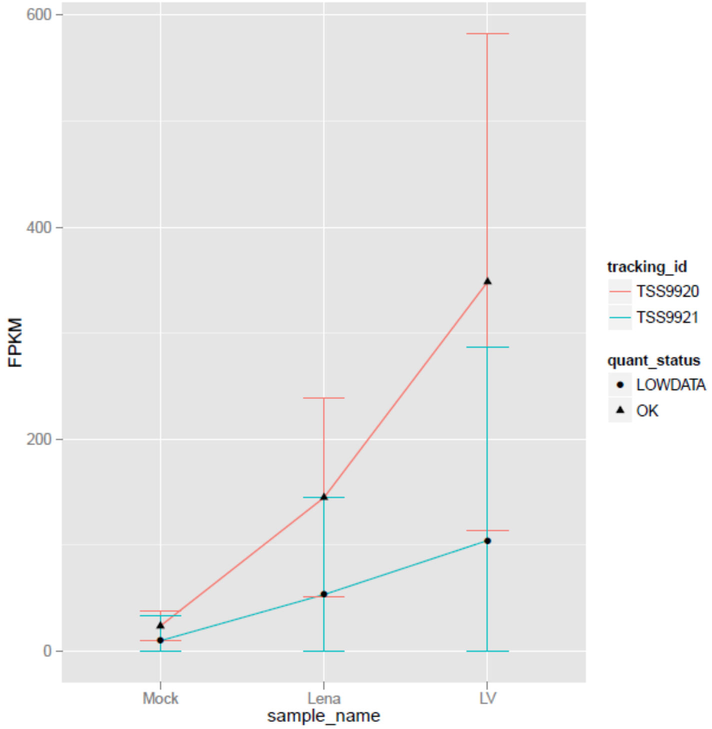

FPKM
600
400
200
0
Mock
Lena
LV
sample_name
tracking_id
TSS9920
TSS9921
quant_status
LOWDATA
OK

# PIK3R1

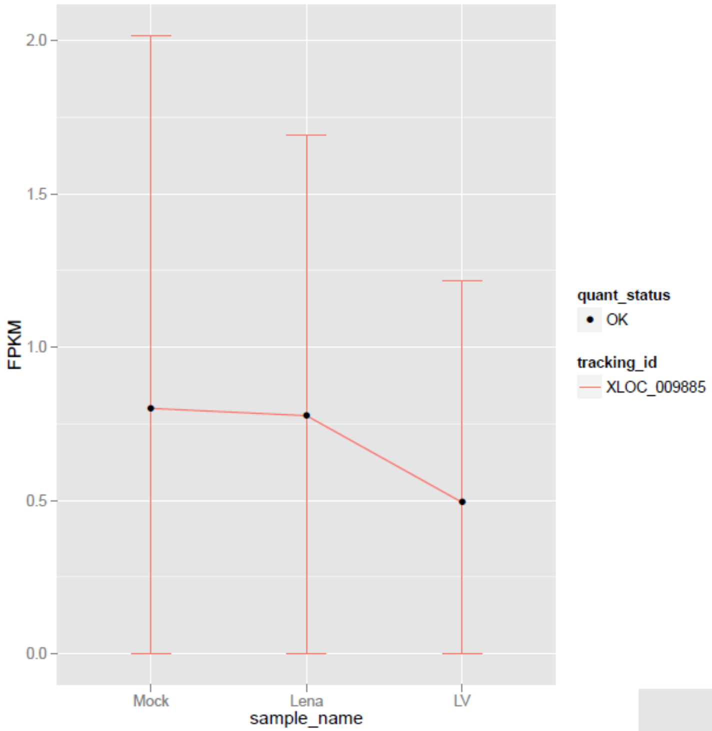

FPKM
2.0
1.5
1.0
0.5
0.0
Mock
Lena
LV
sample_name
quant_status
OK
tracking_id
XLOC_009885


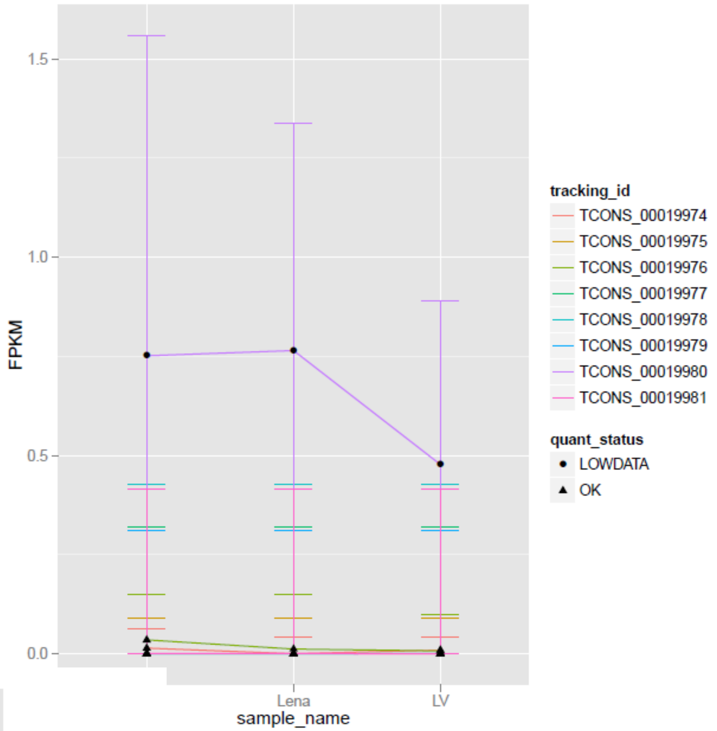

FPKM
1.5
1.0
0.5
0.0
Lena
LV
sample_name
tracking_id
TCONS_00019974
TCONS_00019975
TCONS_00019976
TCONS_00019977
TCONS_00019978
TCONS_00019979
TCONS_00019980
TCONS_00019981
quant_status
LOWDATA
OK


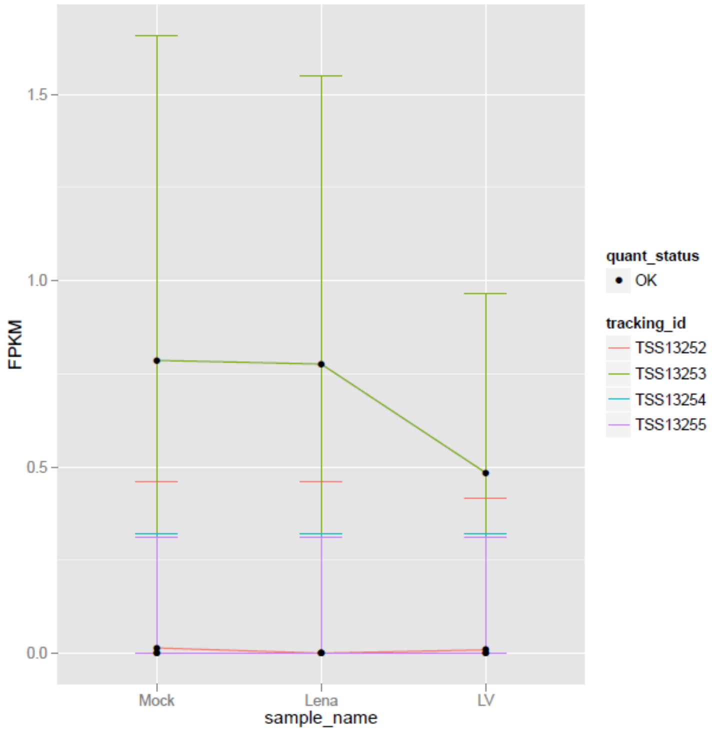

FPKM
1.5
1.0
0.5
0.0
Mock
Lena
LV
sample_name
quant_status
OK
tracking_id
TSS13252
TSS13253
TSS13254
TSS13255

# RNASEL

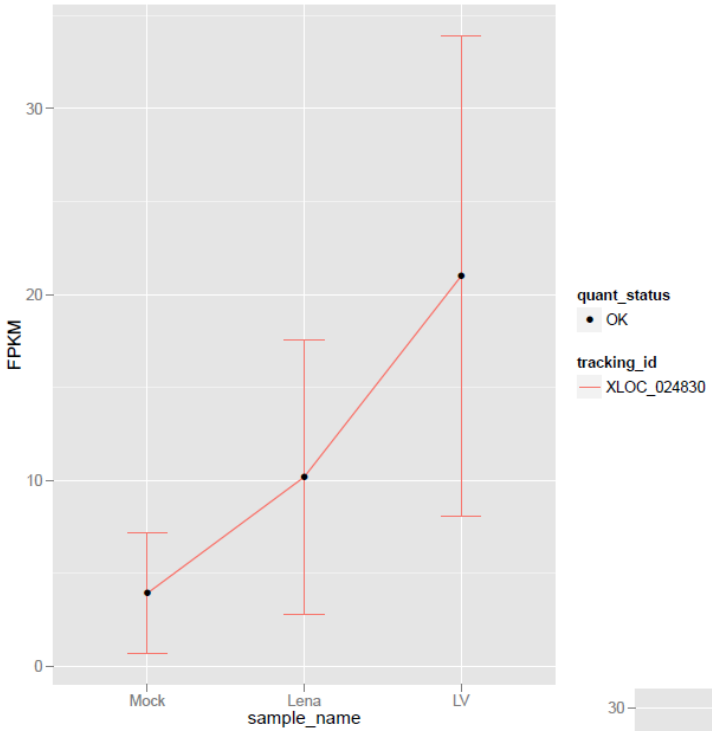

FPKM
30
20
10
0
Mock
Lena
LV
sample_name
quant_status
OK
tracking_id
XLOC_024830


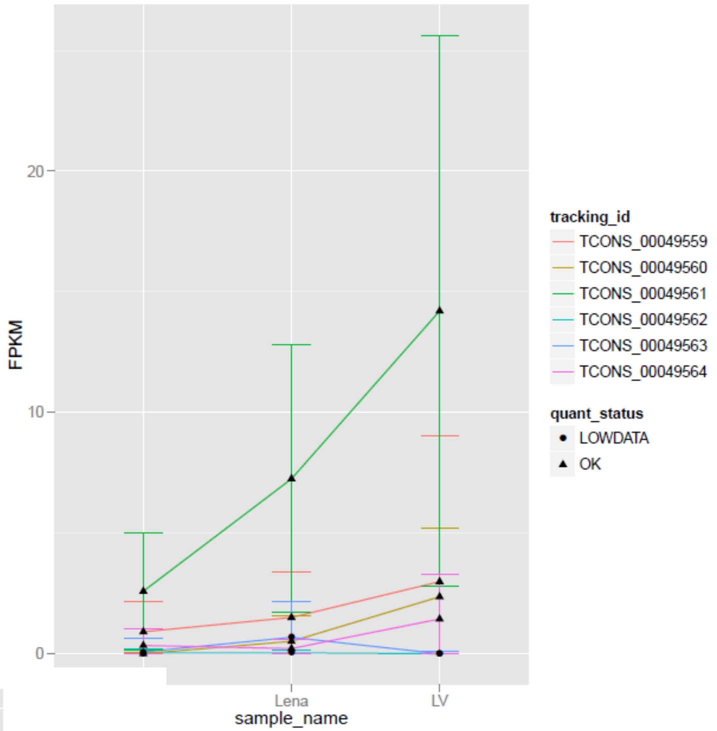

FPKM
20
10
0
Lena
LV
sample_name
tracking_id
TCONS_00049559
TCONS_00049560
TCONS_00049561
TCONS_00049562
TCONS_00049563
TCONS_00049564
quant_status
LOWDATA
OK


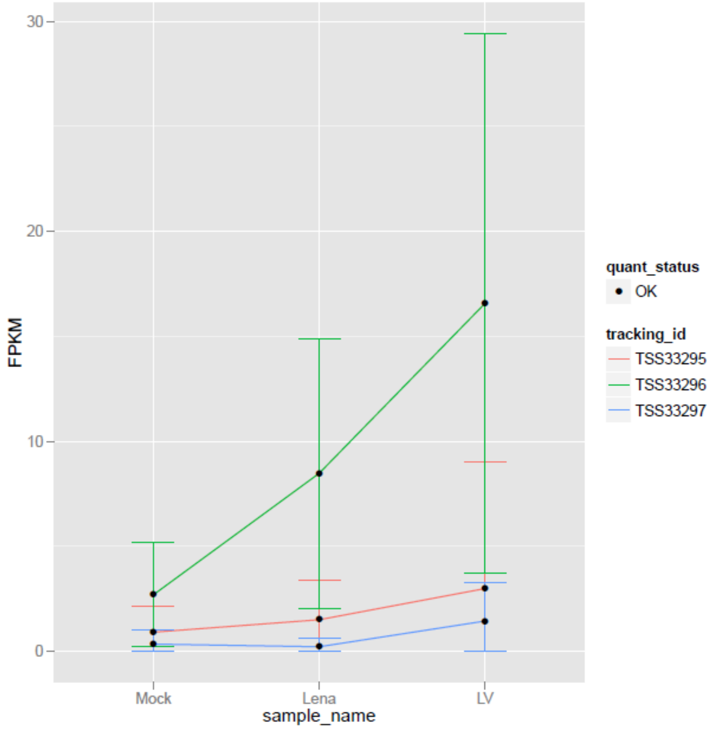

FPKM
30
20
10
0
Mock
Lena
LV
sample_name
quant_status
OK
tracking_id
TSS33295
TSS33296
TSS33297

# TLR7

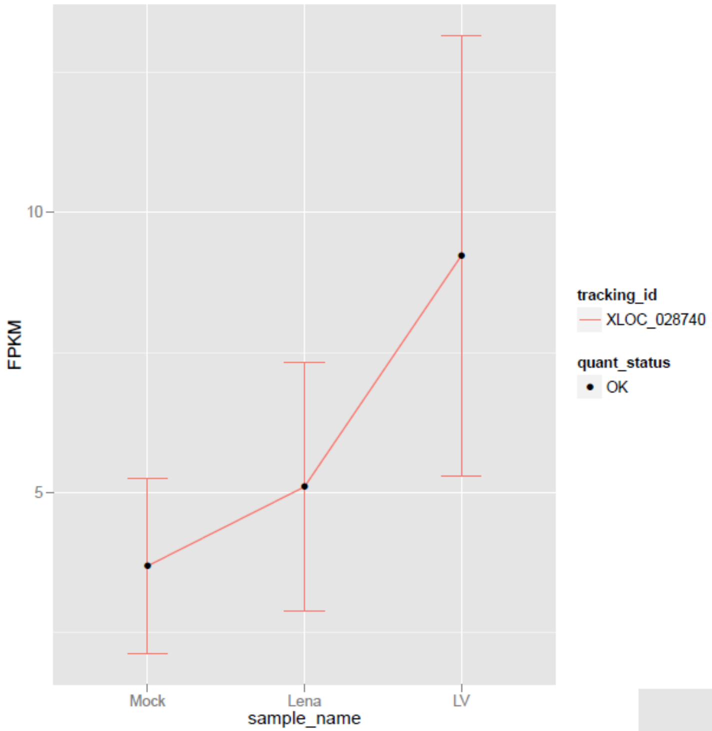
FPKM
10
5
Mock
Lena
LV
sample_name
tracking_id
XLOC_028740
quant_status
OK

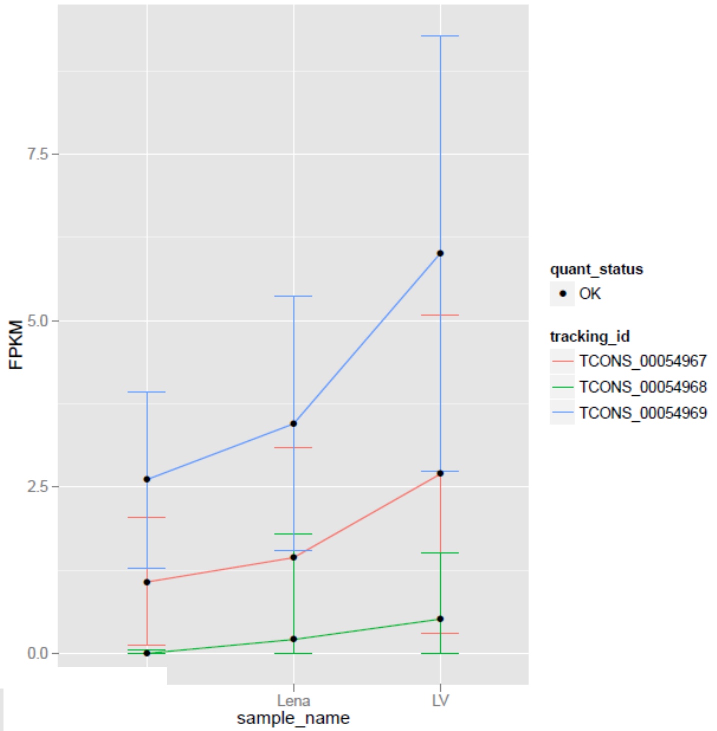
FPKM
7.5
5.0
2.5
0.0
Lena
LV
sample_name
quant_status
OK
tracking_id
TCONS_00054967
TCONS_00054968
TCONS_00054969

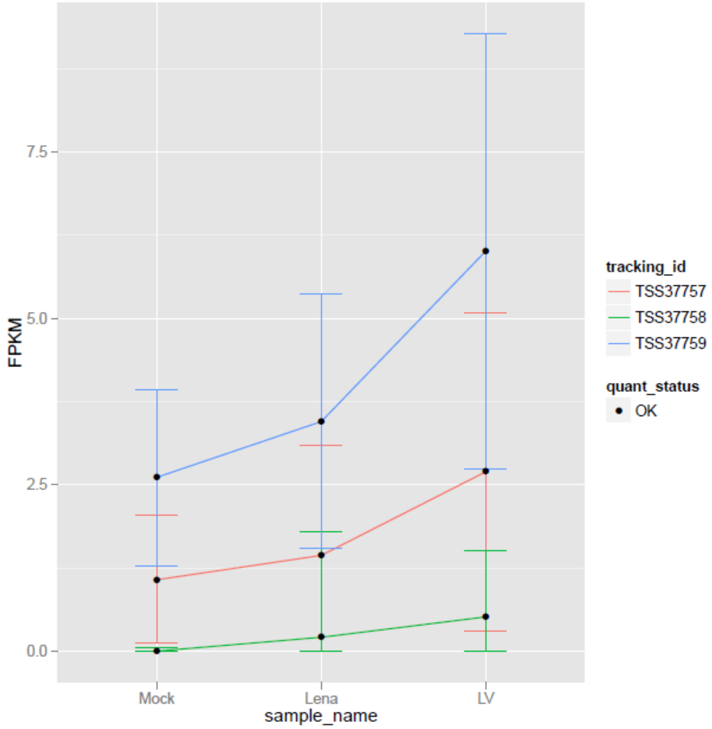
FPKM
7.5
5.0
2.5
0.0
Mock
Lena
LV
sample_name
tracking_id
TSS37757
TSS37758
TSS37759
quant_status
OK

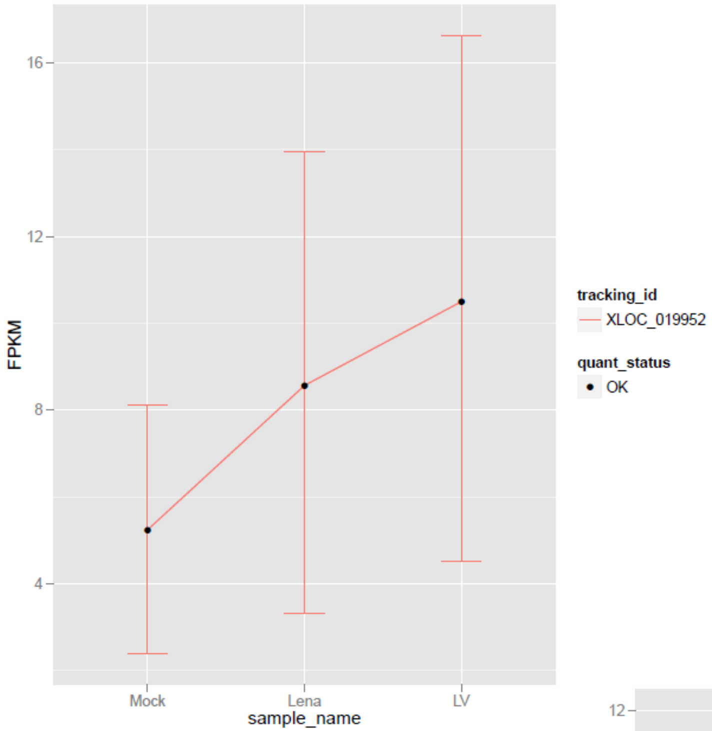

FPKM
16
12
8
4
Mock
Lena
LV
sample_name
tracking_id
XLOC_019952
quant_status
OK


# PIK3CD

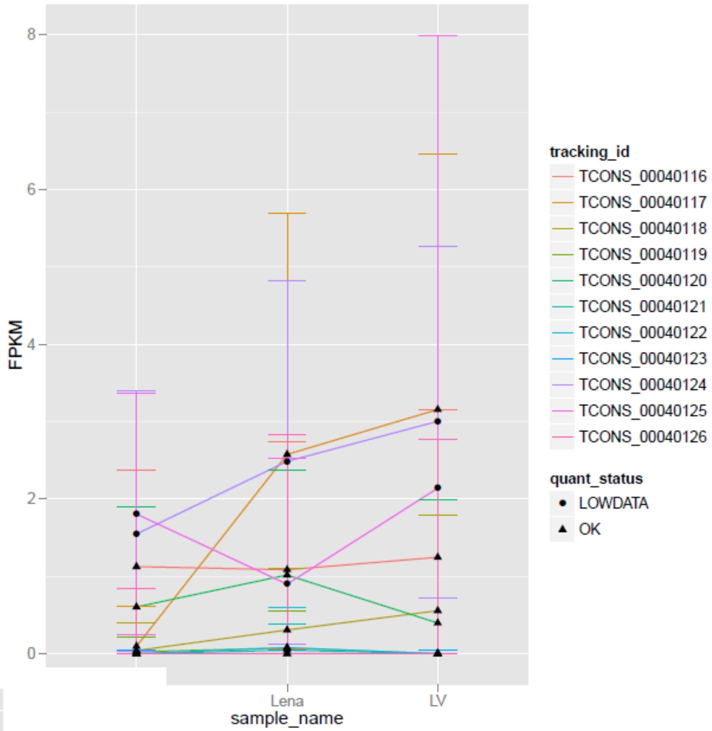

FPKM
8
6
4
2
0
Lena
LV
sample_name
tracking_id
TCONS_00040116
TCONS_00040117
TCONS_00040118
TCONS_00040119
TCONS_00040120
TCONS_00040121
TCONS_00040122
TCONS_00040123
TCONS_00040124
TCONS_00040125
TCONS_00040126
quant_status
LOWDATA
OK


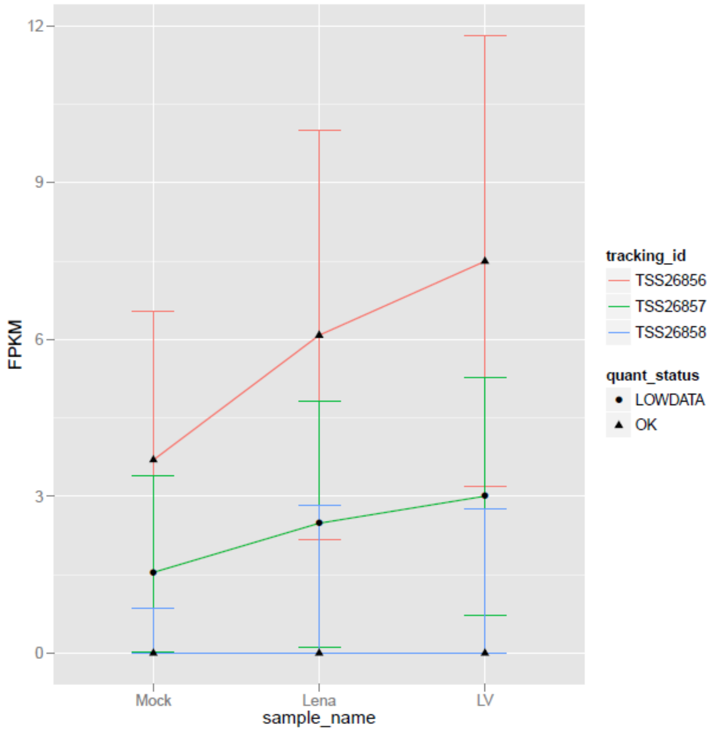

FPKM
12
9
6
3
0
Mock
Lena
LV
sample_name
tracking_id
TSS26856
TSS26857
TSS26858
quant_status
LOWDATA
OK

# EIF2AK2

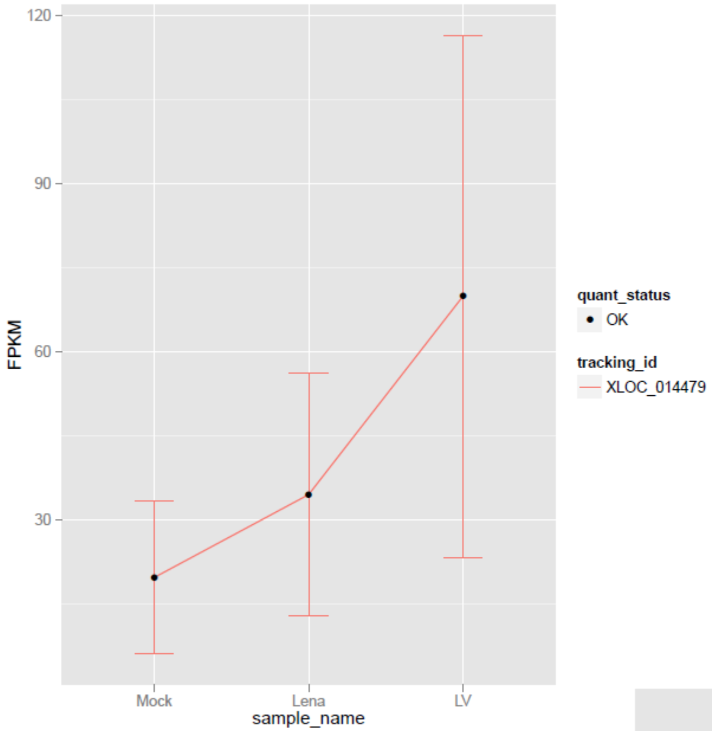

120
90
60
30
FPKM
Mock
Lena
LV
sample_name
quant_status
OK
tracking_id
XLOC_014479


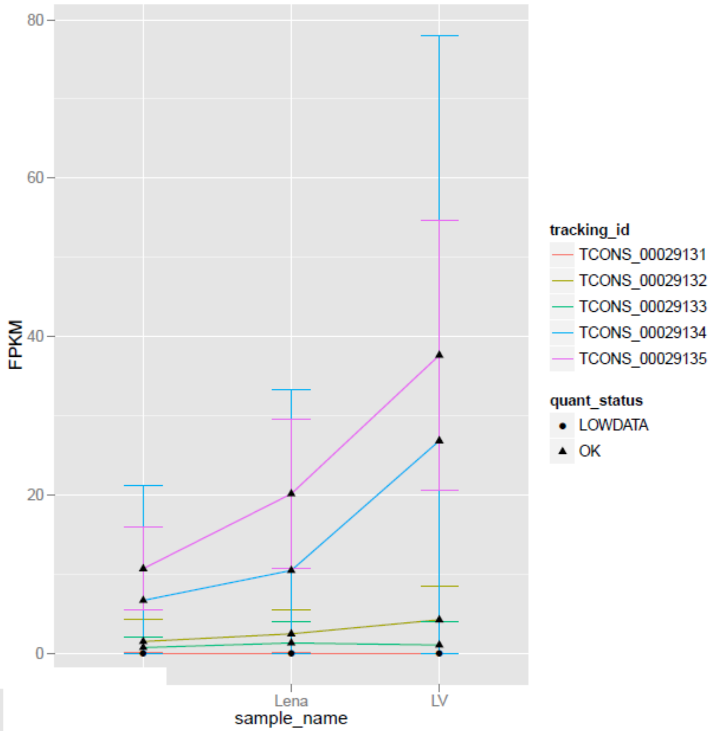

80
60
40
20
0
FPKM
Lena
LV
sample_name
tracking_id
TCONS_00029131
TCONS_00029132
TCONS_00029133
TCONS_00029134
TCONS_00029135
quant_status
LOWDATA
OK


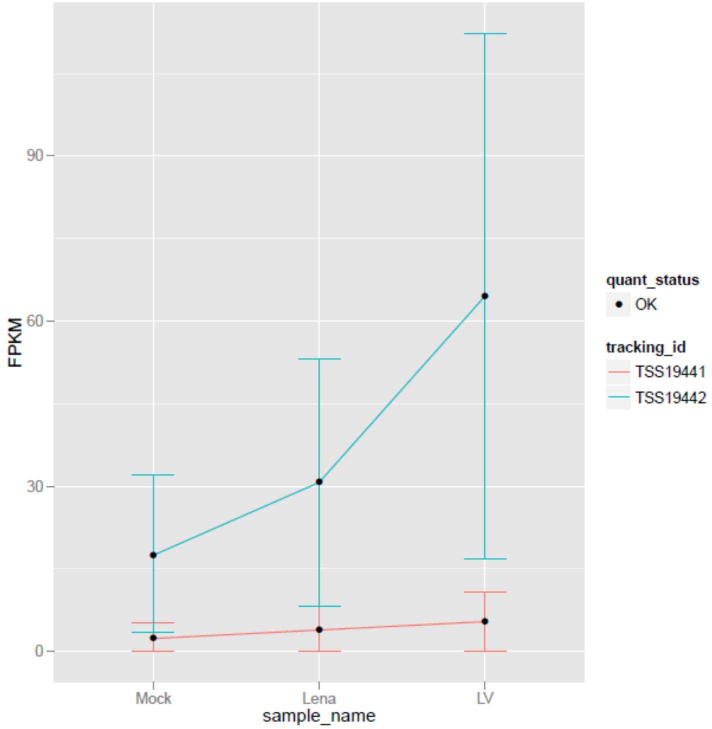

90
60
30
0
FPKM
Mock
Lena
LV
sample_name
quant_status
OK
tracking_id
TSS19441
TSS19442

# TLR3

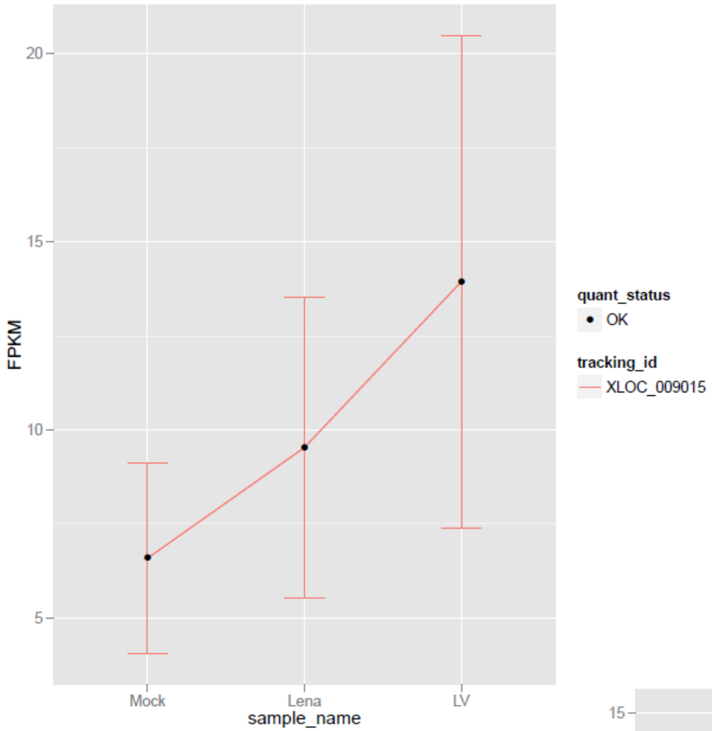

FPKM
20
15
10
5
Mock
Lena
LV
sample_name
quant_status
OK
tracking_id
XLOC_009015


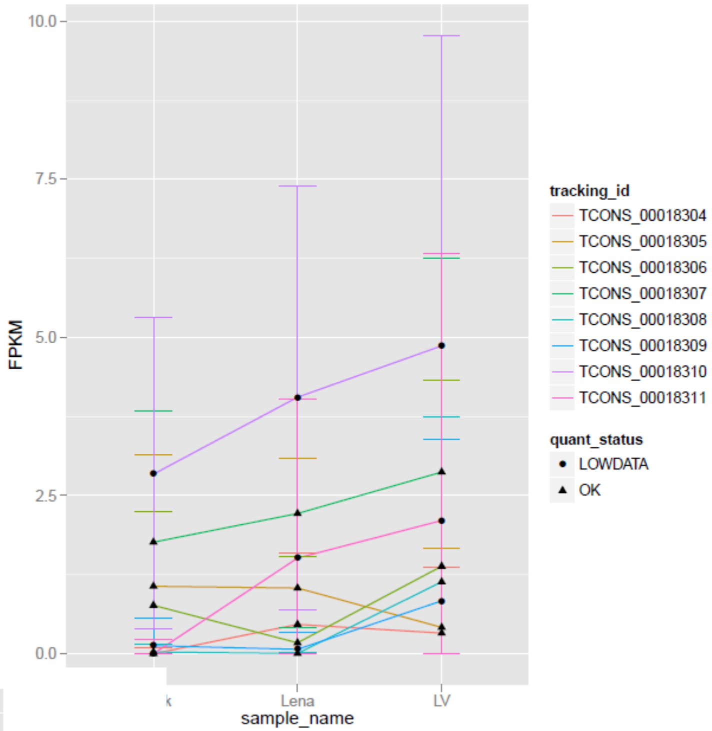

FPKM
10.0
7.5
5.0
2.5
0.0
Lena
LV
sample_name
tracking_id
TCONS_00018304
TCONS_00018305
TCONS_00018306
TCONS_00018307
TCONS_00018308
TCONS_00018309
TCONS_00018310
TCONS_00018311
quant_status
LOWDATA
OK


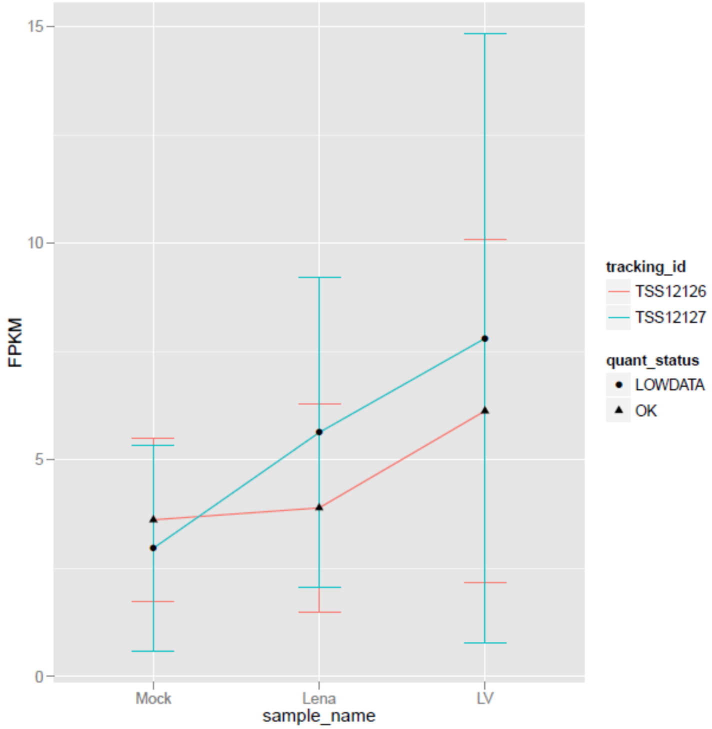

FPKM
15
10
5
0
Mock
Lena
LV
sample_name
tracking_id
TSS12126
TSS12127
quant_status
LOWDATA
OK

# TNFSF10

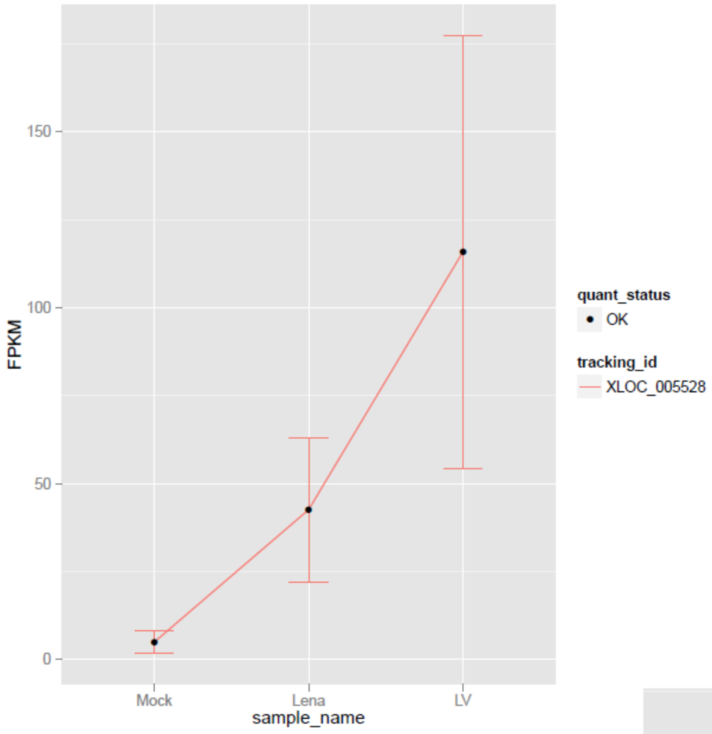

FPKM
150
100
50
0
Mock
Lena
LV
sample_name
quant_status
OK
tracking_id
XLOC_005528


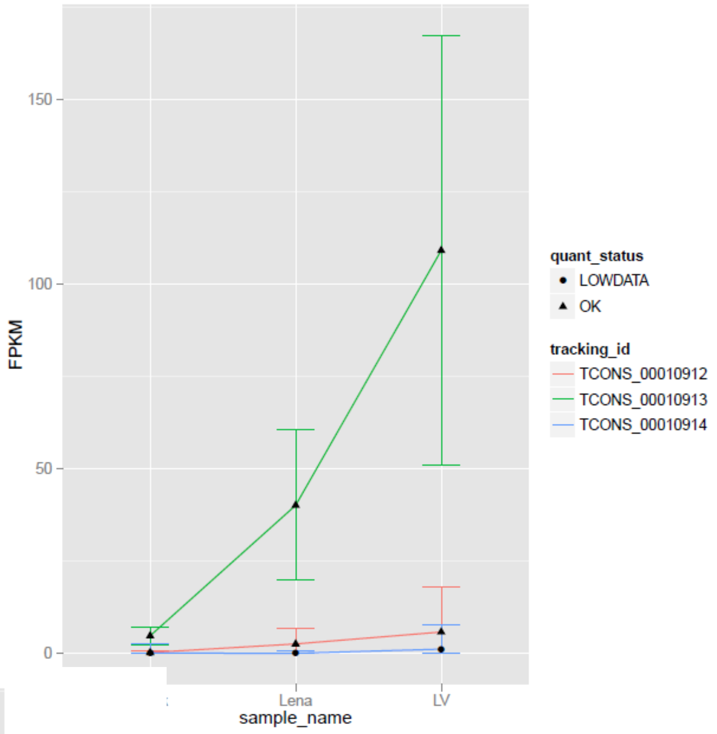

FPKM
150
100
50
0
Lena
LV
sample_name
quant_status
LOWDATA
OK
tracking_id
TCONS_00010912
TCONS_00010913
TCONS_00010914


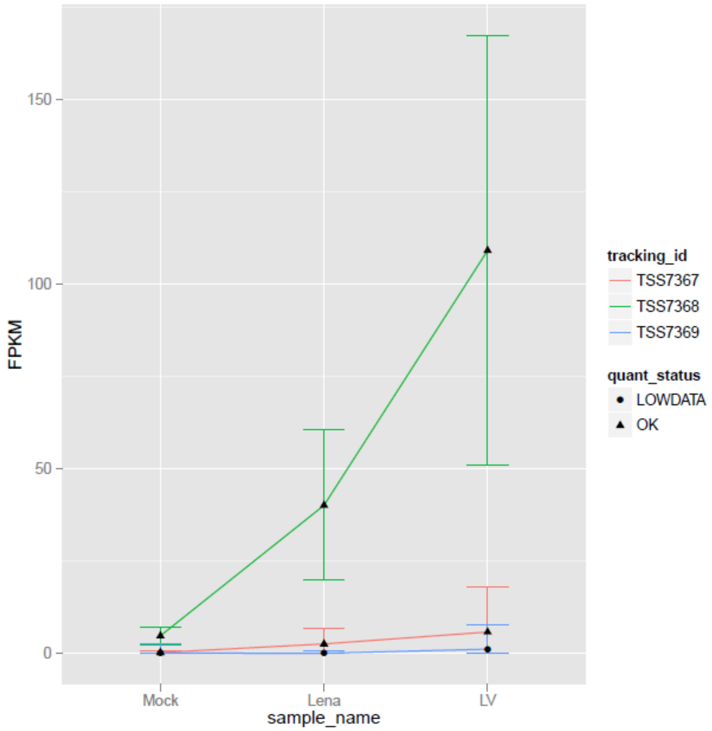

FPKM
150
100
50
0
Mock
Lena
LV
sample_name
tracking_id
TSS7367
TSS7368
TSS7369
quant_status
LOWDATA
OK

# PARP11

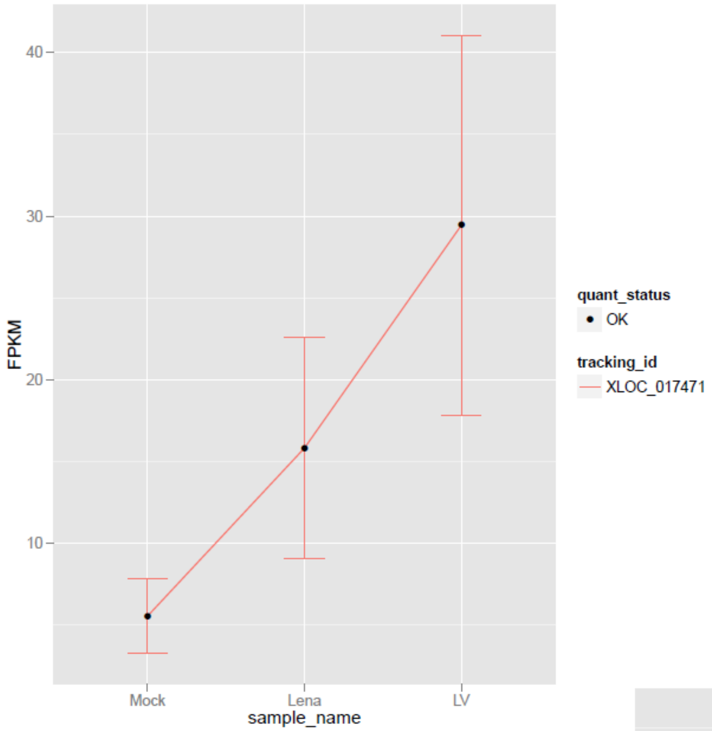

40
30
20
10
FPKM
Mock
Lena
LV
sample_name
quant_status
OK
tracking_id
XLOC_017471


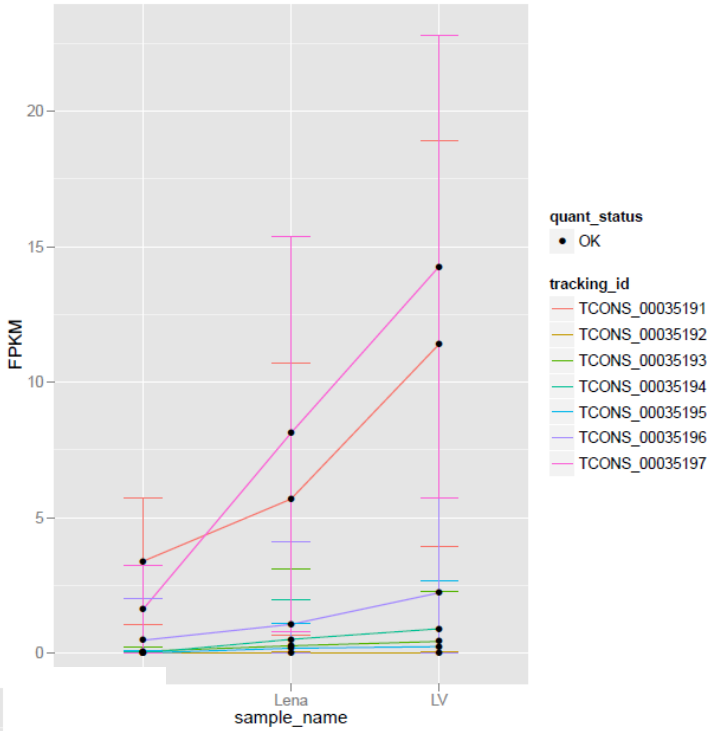

20
15
10
5
0
FPKM
Lena
LV
sample_name
quant_status
OK
tracking_id
TCONS_00035191
TCONS_00035192
TCONS_00035193
TCONS_00035194
TCONS_00035195
TCONS_00035196
TCONS_00035197


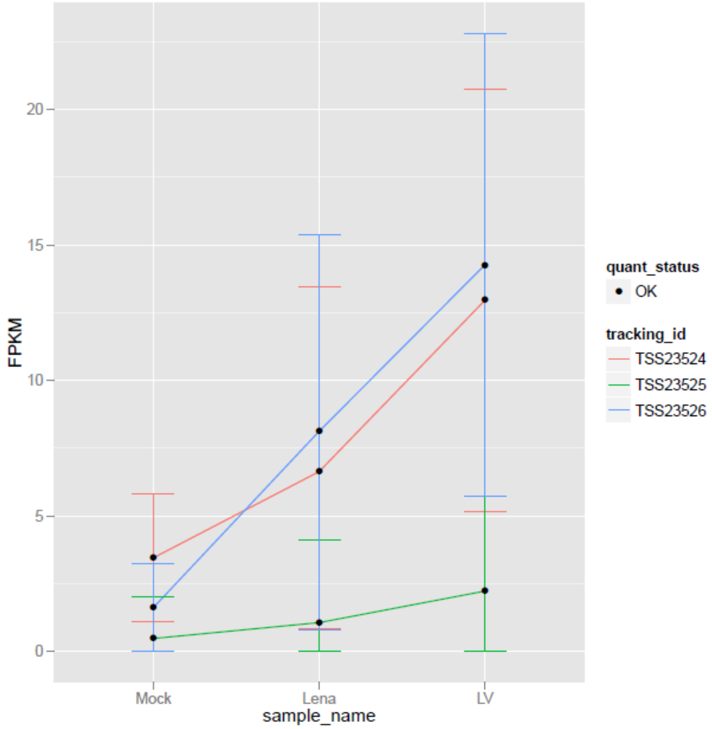

20
15
10
5
0
FPKM
Mock
Lena
LV
sample_name
quant_status
OK
tracking_id
TSS23524
TSS23525
TSS23526

# TNFRSF10A

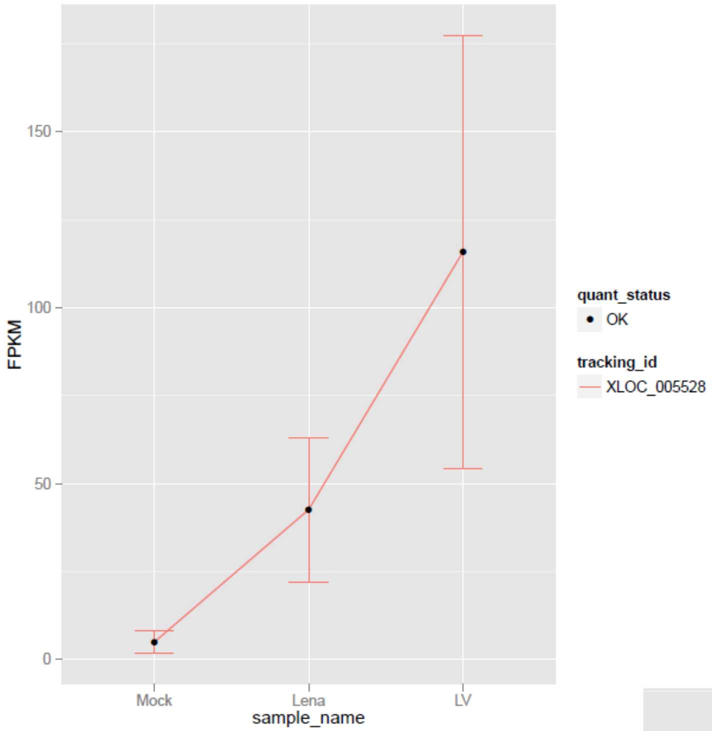
FPKM
150
100
50
0
Mock
Lena
LV
sample_name
quant_status
OK
tracking_id
XLOC_005528

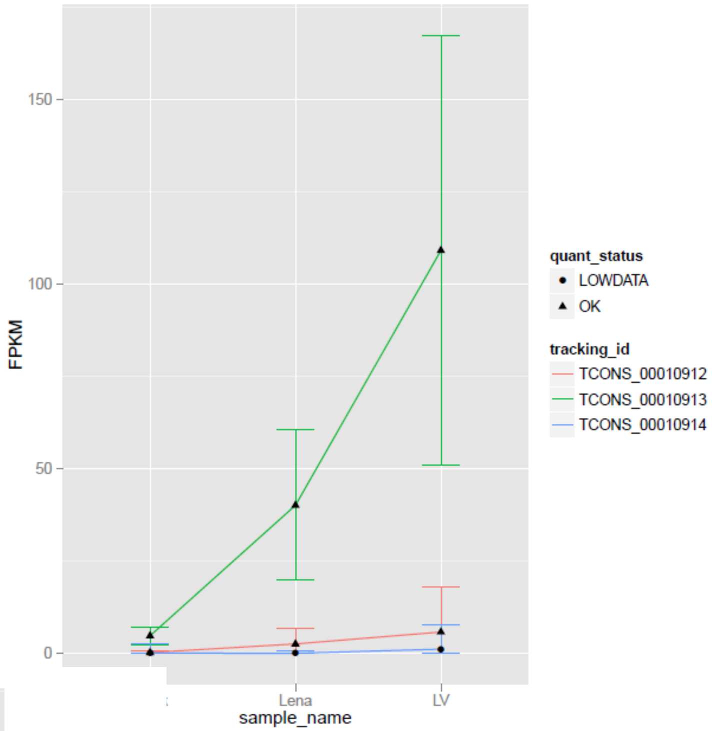
FPKM
150
100
50
0
Lena
LV
sample_name
quant_status
LOWDATA
OK
tracking_id
TCONS_00010912
TCONS_00010913
TCONS_00010914

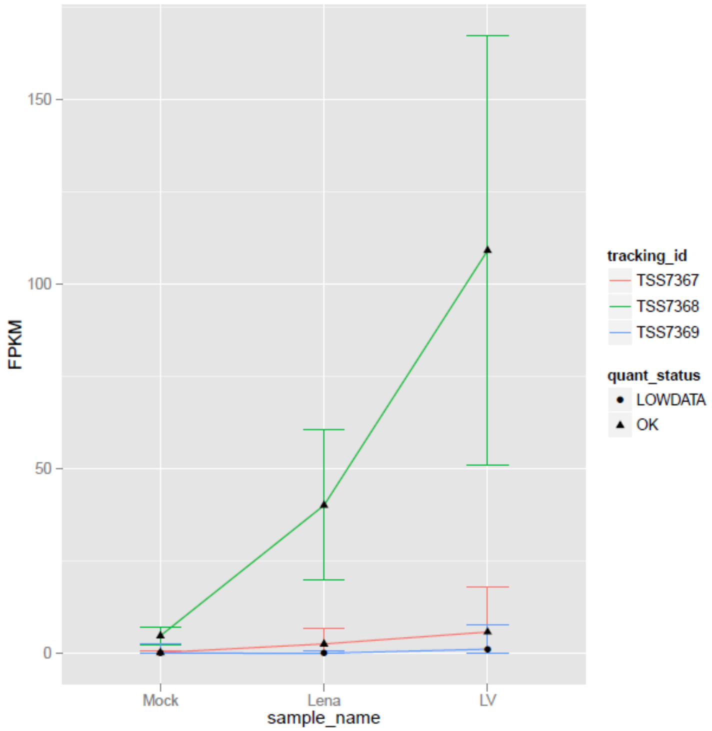
FPKM
150
100
50
0
Mock
Lena
LV
sample_name
tracking_id
TSS7367
TSS7368
TSS7369
quant_status
LOWDATA
OK

# PARP9

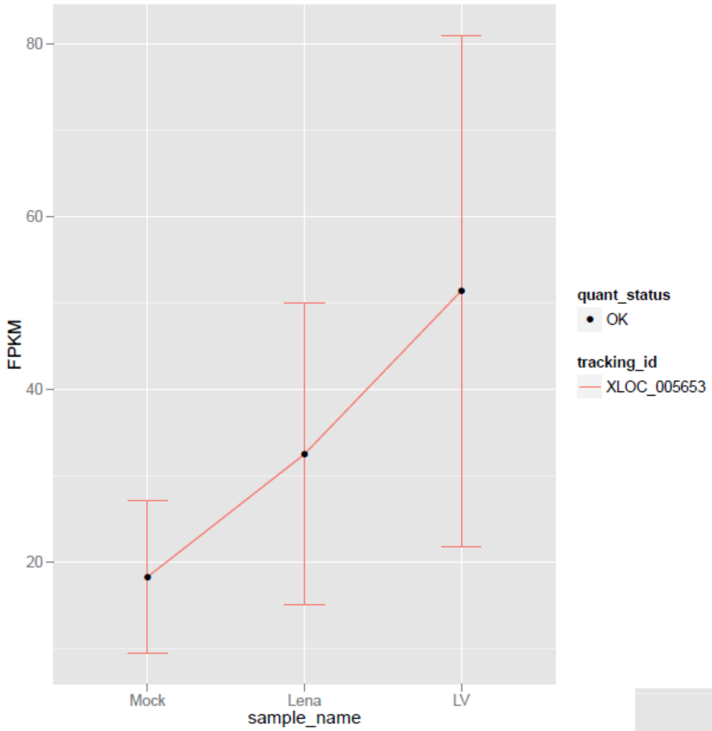
FPKM
80
60
40
20
Mock
Lena
LV
sample_name
quant_status
OK
tracking_id
XLOC_005653

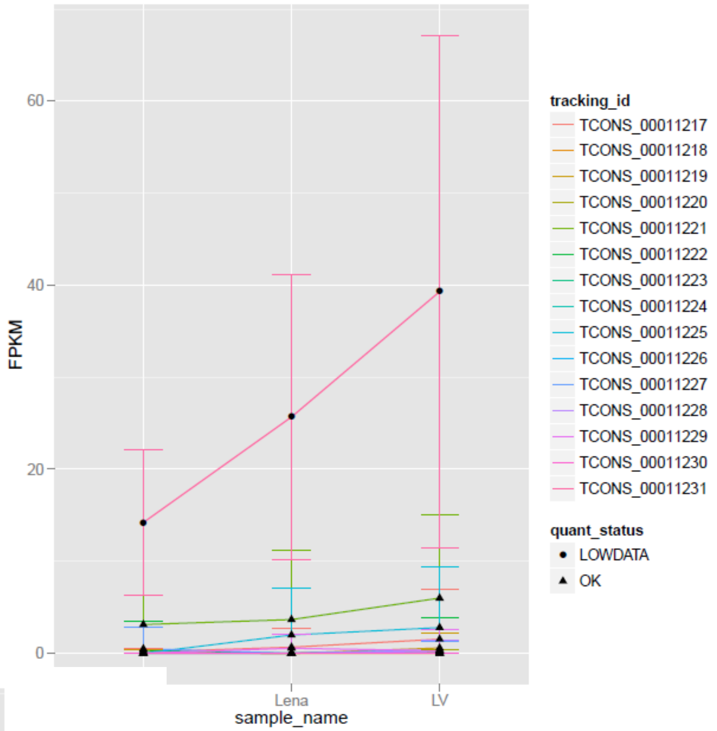
FPKM
60
40
20
0
Lena
LV
sample_name
tracking_id
TCONS_00011217
TCONS_00011218
TCONS_00011219
TCONS_00011220
TCONS_00011221
TCONS_00011222
TCONS_00011223
TCONS_00011224
TCONS_00011225
TCONS_00011226
TCONS_00011227
TCONS_00011228
TCONS_00011229
TCONS_00011230
TCONS_00011231
quant_status
LOWDATA
OK

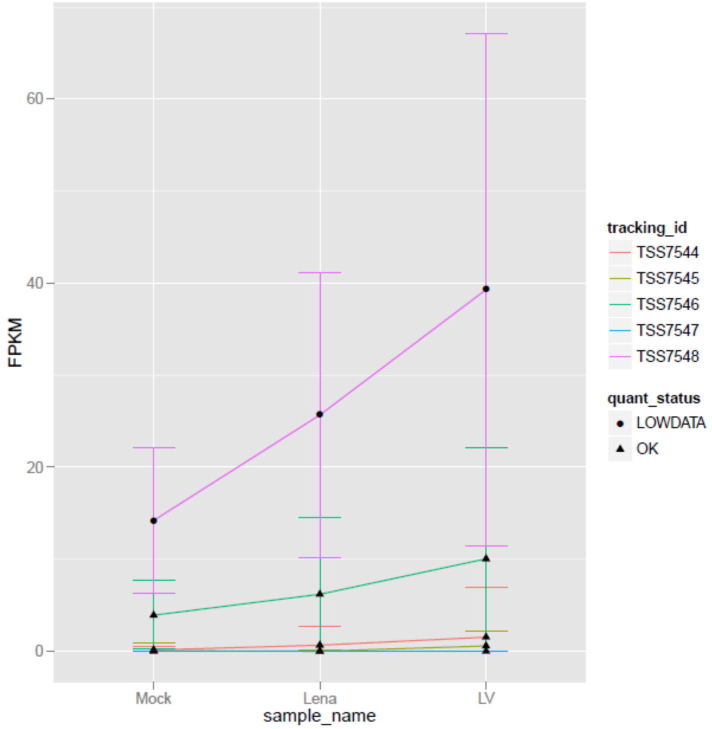
FPKM
60
40
20
0
Mock
Lena
LV
sample_name
tracking_id
TSS7544
TSS7545
TSS7546
TSS7547
TSS7548
quant_status
LOWDATA
OK

# PARP14

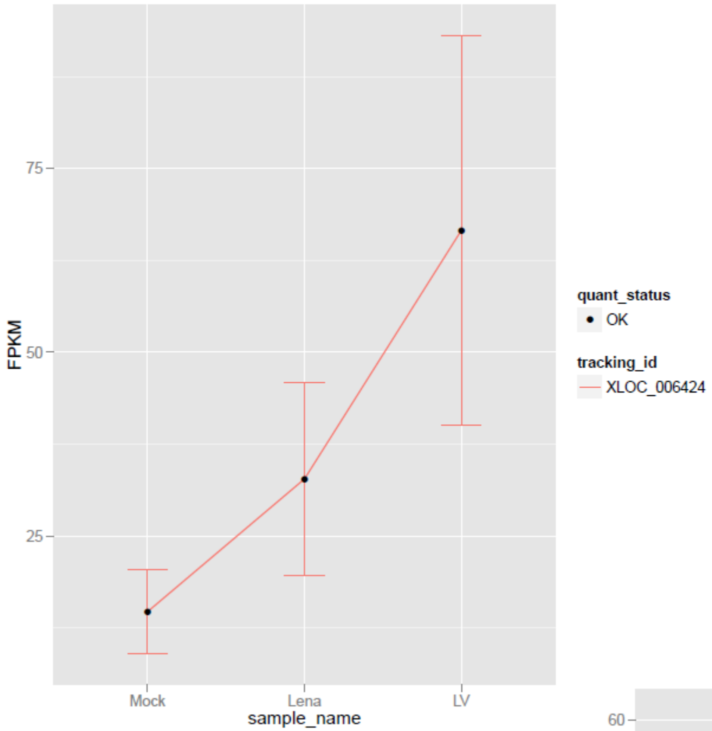

FPKM
75
50
25
Mock
Lena
LV
sample_name
quant_status
OK
tracking_id
XLOC_006424


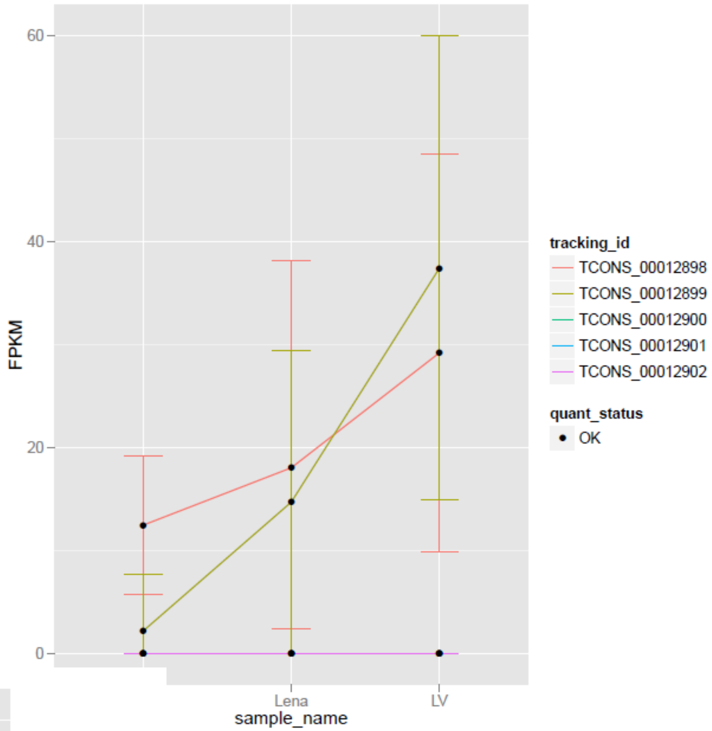

FPKM
60
40
20
0
Lena
LV
sample_name
tracking_id
TCONS_00012898
TCONS_00012899
TCONS_00012900
TCONS_00012901
TCONS_00012902
quant_status
OK


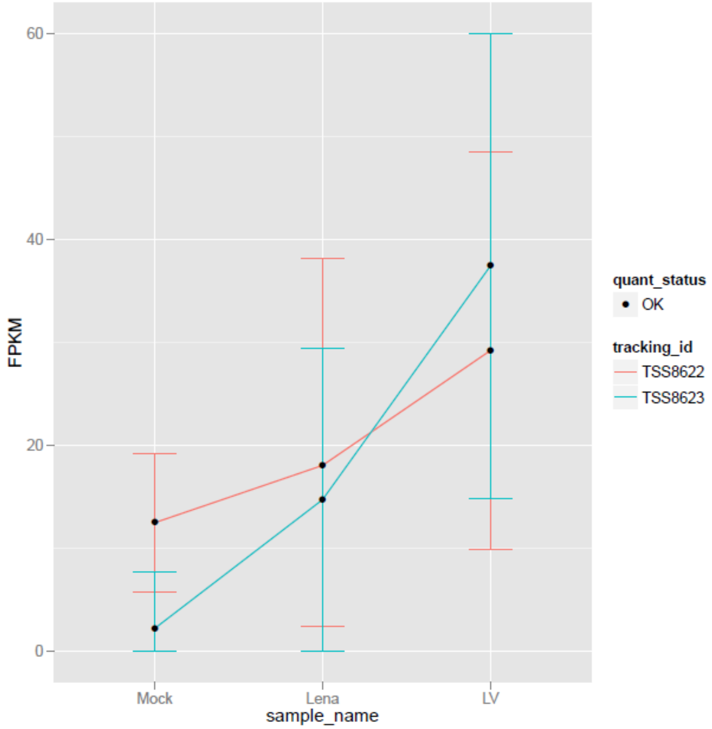

FPKM
60
40
20
0
Mock
Lena
LV
sample_name
quant_status
OK
tracking_id
TSS8622
TSS8623

# CXCL10

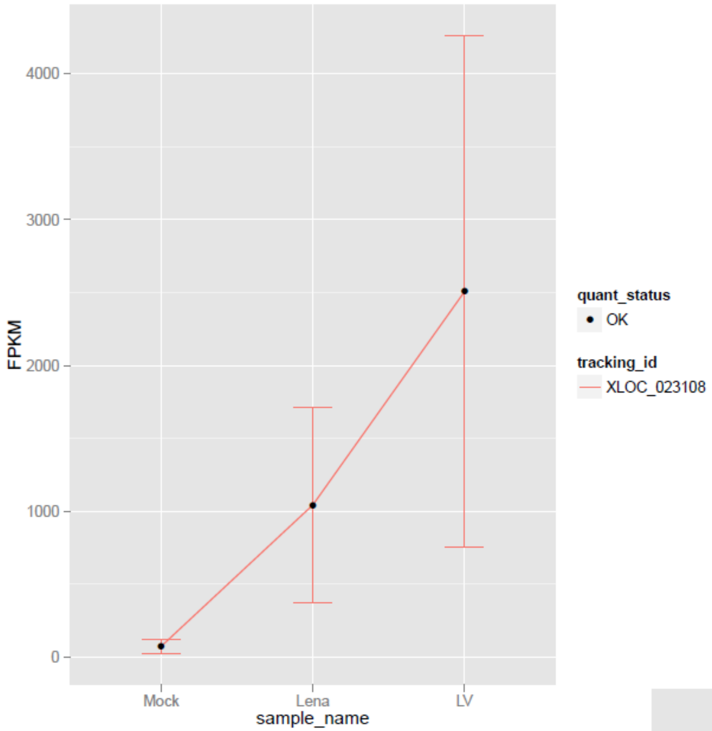
FPKM
4000
3000
2000
1000
0
Mock
Lena
LV
sample_name
quant_status
OK
tracking_id
XLOC_023108

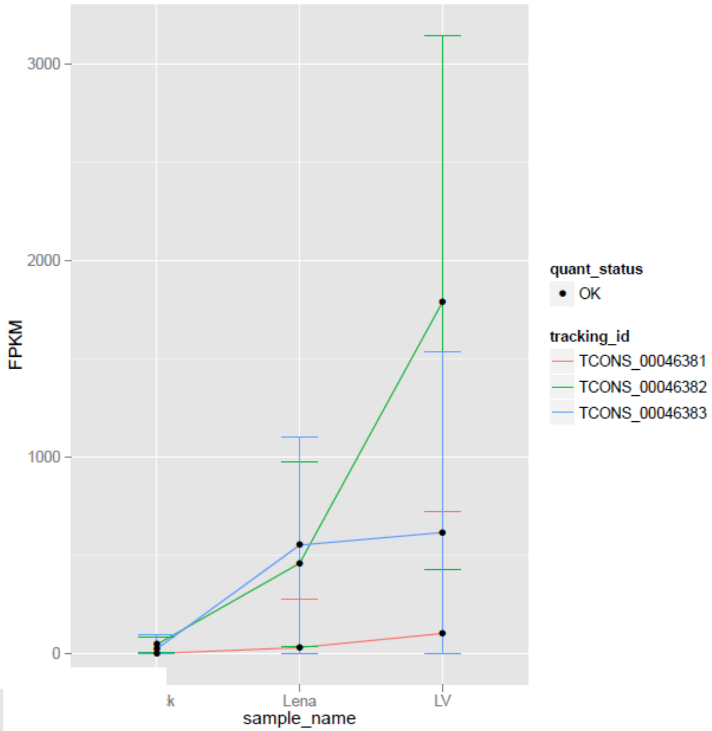
FPKM
3000
2000
1000
0
k
Lena
LV
sample_name
quant_status
OK
tracking_id
TCONS_00046381
TCONS_00046382
TCONS_00046383

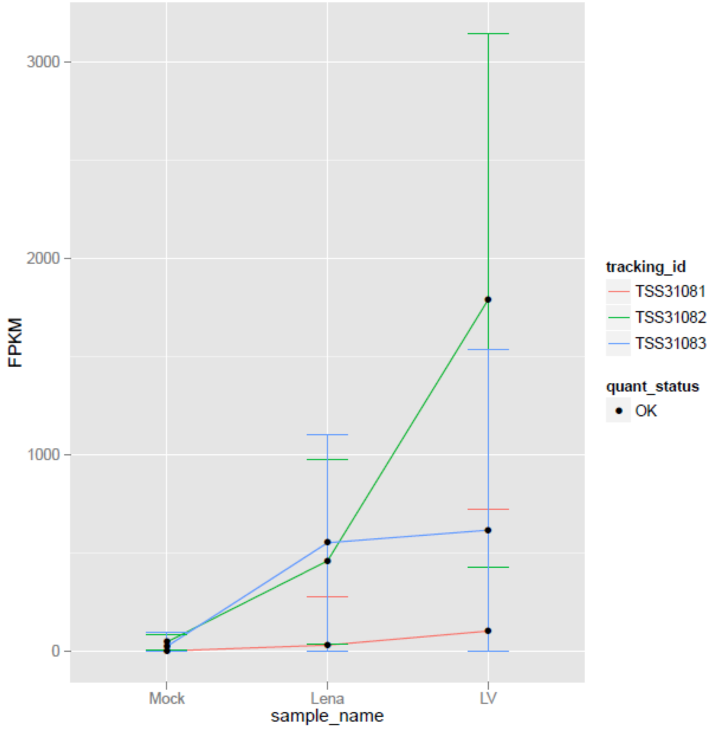
FPKM
3000
2000
1000
0
Mock
Lena
LV
sample_name
tracking_id
TSS31081
TSS31082
TSS31083
quant_status
OK

# IL15

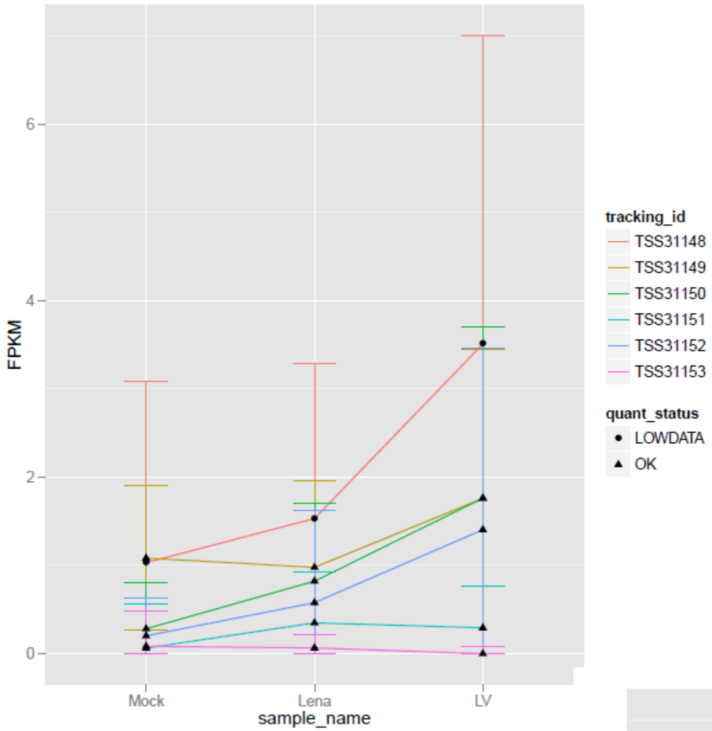
FPKM
sample_name
Mock
Lena
LV
0
2
4
6
tracking_id
TSS31148
TSS31149
TSS31150
TSS31151
TSS31152
TSS31153
quant_status
LOWDATA
OK

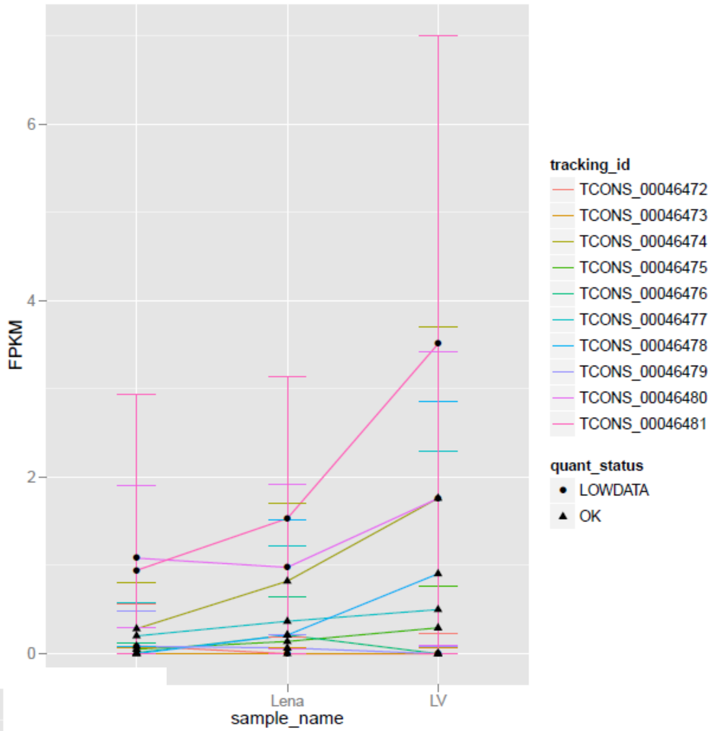
FPKM
sample_name
Lena
LV
0
2
4
6
tracking_id
TCONS_00046472
TCONS_00046473
TCONS_00046474
TCONS_00046475
TCONS_00046476
TCONS_00046477
TCONS_00046478
TCONS_00046479
TCONS_00046480
TCONS_00046481
quant_status
LOWDATA
OK

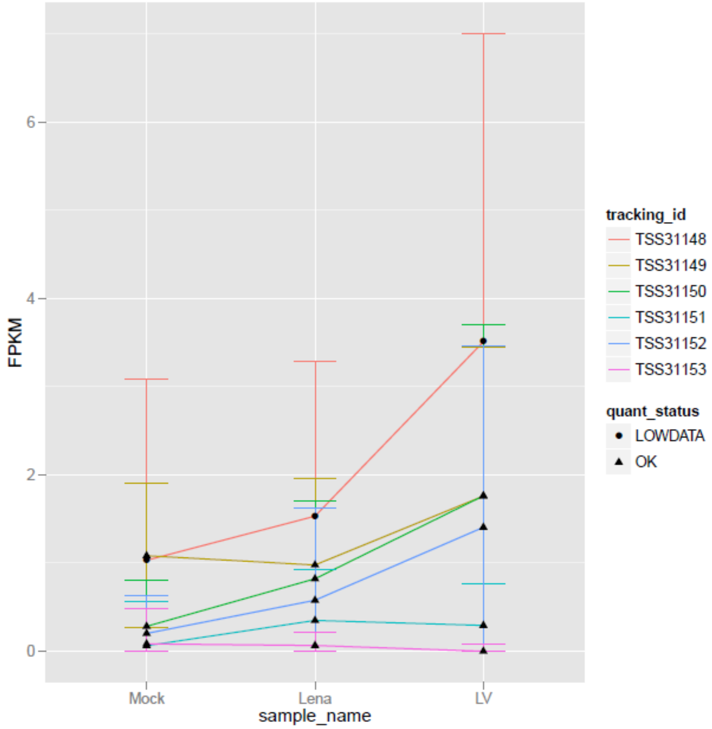
FPKM
sample_name
Mock
Lena
LV
0
2
4
6
tracking_id
TSS31148
TSS31149
TSS31150
TSS31151
TSS31152
TSS31153
quant_status
LOWDATA
OK
