# Supplementary material for: RNA-Sequence Analysis of Primary Alveolar Macrophages after In Vitro Infection with Porcine Reproductive and Respiratory Syndrome Virus Strains of Differing Virulence
Source: PLoS One. 2014 Mar 18;9(3):e91918. doi: 10.1371/journal.pone.0091918 (PMC3958415; doi:10.1371/journal.pone.0091918)
Supplement: Table S1 — Summary of the reads generated per sample (mock, LV and Lena). A. Numbers of RNA-Seq reads generated per sample (mock, LV and Lena). B. RNA-Seq reads eliminated following quality evaluation via FASTQC and Sickle. C. RNA-Seq reads mapped to the pig genome (Build Sus_scrofa.Sscrofa10.2.71.) using the TopHat v2.0.8 algorithm. (DOC) [file pone.0091918.s008.doc]

| **A. RNA-seq reads numbers generated per sample (Mock, LV, Lena)** | | | | | | | |
| --- | --- | --- | --- | --- | --- | --- | --- |
| **Sample name** | **File name** | | **Library code** | **Index** | **Total reads** | | **Total bases** |
| **PIG1_Mock_GDI-9** | **110818_SN365_A_s_7_1_seq_GDI-9.txt** | | **GDI-9** | **CAAAAT** | **20'417'364** | | **2'041'736'400** |
|
| **PIG1_Lena_GDI_10** | **110818_SN365_A_s_7_1_seq_GDI-10.txt** | | **GDI-10** | **GGTATA** | **21'469'434** | | **2'146'943'400** |
|
| **PIG1_LV_GDI_11** | **110818_SN365_A_s_7_1_seq_GDI-11.txt** | | **GDI-11** | **TCCGCT** | **20'023'158** | | **1'001'157'900** |
|
| **PIG2_Mock_GDI_12** | **110818_SN365_A_s_7_1_seq_GDI-12.txt** | | **GDI-12** | **ACGGAC** | **21'060'988** | | **2'106'098'800** |
|
| **PIG2_Lena_GDI_12** | **110818_SN365_A_s_7_1_seq_GDI-13.txt** | | **GDI-13** | **CGTTAA** | **49'839'962** | | **4'983'996'200** |
|
| **PIG2_LV_GDI_12** | **110818_SN365_A_s_7_1_seq_GDI-14.txt** | | **GDI-14** | **GACTCT** | **26'331'788** | | **2'633'178'800** |
|
| **PIG3_Mock_GDI_15** | **110818_SN365_A_s_7_1_seq_GDI-15.txt** | | **GDI-15** | **TTGTTC** | **21'362'922** | | **2'136'292'200** |
|
| **PIG3_Lena_GDI_15** | **110818_SN365_A_s_7_1_seq_GDI-16.txt** | | **GDI-16** | **AGAAGA** | **32'978'890** | | **3'297'889'000** |
|
| **PIG3_LV_GDI_15** | **110818_SN365_A_s_7_1_seq_GDI-17.txt** | | **GDI-17** | **GAGGAT** | **40'544'844** | | **4'054'484'400** |
|
| **Total** |  | |  |  | **127'014'675** | | **12'701'467'500** |
| **B. RNA-seq reads eliminated after quality evaluation by FASTQC and Sickle** | | | | | | | |
| **sample name** | **Library name** | | **Real reads number in trimmed file (/4)** | | **Reads number in raw files** | **Percentage of eliminated reads** | |
| **Lena** | **trimmed_110818_SN365_A_s_7_2_seq_GDI-10.txt** | | **9155435** | | **10734717** | **0.147119109** | |
| **Lena** | **trimmed_110818_SN365_A_s_7_2_seq_GDI-13.txt** | | **21197404** | | **24919981** | **0.149381213** | |
| **Lena** | **trimmed_110818_SN365_A_s_7_2_seq_GDI-16.txt** | | **14010532** | | **16489445** | **0.150333319** | |
| **LV** | **trimmed_110818_SN365_A_s_7_2_seq_GDI-11.txt** | | **8527283** | | **10011579** | **0.148257932** | |
| **LV** | **trimmed_110818_SN365_A_s_7_2_seq_GDI-14.txt** | | **11288147** | | **13165894** | **0.142622066** | |
| **LV** | **trimmed_110818_SN365_A_s_7_2_seq_GDI-17.txt** | | **17456669** | | **20272422** | **0.138895737** | |
| **Mock** | **trimmed_110818_SN365_A_s_7_2_seq_GDI-12.txt** | | **9013184** | | **10208682** | **0.117106008** | |
| **Mock** | **trimmed_110818_SN365_A_s_7_2_seq_GDI-15.txt** | | **9132277** | | **10681461** | **0.145034841** | |
| **Mock** | **trimmed_110818_SN365_A_s_7_2_seq_GDI-9.txt** | | **8731723** | | **10530494** | **0.170815443** | |
| **Total** |  | | **217025308** | |  |  | |
| **C. RNA-Seq sequence reads mapping to pig genome (Build Sus_scrofa.Sscrofa10.2.71) using TopHat v2.0.8 algorithm** | | | | | | | |
|  | **GDI-9** | **GDI-10** | **GDI-12** | **GDI-13** | **GDI-15** | **GDI-16** | **GDI-17** |
| mapped | 86.77 | 83.75 | 87.36 | 85.55 | 85.92 |  | 72.87 |
| unmapped | 0.13 | 0.16 | 0.13 | 0.14 | 0.14 |  | 0.27 |
